# Dicarboxylic Acid Monoesters in β- and δ-Lactam Synthesis

**DOI:** 10.3390/molecules27082469

**Published:** 2022-04-11

**Authors:** Anna Ananeva, Olga Bakulina, Dmitry Dar’in, Grigory Kantin, Mikhail Krasavin

**Affiliations:** 1 Institute of Chemistry, Saint Petersburg State University, 26 Universitetskii Prospect, 198504 Peterhof, Russia; ananjewa.anna98@yandex.ru (A.A.); d.dariin@spbu.ru (D.D.); kanting@mail.ru (G.K.); 2 Institute of Living Systems, Immanuel Kant Baltic Federal University, 236041 Kaliningrad, Russia

**Keywords:** beta-lactams, delta-lactams, regioselectivity, imines, carboxylic acid activation, cyclization

## Abstract

A *N*-(2-methoxy-2-oxoethyl)-*N*-(phenylsulfonyl)glycine monomethyl ester of the respective dicarboxylic acid was involved in a reaction with imines promoted by acetic anhydride at an elevated temperature. Instead of the initially expected δ-lactam products of the Castagnoli–Cushman-type reaction, medicinally important 3-amino-2-azetidinones were obtained as the result of cyclization, involving a methylene group adjacent to an acid moiety. In contrast, replacing alcohol residue with hexafluoroisopropyl in the same substrate made another methylene group (adjacent to the ester moiety) more reactive to furnishing the desired δ-lactam in the Castagnoli–Cushman fashion.

## 1. Introduction

**Figure 1:**
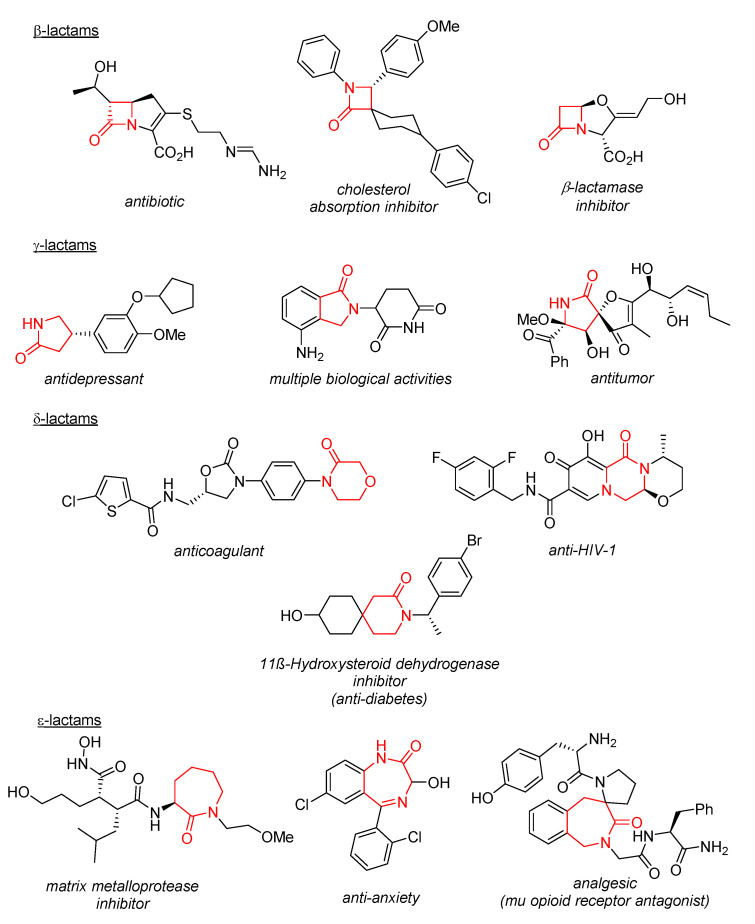
Selected examples of biologically active compounds comprising lactams rings of various sizes.

Lactams of various ring sizes (β [1], γ [2], δ [3], ε [4], denoting 4-7-membered rings, respectively, and larger [5]) represent one of the most important heterocyclic moieties employed in medicinal chemistry and drug design [6]. While the size of the lactam ring has a strong bearing on the chemical and physicochemical properties [7] of the lactam-containing compounds as well as their specific biological activity profile [8], the broadly defined lactam chemical class can be confidently defined as privileged [9], i.e., capable of delivering compounds endowed with diverse biological activities (Figure 1). This mandates the development and constant broadening of the current arsenal of synthetic methods to access lactam scaffolds, all with feasible substitution patterns for *de novo* biological target interrogation and the subsequent medicinal chemistry optimization of the hits emerging from biological screening campaigns.

Aside from conventional methods of building the lactam core, such as intramolecular amine *N*-acylation (lactamization) [10], intramolecular amide *N*-alkylation [11], carbon–carbon-bond-forming reactions [12], various annulation approaches [13,14], the oxidation of cyclic amines [15] and reduction of cyclic imides [16], the reaction of cyclic C-H anhydrides **1** with imines, dubbed the Castagnoli–Cushman reaction [17], represents an efficient way to access 5- to 7-membered [4,18] polysubstituted lactams, often in a diastereoselective fashion [19]. 

**Scheme 1:**
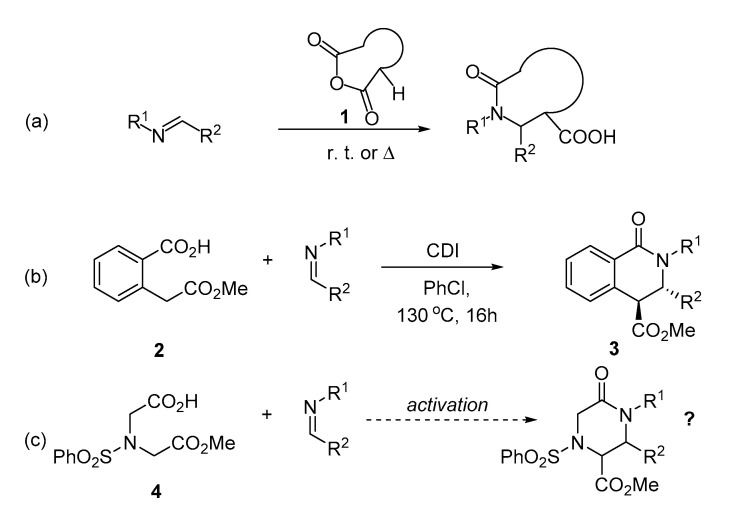
(**a**) The Castagnoli–Cushman reaction; (**b**) The use of HPA monoester in the Castagnoli–Cushman reaction [20]; (**c**) Reaction investigated in this work and initially proposed structure of the reaction product.

Recently, we demonstrated that, in the well-known synthesis of tetrahydroisoquinolonic acids from imines and homophthalic anhydride (HPA), the latter can be efficiently replaced with 2-(2-methoxy-2-oxoethyl)benzoic acid (**2**) (HPA monoester) activated by CDI (1,1′-carbonyldiimidazole). This allowed tetrahydroisoquinolonic esters **3** to be obtained and the Castagnoli–Cushman chemistry reaction to be planted onto a new reagent space that did not require the use of cyclic anhydrides [20]. Encouraged by this finding, we proceeded to investigate if the same approach could be extended to dicarboxylic acid monoesters, such as **4**, X = NSO_2_Ph (Scheme 1). Herein, we report our findings obtained during the course of this investigation.

## 2. Results and Discussion

Our initial attempt to transfer the reaction conditions optimized for the reaction of HPA monoester **2** with imines (Scheme 1b) was not successful. Therefore, we screened for a suitable carboxylic acid activation regimen that would lead to an appreciable conversion and product yield (Table 1). Among the three other activators tried (oxalyl chloride, trifluoroacetic anhydride and acetic anhydride), only acetic anhydride gave the full conversion of **4** and the best isolated yield for the product (66%, entry 4), whose molecular weight and spectral characteristics appeared to correspond to the desired δ-lactam product **5a** and demonstrated the predominant formation of one product diastereomer. Notably, the reaction turned out to be rather sensitive to thermal activation with a sharp decrease in the yield either when raising (entry 5) or reducing (entries 6–7) the temperature. Replacing chlorobenzene with other solvents (entries 8–9) did not improve the yields, while adding a base (entry 10) significantly diminished it.

**Scheme 2:**
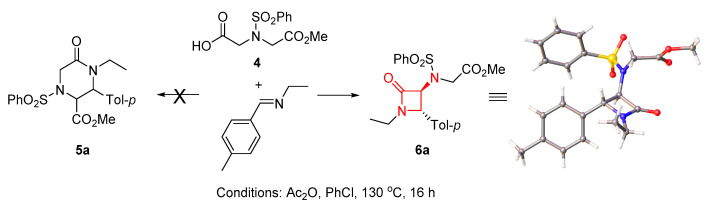
The actual outcome of the reaction of monoester **4** with (*E*)-*N*-ethyl-1-(*p*-tolyl)methanimine.

However, a single-crystal X-ray analysis of the reaction product demonstrated, to our surprise, that it was not the expected δ-lactam **5a** but rather the *trans*-diastereomer of β-lactam product **6a** (Scheme 2). While the activation of α-C-H carboxylic acids toward such a reaction by acetic anhydride (in the presence of triethylamine) has been described in the literature [21,22], the formation of the β-lactam in the absence of any base (as in our case) is hitherto undescribed. Moreover, the fact that the use of the base is detrimental to the product yield (*vide supra*) makes this transformation rather unique.

**Scheme 3:**
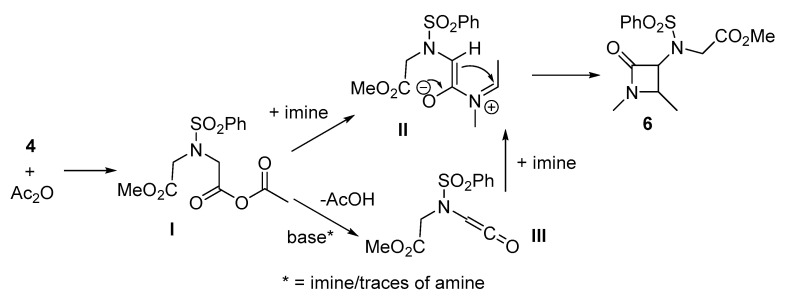
Plausible mechanistic pathways for the reaction of mono ester **4**, imines and acetic anhydride.

Two possible reaction pathways can be suggested for this observed transformation (Scheme 3). They both begin with a carboxylic acid activation by acetic anhydride and the formation of mixed anhydride **I**. This intermediate can then either directly acylate imine to form intermediate **II** or be converted into a ketene **III** via AcOH elimination (the latter can be base-promoted by imine or traces of amine from imine decomposition). Ketene **III** also reacts with imine to provide the intermediate **II**. The last step is a Mannich-type cyclization involving the methylene group adjacent to carbonyl, resulting in formation of a C-C bond and beta-lactam cycle. The selective formation of β-lactams over δ- can be explained by the increased CH acidity of the methylene group closest to the positively charged iminium fragment of intermediate **II** compared to the second methylene group adjacent to an ester moiety.

**Figure 2:**
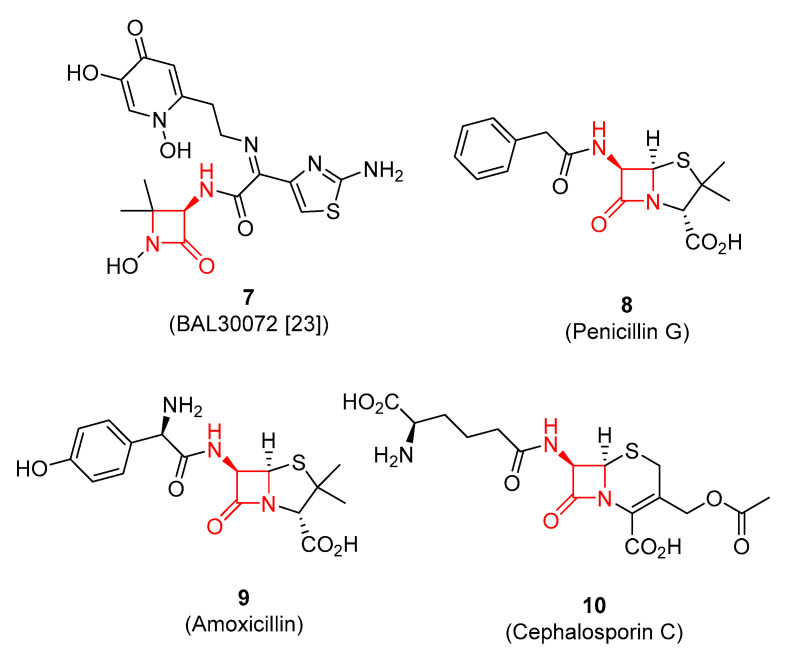
Examples of antibiotics comprising 3-amino-2-azetidinone scaffold.

In addition to the novelty of the discovered protocol for β-lactam synthesis, the medicinal importance of the 3-amino-2-azetidinone scaffold comprised by compound **6a** is relatively clear. Indeed, it is the core of exemplary antibiotics, such as **7**–**10** [23] (Figure 2). This motivated us to explore the scope of the new protocol for β-lactams **6** preparation from monoester **4** and various imines using the optimized conditions (Ac_2_O, PhCl, 130 °C, 16 h).

**Scheme 4:**
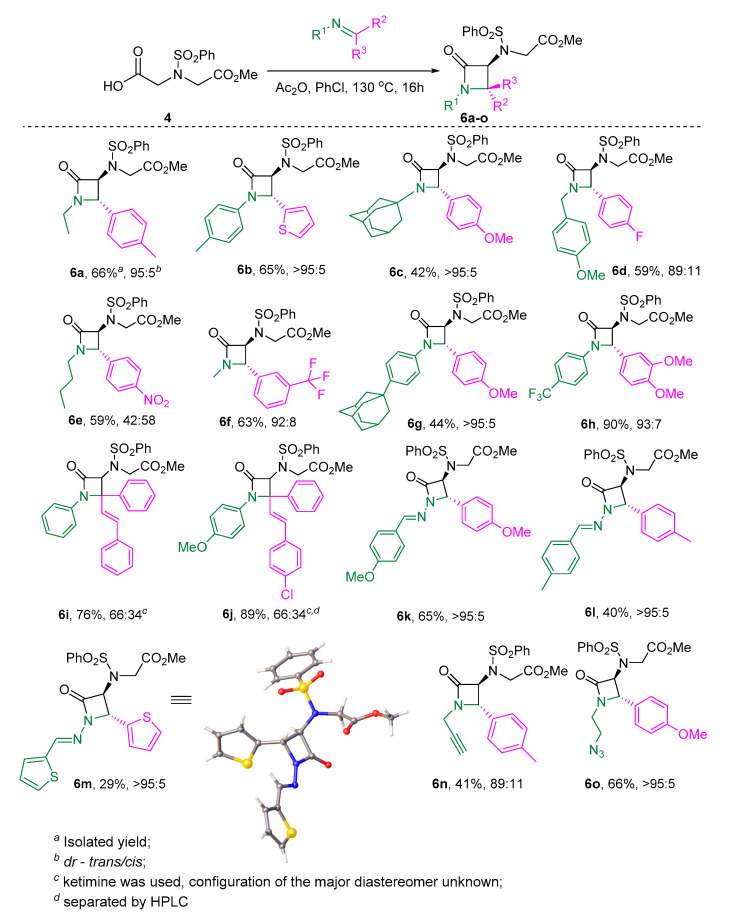
The developed novel protocol for synthesis of polysubstituted 3-amino-2-azetidinones **6a**–**o**.

Following from the results presented in Scheme 4, polysubstituted 3-amino-2-azetidinones **6a**–**o** can be synthesized in a modest-to-high yield (up to 90%) and high *trans*-diastereoselectivity (see also Table S1) in case of aldimines (R^3^ = H), as confirmed by a single-crystal X-ray analysis of two reaction products (**6a** and **6m**). The reaction appeared to work equally well for aldimines derived from both aliphatic and aromatic amines. Symmetrical hydrazones could also be productively involved in the reaction (cf., products **6k**–**m**). In the case of (*E*)-chalcones, the diastereoselectivity of the reaction deteriorated; however, this was not a detriment to the product yield. The respective diastereomers were separated by HPLC and characterized (compound **6j**). The notable products are **6n** and **6o**, which can be viewed as building blocks for further structural complexity buildup via alkyne-azide click chemistry.

**Scheme 5:**
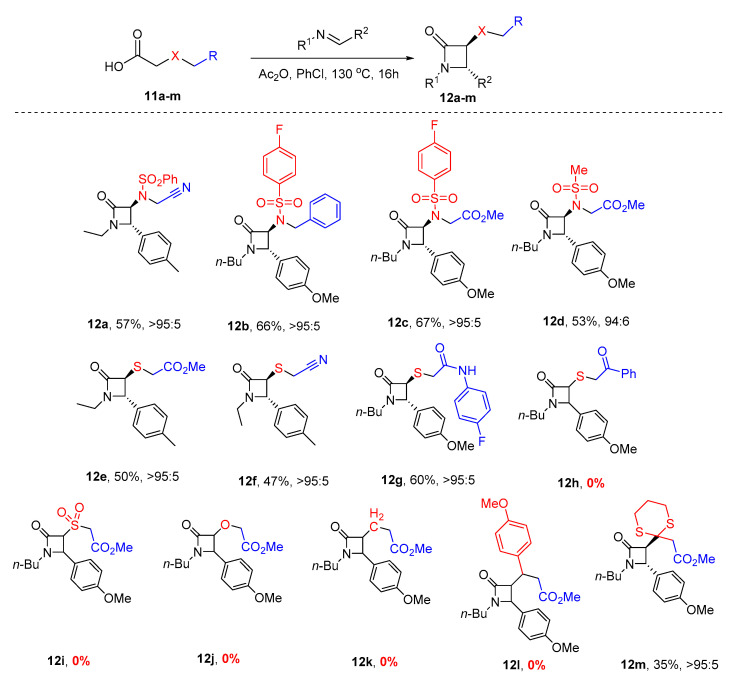
The developed novel protocol for synthesis of polysubstituted 3-amino-2-azetidinones **12**.

Encouraged by the results obtained with dicarboxylic acid monoester **4**, we proceeded to investigate the workability of the new base-free protocol for other carboxylic acids **11a**–**m** in combination with aldimines, aiming to obtain β-lactams **12** (Scheme 5). 

It turned out that the sulfonylamino-substituted acetic acids **11a**–**d** were similarly effective in the β-lactam synthesis, furnishing the respective products **12a**–**d** with good yields and a high diastereoselectivity. When replacing the nitrogen group (X = N) with sulfur, sulfone, oxygen, and carbon linkers had a strong effect on the reaction outcome. The thia-linked carboxylic acids (**11e**–**11g**) gave β-lactams in good to fair yields except for, surprisingly, the benzoyl-substituted substrate **11h**. Sulfone-, oxygen- and carbon-linked substrates did not deliver the desired products, except for 1,3-dithiane substrate **11m,** which furnished the diastereomerically pure product **12m** in a modest yield. 

**Scheme 6:**
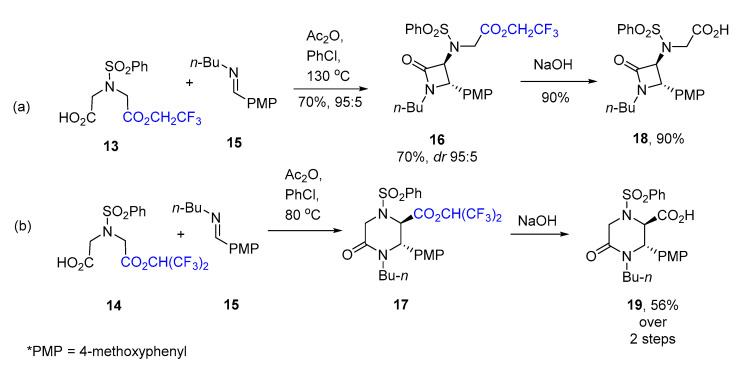
TFE (**a**) and HFIP (**b**) monoesters **13** and **14** in Ac_2_O-promoted reactions with imine **15**.

Having explored the formation of β-lactams from dicarboxylic monoester **4** and carboxylic acids **11a**–**m**, we continued pondering the possibility of forcing substrates such as **4** to react in the Castagnoli–Cushman fashion and furnish δ-lactam products. One possibility we considered would be to increase the C-H acidity of the ‘ester arm’ of the substrate, thereby making the closure of the six-membered ring more feasible. This could be achieved by placing electron-withdrawing substituents, such as perfluoroalkyl group, in the ester moiety. In our previous work on the surrogate Castagnoli–Cushman reaction of HPA monomethyl ester **2** and its analogs, we observed a notable increase in the reactivity of the 2,2,2-trifluoroethyl (TFE) ester compared to **2 [20]**. Similarly, the TFE ester was found to be more reactive towards nucleophilic addition–elimination [24]. Moreover, TFE esters have been used as versatile acylation reagents in various reactions, including transesterification [25], amidation [26], and the kinetic resolution of aliphatic amines [27]. We reasoned that, aside from TFE analog of monoester **4**, the hexafluoroisopropyl (HFIP) congener [28] would be even more predisposed to react with the Castagnoli–Cushman (rather than the previously observed) fashion and yield δ-lactams. Hence, we prepared monoesters **13** (TFE) and **14** (HFIP) (see Supplementary Information) and reacted them with *N*-butyl-1-(4-methoxyphenyl)methanimine (**15**) in chlorobenzene in the presence of acetic anhydride (Scheme 6). Expectedly, **14** proved more reactive towards imine **15** compared to its TFE counterpart (**13**) as it required a lower temperature for the reaction to be completed. Both ester products (**16** and labile **17**) were hydrolyzed to their respective carboxylic acids (**18** and **19**), and the spectral characteristics of the latter two compounds were compared to each other to reveal that compound **16** was the product of β-lactam synthesis, while compound **17** was the desired δ-lactam formed via the Castagnoli–Cushman reaction (Scheme 5). Indeed, the signals of the vicinal methine protons in **18** resonated differently (doublets at 4.81 and 4.40 ppm) compared to the same signals in **19** (doublets at 5.06 and 4.85 ppm). Small *^3^J* coupling constants (2.0–2.2 Hz) were indicative of the *trans*-configuration of both carboxylic acids [29].

**Figure 3:**
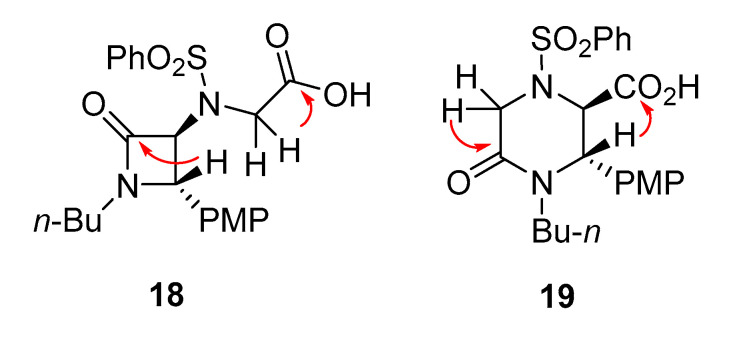
Key HMBC correlations (red arrows) displayed by compounds **18** and **19**.

Considering the importance of the notable reactivity switch between monoesters **13** and **14** (β-lactam synthesis vs. Castagnoli–Cushman δ-lactam synthesis), we continued scrutinizing the differences in the structures of products **18** and **19** using NMR spectroscopy after our attempts to obtain crystals suitable for X-ray crystallography failed. The unequivocal difference between these compounds was identified in their correlational HMBC spectra. Specifically, compound **18** displayed two key correlations, *^3^J* [CH-CON] and *^3^J* [CH_2_-CO_2_H], in contrast to compound **19,** where *^3^J* [CH_2_-CON] and *^3^J* [CH-CO_2_H] were observable (Figure 3).

**Scheme 7:**
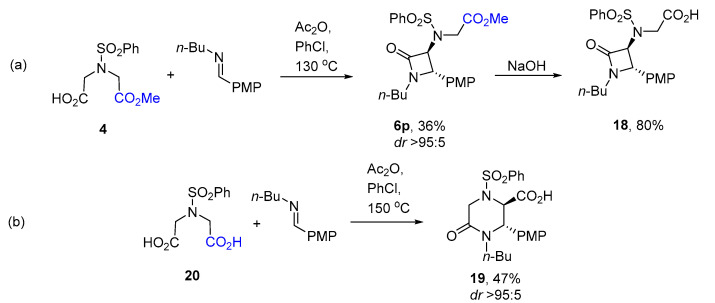
Alternative synthesis of compounds (**a**) **18** and (**b**) **19**.

Finally, we reasoned that β-lactam carboxylic acid **18** could be synthesized via the reaction of monomethyl ester **4** followed by the hydrolysis of ester **6p**. Similarly, δ-lactam carboxylic acid **19** could be obtained via the Castagnoli–Cushman reaction of dicarboxylic acid **20** mediated by the in situ generation of the respective cyclic anhydride [18] (Scheme 7). To our delight, this synthetic strategy indeed led to the compounds whose spectral characteristics fully matched those of compounds **18** and **19** synthesized as described in Scheme 6.

## 3. Conclusions

With the aim of involving a dicarboxylic acid monomethyl ester in the recently described Ac_2_O-promoted Castagnoli–Cushman-type reaction, we identified a novel protocol for β-lactam synthesis instead. A series of 25 novel compounds were prepared in 35–90% yields, mostly as single *trans*-diastereomers as confirmed by X-ray analysis. The type of substituent in the β-position in the carboxylic acid group of the monoester was found to be crucial for the reaction outcome, while the variation in the imine component was well-tolerated. Additionally, it was discovered that replacing the monomethyl ester with its hexafluoroisopropyl congener not only led to a reduction in the reaction temperature, but also to a marked reactivity switch as the reaction proceeded along the Castagnoli–Cushman-type pathway and furnished the respective δ-lactam. An investigation of the scope of the latter reaction is currently underway in our laboratories and will be reported on in due course.

## 4. Materials and Methods

NMR spectra were acquired with 400 MHz Bruker Avance III spectrometer (400.13 MHz for ^1^H, 376.49 MHz for ^19^F and 100.61 MHz for ^13^C) or 500 MHz Bruker Avance III (500.03 MHz for ^1^H and 125.73 MHz for ^13^C) in CDCl_3_ or DMSO-*d_6_* and were referenced to residual solvent proton signals (δ_H_ = 7.26 and 2.50, respectively) and solvent carbon signals (δ_C_ = 77.16 and 39.52, respectively). Multiplicities are abbreviated as follows: s = singlet, d = doublet, t = triplet, q = quartet, m = multiplet, br = broad, dd = doublet of doublets, dt = doublet of triplets, ddd = doublet/doublets of doublets; coupling constants, *J*, are reported in Hz. Mass spectra were acquired with an HRMS-ESI-qTOF spectrometer Nexera LCMS-9030 or MaXis II Bruker Daltonic GmbH (electrospray ionization mode, positive ions detection). IR spectrums were recorded with Fourier transform infrared Shimadzu spectrophotometer IRAffinity-1. Flash column chromatography on silica (Merck, 230–400 mesh) was performed with Biotage Isolera Prime instrument. TLC was performed on aluminium-backed pre-coated plates (0.25 mm) with silica gel 60 F254 with a suitable solvent system and was visualized using UV fluorescence. Preparative HPLC was carried out in a compact preparative system ECOM ECS28P00, equipped with spectrophotometric detector or Shimadzu LC-20AP. Column: YMC-Pack SIL-06, 5 µm, 250 × 20 mm or Agilent Zorbax prepHT XDB-C18, 5 μm, 21.2 × 150 mm. Chlorobenzene was distilled from P_2_O_5_ and stored in molecular sieves 4 Å (>24 h). 2-(Benzenesulfonyl-(cyanomethyl)amino)acetic acid (**11a**) was obtained from commercial sources. Synthesis of other starting materials is reported in ESI. *CCDC 2,154,010* (**6a**) and *2,154,011* (**6m**) contain the supplementary crystallographic data for this paper. These data can be obtained free of charge from The Cambridge Crystallographic Data Centre via http://www.ccdc.cam.ac.uk (accessed on 22 February 2022).

### 4.1. General Procedure for Preparation of Beta Lactams **6a**–**p**, **12a**–**m**, **16** and Their Analytical Data

In a screw-cap vial equipped with a magnetic stir bar, imine (1.1 eq) and the corresponding substituted monocarboxylic acid (0.05–1.3 mmol) were mixed in chlorobenzene (0.058 M, 1–6 mL). Then, acetic anhydride (1.1 eq) was added. The resulting mixture was placed in a pre-heated to 130 °C oil bath or metal heating block. After 16h, the mixture was cooled to room temperature, and the solvent was evaporated. The residue was purified by column chromatography in silica gel with a linear gradient (5–75%) of acetone in hexane (total volume of eluent, 400 mL) to provide pure compounds, **6a**–**p** and **12a**–**m**.

#### 4.1.1. Methyl *N*-((3RS,4RS)-1-Ethyl-2-oxo-4-(p-tolyl)azetidin-3-yl)-*N*-(phenylsulfonyl)glycinate (**6a**)

Yield 96 mg, 66%; dr 95:5, yellow oil. ^1^H NMR (500 MHz, CDCl_3_) δ 7.65–7.62 (m, 2H), 7.57–7.51 (m, 1H), 7.41 (dd, *J* = 8.3, 7.4 Hz, 2H), 7.41 (dd, *J* = 8.3, 7.4 Hz, 2H), 7.21 (d, *J* = 8.6 Hz, 2H), 4.85 (d, *J* = 1.8 Hz, 1H), 4.41 (d, *J* = 1.0 Hz, 1H), 4.26 (d, *J* = 18.3 Hz, 1H), 4.15 (d, *J* = 18.3 Hz, 1H), 3.69 (s, 3H), 3.54–3.44 (m, 1H), 2.83 (dqd, *J* = 14.3, 7.2, 0.9 Hz, 1H), 2.38 (s, 3H), 1.07 (t, *J* = 7.3 Hz, 3H). ^13^C NMR (126 MHz, CDCl_3_) δ 169.7, 163.7, 138.7, 138.3, 133.2, 132.7, 129.6, 129.1, 127.5, 126.9, 71.6, 62.5, 52.4, 48.0, 35.4, 21.3, 12.6. HRMS (ESI) *m*/*z*: [M + Na]^+^ Calcd for C_21_H_23_N_2_O_5_NaS^+^ 439.1298; found 439.1307. 

#### 4.1.2. Methyl *N*-((3RS,4SR)-2-Oxo-4-(thiophen-2-yl)-1-(p-tolyl)azetidin-3-yl)-*N*-(phenylsulfonyl)glycinate (**6b**)

Yield 107 mg, 65%; dr > 95:5, dark orange solid. ^1^H NMR (500 MHz, CDCl_3_) δ 7.70 (dd, *J* = 8.4, 1.3 Hz, 2H), 7.62–7.55 (m, 1H), 7.46 (dd, *J* = 8.3, 7.4 Hz, 2H), 7.34 (dd, *J* = 5.0, 3.0 Hz, 1H), 7.29 (dd, *J* = 3.0, 1.3 Hz, 1H), 7.17 (d, *J* = 8.4 Hz, 2H), 7.09–7.03 (m, 3H), 5.43 (d, *J* = 2.1 Hz, 1H), 4.63 (d, *J* = 2.1 Hz, 1H), 4.31 (d, *J* = 18.4 Hz, 1H), 4.16 (d, *J* = 18.4 Hz, 1H), 3.67 (s, 3H), 2.27 (s, 3H). ^13^C NMR (126 MHz, CDCl_3_) δ 169.7, 160.8, 138.3, 137.4, 134.7, 134.4, 133.5, 129.8, 129.3, 127.7, 127.2, 125.7, 123.7, 117.8, 71.5, 59.5, 52.6, 48.2, 21.1. HRMS (ESI) *m*/*z*: [M + H]^+^ Calcd for C_23_H_23_N_2_O_5_S_2_^+^ 471.1043; found 471.1050.

#### 4.1.3. Methyl *N*-((2RS,3RS)-1-(Adamantan-1-yl)-2-(4-methoxyphenyl)-4-oxoazetidin-3-yl)-*N*-(phenylsulfonyl)glycinate (**6c**)

Yield 80 mg, 42%; dr > 95:5, yellow oil. ^1^H NMR (400 MHz, CDCl_3_) δ 7.64–7.55 (m, 2H), 7.53–7.47 (m, 1H), 7.39 (t, *J* = 7.8 Hz, 2H), 7.26 (d, *J* = 8.3 Hz, 2H), 6.86 (d, *J* = 8.8 Hz, 2H), 5.10 (d, *J* = 4.9 Hz, 1H), 4.88 (d, *J* = 4.9 Hz, 1H), 3.96 (d, *J* = 18.5 Hz, 1H), 3.88 (d, *J* = 18.5 Hz, 1H), 3.82 (s, 3H), 3.48 (s, 3H), 1.98 (br.s, 3H), 1.92 (br.s, 6H), 1.62–1.51 (m, 6H). ^13^C NMR (101 MHz, CDCl_3_) δ 168.9, 163.4, 159.8, 139.2, 132.9, 129.5, 128.8, 128.4, 127.9, 113.8, 65.7, 60.6, 56.0, 55.3, 52.2, 49.3, 40.9, 36.1, 29.2. HRMS (ESI) *m*/*z*: [M + H]^+^ Calcd for C_29_H_35_N_2_O_6_S^+^ 539.2210; found 539.2219.

#### 4.1.4. Methyl *N*-((2RS,3RS)-2-(4-Fluorophenyl)-1-(4-methoxybenzyl)-4-oxoazetidin-3-yl)-*N*-(phenylsulfonyl)glycinate (**6d**)

Yield 105 mg, 59%; dr 89:11, yellow oil. ^1^H NMR (500 MHz, CDCl_3_) δ 7.64–7.58 (m, 2H), 7.54 (t, *J* = 7.5 Hz, 1H), 7.40 (t, *J* = 7.9 Hz, 2H), 7.17 (dd, *J* = 8.7, 5.3 Hz, 2H), 7.06 (t, *J* = 8.6 Hz, 2H), 7.00 (d, *J* = 8.6 Hz, 2H), 6.80 (d, *J* = 8.7 Hz, 2H), 4.72 (d, *J* = 14.8 Hz, 1H), 4.62 (d, *J* = 1.9 Hz, 1H), 4.46 (d, *J* = 1.5 Hz, 1H), 4.22 (d, *J* = 18.4 Hz, 1H), 4.05 (d, *J* = 18.4 Hz, 1H), 3.78 (s, 3H), 3.68 (d, *J* = 14.8 Hz, 1H), 3.60 (s, 3H).^13^C NMR (126 MHz, CDCl_3_) δ 169.6, 163.8, 163.0 (d, *J* = 247.7 Hz), 159.4, 138.4, 133.4, 131.4 (d, *J* = 3.2 Hz), 130.0, 129.2, 128.9 (d, *J* = 8.2 Hz), 127.7, 126.8, 116.1 (d, *J* = 21.6 Hz), 114.3, 72.0, 61.6, 55.4, 52.5, 47.9, 44.3. ^19^F NMR (376 MHz, CDCl_3_) δ-112.7. HRMS (ESI) *m*/*z*: [M + H]^+^ Calcd for C_26_H_26_FN_2_O_6_S^+^ 513.1490; found 513.1494.

#### 4.1.5. Methyl *N*-((2RS,3RS)-1-Butyl-2-(4-nitrophenyl)-4-oxoazetidin-3-yl)-*N*-(phenylsulfonyl)glycinate (**6e**)

Yield 98 mg, 59%; dr 42:58, dark orange oil. ^1^H NMR (500 MHz, CDCl_3_) δ 8.28 (d, *J* = 8.7 Hz, 2H), 8.25 (d, *J* = 8.7 Hz, 2H), 7.71–7.67 (m, 2H), 7.64 (dd, *J* = 8.5, 1.3 Hz, 2H), 7.62–7.54 (m, 4H), 7.53–7.50 (m, 2H), 7.49–7.47 (m, 2H), 7.46–7.42 (m, 2H), 5.26 (d, *J* = 4.7 Hz, 1H), 5.08 (d, *J* = 1.9 Hz, 1H), 4.97 (d, *J* = 4.8 Hz, 1H), 4.35 (d, *J* = 1.8 Hz, 1H), 4.26 (d, *J* = 18.4 Hz, 1H), 4.16 (d, *J* = 18.4 Hz, 1H), 3.99 (d, *J* = 18.3 Hz, 1H), 3.79 (d, *J* = 18.3 Hz, 1H), 3.72 (s, 3H), 3.61–3.49 (m, 2H), 3.48 (s, 3H), 2.94 (ddd, *J* = 13.8, 7.4, 6.2 Hz, 1H), 2.81–2.72 (m, 1H), 1.55–1.39 (m, 4H), 1.33–1.26 (m, 6H), 0.88 (q, *J* = 7.3 Hz, 6H). ^13^C NMR (126 MHz, CDCl_3_) δ 170.0, 168.4, 163.6, 163.3, 148.3, 148.3, 143.5, 141.8, 138.3, 138.2, 133.6, 133.5, 129.5, 129.4, 129.1, 128.0, 127.5, 127.4, 124.3, 123.8, 72.2, 68.1, 62.8, 62.1, 52.7, 52.3, 49.2, 48.2, 41.3, 40.9, 29.6, 29.3, 20.3, 20.2, 13.6, 13.6. HRMS (ESI) *m*/*z*: [M + H]^+^ Calcd for C_22_H_26_N_3_O_7_S^+^ 476.1486; found 476.1487.

#### 4.1.6. Methyl *N*-((3RS,4RS)-1-Methyl-2-oxo-4-(3-(trifluoromethyl)phenyl)azetidin-3-yl)-*N*-(phenylsulfonyl)glycinate (**6f**)

Yield 101 mg, 63%; dr 92:8, light yellow oil. ^1^H NMR (500 MHz, CDCl_3_) δ 7.67–7.61 (m, 3H), 7.60–7.47 (m, 4H), 7.45–7.37 (m, 2H), 4.91 (d, *J* = 1.9 Hz, 1H), 4.43 (d, *J* = 0.9 Hz, 1H), 4.27 (d, *J* = 18.4 Hz, 1H), 4.14 (d, *J* = 18.3 Hz, 1H), 3.71 (s, 3H), 2.77 (s, 3H). ^13^C NMR (126 MHz, CDCl_3_) δ 169.9, 164.0, 138.3, 136.8, 133.5, 131.5 (q, *J* = 32.5 Hz), 130.5, 129.7, 129.3, 127.6, 125.9 (q, *J* = 3.6 Hz), 125.0, 124.0 (q, *J* = 272.6 Hz), 123.7 (q, *J* = 3.8 Hz), 72.8, 64.5, 52.6, 48.2, 27.4. ^19^F NMR (376 MHz, CDCl_3_) δ-62.6. HRMS (ESI) *m*/*z*: [M + H]^+^ Calcd for C_20_H_20_F_3_N_2_O_5_S^+^ 457.1040; found 457.1041.

#### 4.1.7. Methyl *N*-((2RS,3RS)-1-(4-(Adamantan-1-yl)phenyl)-2-(4-methoxyphenyl)-4-oxoazetidin-3-yl)-*N*-(phenylsulfonyl)glycinate (**6g**)

Yield 94 mg, 44%; dr > 95:5, yellow oil. ^1^H NMR (500 MHz, CDCl_3_) δ 7.70–7.66 (m, 2H), 7.60–7.54 (m, 1H), 7.48–7.40 (m, 2H), 7.28 (d, *J* = 8.7 Hz, 2H), 7.23–7.17 (m, 4H), 6.90 (d, *J* = 8.7 Hz, 2H), 5.24 (d, *J* = 2.2 Hz, 1H), 4.65 (d, *J* = 2.2 Hz, 1H), 4.31 (d, *J* = 18.4 Hz, 1H), 4.11 (d, *J* = 18.4 Hz, 1H), 3.82 (s, 3H), 3.68 (s, 3H), 2.08–2.03 (m, 3H), 1.81 (d, *J* = 2.9 Hz, 6H), 1.79–1.73 (m, 3H), 1.72–1.66 (m, 3H). ^13^C NMR (126 MHz, CDCl_3_) δ 169.7, 161.4, 160.0, 148.1, 138.4, 134.3, 133.4, 129.2, 128.0, 127.7, 125.6, 119.9, 117.6, 114.5, 72.3, 62.9, 55.4, 52.6, 48.0, 43.2, 36.8, 36.0, 29.0. HRMS (ESI) *m*/*z*: [M + H]^+^ Calcd for C_35_H_39_N_2_O_6_S^+^ 615.2523; found 615.2520.

#### 4.1.8. Methyl *N*-((2RS,3RS)-2-(3,4-Dimethoxyphenyl)-4-oxo-1-(4-(trifluoromethyl)phenyl)azetidin-3-yl)-*N*-(phenylsulfonyl)glycinate (**6h**)

Yield 181 mg, 90%; dr 93:7, yellow oil. ^1^H NMR (500 MHz, CDCl_3_) δ 7.72–7.69 (m, 2H), 7.62–7.56 (m, 1H), 7.50 (d, *J* = 8.6 Hz, 2H), 7.48–7.44 (m, 2H), 7.37 (d, *J* = 8.5 Hz, 2H), 6.92–6.84 (m, 3H), 5.35 (d, *J* = 2.3 Hz, 1H), 4.62 (d, *J* = 2.3 Hz, 1H), 4.28 (d, *J* = 18.4 Hz, 1H), 4.12 (d, *J* = 18.4 Hz, 1H), 3.90 (s, 3H), 3.86 (s, 3H), 3.67 (s, 3H). ^13^C NMR (126 MHz, CDCl_3_) δ 169.7, 162.2, 149.7 (d, *J* = 3.9 Hz), 139.6, 138.2, 133.6, 129.3, 127.7, 127.4, 126.6 (d, *J* = 32.8 Hz), 126.5 (q, *J* = 3.6 Hz), 124.0 (d, *J* = 271.7 Hz), 119.5, 118.9, 117.8, 111.6, 109.6, 72.8, 64.0, 56.2, 56.1, 52.7, 48.4. ^19^F NMR (376 MHz, CDCl_3_) δ-62.3. HRMS (ESI) *m*/*z*: [M + H]^+^ Calcd for C_27_H_26_FN_2_O_7_S^+^ 579.1407; found 579.1411.

#### 4.1.9. Methyl (E)-*N*-(4-Oxo-1,2-diphenyl-2-styrylazetidin-3-yl)-*N*-(phenylsulfonyl)glycinate (**6i**)

Yield 29 mg, 76%; dr 66:34, dark orange oil. ^1^H NMR (500 MHz, CDCl_3_) δ 7.90–7.79 (m, 2H), 7.66 (d, *J* = 7.4 Hz, 2H), 7.60 (td, *J* = 7.6, 1.6 Hz, 2H), 7.54–7.46 (m, 3H), 7.46–7.27 (m, 26H), 7.20 (t, *J* = 8.0 Hz, 2H), 7.16–6.99 (m, 7H), 6.91 (d, *J* = 16.1 Hz, 1H), 6.70 (d, *J* = 16.2 Hz, 1H), 6.65 (d, *J* = 16.2 Hz, 1H), 5.28 (d, *J* = 1.0 Hz, 1H), 4.97 (s, 1H), 4.36 (d, *J* = 18.5 Hz, 1H), 4.04 (d, *J* = 18.5 Hz, 1H), 3.73 (d, *J* = 18.5 Hz, 1H), 3.50 (s, 3H), 3.47 (s, 3H), 3.28 (d, *J* = 18.5 Hz, 1H), 2.27 (s, 6H). ^13^C NMR (126 MHz, CDCl_3_) δ 169.0, 161.2, 161.0, 139.5, 138.2, 137.8, 136.7, 136.7, 136.6, 135.9, 135.9, 135.1, 133.5, 133.3, 129.7, 129.2, 129.2, 129.1, 129.1, 129.0, 129.0, 128.9, 128.8, 128.7, 128.6, 128.6, 128.6, 128.3, 128.2, 128.1, 127.4, 127.1, 127.0, 126.3, 125.9, 124.8, 124.7, 124.0, 119.0, 118.6, 72.8, 70.9, 52.2, 52.2, 49.4, 48.1, 19.9. HRMS (ESI) *m*/*z*: [M + Na]^+^ Calcd for C_32_H_28_N_2_O_5_S^+^ 575.1611; found 575.1614.

#### 4.1.10. Methyl (E)-*N*-(2-(4-Chlorostyryl)-1-(4-methoxyphenyl)-4-oxo-2-phenylazetidin-3-yl)-*N*-(phenylsulfonyl)glycinate (**6j**)

Yield 39 mg, 89% (after flash chromatography); dr 66:34, yellow oil. This mixture was further separated by preparative HPLC on silica gel column YMC-Pack SIL-06, 5 µm, 250 × 20 mm (mobile phase hexane-acetone, 5–50% of acetone, total volume 600 mL; flow 20 mL/min, 25 °C) to afford pure diastereomers. Major isomer (16 mg): ^1^H NMR (500 MHz, CDCl_3_) δ 7.59–7.55 (m, 2H), 7.54–7.48 (m, 1H), 7.39–7.29 (m, 7H), 7.26 (d, *J* = 1.5 Hz, 4H), 7.21–7.17 (m, 2H), 7.00 (d, *J* = 16.3 Hz, 1H), 6.66 (d, *J* = 9.1 Hz, 2H), 6.52 (d, *J* = 16.2 Hz, 1H), 4.82 (s, 1H), 4.26 (d, *J* = 18.6 Hz, 1H), 3.92 (d, *J* = 18.5 Hz, 1H), 3.66 (s, 3H), 3.43 (s, 3H). ^13^C NMR (126 MHz, CDCl_3_) δ 169.0, 160.2, 156.5, 138.0, 137.7, 134.5, 134.4, 133.5, 132.2, 130.1, 129.2, 129.1, 129.1, 128.7, 128.3, 128.2, 128.1, 126.4, 125.1, 119.9, 114.3, 70.9, 55.5, 52.3, 49.4. Minor isomer (7 mg): ^1^H NMR (500 MHz, CDCl_3_) δ 7.83 (dd, *J* = 8.4, 1.3 Hz, 2H), 7.64–7.57 (m, 1H), 7.53–7.46 (m, 4H), 7.46–7.39 (m, 5H), 7.30 (s, 4H), 6.87 (d, *J* = 16.2 Hz, 1H), 6.80 (d, *J* = 9.2 Hz, 2H), 6.62 (d, *J* = 16.2 Hz, 1H), 5.23 (d, *J* = 1.0 Hz, 1H), 3.76 (s, 3H), 3.68 (d, *J* = 18.5 Hz, 1H), 3.44 (s, 3H), 3.27 (d, *J* = 18.6 Hz, 1H). ^13^C NMR (126 MHz, CDCl_3_) δ 168.5, 160.4, 156.6, 139.5, 135.1, 134.4, 134.3, 133.4, 132.0, 130.1, 129.1, 129.1, 129.0, 128.8, 128.2, 128.2, 128.2, 128.1, 120.2, 114.4, 72.7, 55.6, 52.2, 48.1. HRMS (ESI) *m*/*z*: [M + H]^+^ Calcd for C_33_H_30_ClN_2_O_6_S^+^ 617.1508; found 617.1508.

#### 4.1.11. Methyl *N*-((2RS,3RS)-1-(((E)-4-Methoxybenzylidene)amino)-2-(4-methoxyphenyl)-4-oxoazetidin-3-yl)-*N*-(phenylsulfonyl)glycinate (**6k**)

Yield 25 mg, 65%; dr > 95:5, yellow oil. ^1^H NMR (400 MHz, CDCl_3_) δ 7.66 (dd, *J* = 8.5, 1.3 Hz, 2H), 7.62 (s, 1H), 7.57 (tt, *J* = 6.9, 1.2 Hz, 1H), 7.51 (d, *J* = 8.9 Hz, 2H), 7.44 (t, *J* = 7.9 Hz, 2H), 7.28 (d, *J* = 8.7 Hz, 2H), 6.92 (d, *J* = 8.7 Hz, 2H), 6.83 (d, *J* = 8.9 Hz, 2H), 5.40 (d, *J* = 2.0 Hz, 1H), 4.44 (d, *J* = 2.1 Hz, 1H), 4.33 (d, *J* = 18.5 Hz, 1H), 4.19 (d, *J* = 18.5 Hz, 1H), 3.83 (s, 3H), 3.80 (s, 3H), 3.70 (s, 3H). ^13^C NMR (101 MHz, CDCl_3_) δ 169.9, 162.1, 160.2, 159.7, 147.7, 138.3, 133.5, 129.6, 129.3, 128.0, 127.7, 126.2, 125.9, 114.7, 114.3, 70.1, 66.6, 55.5, 55.5, 52.7, 48.1. HRMS (ESI) *m*/*z*: [M + H]^+^ Calcd for C_27_H_28_N_3_O_7_S^+^ 538.1642; found 538.1647.

#### 4.1.12. Methyl (E)-*N*-(1-((4-Methylbenzylidene)amino)-2-oxo-4-(p-tolyl)azetidin-3-yl)-*N*-(phenylsulfonyl)glycinate (**6l**)

Yield 14 mg, 40%; dr > 95:5, yellow oil. ^1^H NMR (500 MHz, DMSO-*d_6_* +CDCl_3_) δ 7.62–7.59 (m, 2H), 7.55 (t, *J* = 7.5 Hz, 1H), 7.51 (s, 1H), 7.40 (t, *J* = 7.8 Hz, 2H), 7.35 (d, *J* = 8.1 Hz, 2H), 7.13 (s, 4H), 7.06 (d, *J* = 8.1 Hz, 2H), 5.26 (d, *J* = 2.1 Hz, 1H), 4.47 (d, *J* = 2.0 Hz, 1H), 4.20 (d, *J* = 18.4 Hz, 1H), 4.05 (d, *J* = 18.4 Hz, 1H), 3.61 (s, 3H), 2.29 (s, 3H), 2.25 (s, 3H). ^13^C NMR (126 MHz, DMSO-*d_6_* +CDCl_3_) δ 168.8, 159.3, 146.9, 140.7, 138.2, 137.7, 132.9, 130.5, 129.6, 129.3, 128.8, 128.7, 127.1, 126.8, 125.8, 69.6, 65.5, 51.9, 47.3, 20.9, 20.7. HRMS (ESI) *m*/*z*: [M + H]^+^ Calcd for C_27_H_28_N_3_O_5_S^+^ 506.1744; found 506.1750.

#### 4.1.13. Methyl *N*-((3RS,4SR)-2-Oxo-4-(thiophen-2-yl)-1-(((E)-thiophen-2-ylmethylene)amino) azetidin-3-yl)-*N*-(phenylsulfonyl)glycinate (**6m**)

Yield 10 mg, 29%; dr > 95:5, brown oil. ^1^H NMR (500 MHz, CDCl_3_) δ 8.12 (s, 1H), 7.72 (dd, *J* = 8.5, 1.2 Hz, 2H), 7.64–7.56 (m, 1H), 7.47 (t, *J* = 7.9 Hz, 2H), 7.39 (dt, *J* = 5.1, 1.0 Hz, 1H), 7.37 (dd, *J* = 5.1, 1.2 Hz, 1H), 7.20 (td, *J* = 3.7, 1.4 Hz, 2H), 7.06 (dd, *J* = 5.1, 3.6 Hz, 1H), 7.01 (dd, *J* = 5.0, 3.7 Hz, 1H), 5.74 (d, *J* = 2.1 Hz, 1H), 4.57 (d, *J* = 2.0 Hz, 1H), 4.32 (d, *J* = 18.4 Hz, 1H), 4.20 (d, *J* = 18.4 Hz, 1H), 3.70 (s, 3H). ^13^C NMR (126 MHz, CDCl_3_) δ 183.1, 169.8, 159.4, 143.1, 138.2, 138.1, 137.6, 133.6, 131.8, 129.9, 129.4, 127.7, 127.7, 127.3, 126.7, 70.8, 63.3, 52.8, 48.3. HRMS (ESI) *m*/*z*: [M + H]^+^ Calcd for C_21_H_20_N_3_O_5_S_3_^+^ 490.0560; found 490.0571.

#### 4.1.14. Methyl *N*-((3RS,4RS)-2-Oxo-1-(prop-2-yn-1-yl)-4-(p-tolyl)azetidin-3-yl)-*N*-(phenylsulfonyl)glycinate (**6n**)

Yield 61 mg, 41%; dr 89:11, dark brown oil. ^1^H NMR (500 MHz, CDCl_3_) δ 7.65 (dd, *J* = 8.5, 1.3 Hz, 2H), 7.58–7.52 (m, 1H), 7.43–7.36 (m, 2H), 7.19 (q, *J* = 8.1 Hz, 4H), 4.92 (d, *J* = 2.1 Hz, 1H), 4.58 (d, *J* = 2.1 Hz, 1H), 4.33–4.24 (m, 2H), 4.10 (d, *J* = 18.4 Hz, 1H), 3.70 (s, 3H), 3.53 (ddd, *J* = 17.8, 2.5, 0.9 Hz, 1H), 2.38 (s, 3H). ^13^C NMR (126 MHz, CDCl_3_) δ 169.5, 163.9, 139.0, 138.4, 133.3, 132.0, 129.8, 129.2, 127.7, 127.0, 76.0, 73.0, 72.2, 62.6, 52.6, 48.0, 30.2, 21.4. HRMS (ESI) *m*/*z*: [M + H]^+^ Calcd for C_22_H_23_N_2_O_5_S^+^ 427.1322; found 427.1328.

#### 4.1.15. Methyl *N*-((2RS,3RS)-1-(2-Azidoethyl)-2-(4-methoxyphenyl)-4-oxoazetidin-3-yl)-*N*-(phenylsulfonyl)glycinate (**6o**)

Yield 22 mg, 66%; dr > 95:5, orange oil. ^1^H NMR (500 MHz, CDCl_3_) δ 7.66 (dd, *J* = 8.5, 1.3 Hz, 2H), 7.58–7.53 (m, 1H), 7.42 (td, *J* = 7.6, 1.7 Hz, 2H), 7.23–7.17 (m, 2H), 6.95–6.91 (m, 2H), 4.90 (d, *J* = 2.0 Hz, 1H), 4.52 (d, *J* = 1.9 Hz, 1H), 4.29 (d, *J* = 18.3 Hz, 1H), 4.14 (d, *J* = 18.3 Hz, 1H), 3.84 (s, 3H), 3.71 (s, 3H), 3.60 (ddd, *J* = 14.5, 7.2, 5.4 Hz, 1H), 3.40 (ddd, *J* = 6.8, 6.0, 5.1 Hz, 2H), 2.92 (dddd, *J* = 14.6, 6.6, 5.0, 0.7 Hz, 1H). ^13^C NMR (126 MHz, CDCl_3_) δ 169.9, 164.6, 160.3, 138.5, 133.4, 129.2, 128.4, 127.7, 127.2, 114.6, 72.3, 63.4, 55.5, 52.6, 49.0, 48.1, 39.7. HRMS (ESI) *m*/*z*: [M + H]^+^ Calcd for C_21_H_24_N_5_O_6_S^+^ 474.1442; found 474.1442.

#### 4.1.16. Methyl *N*-((2RS,3RS)-1-Butyl-2-(4-methoxyphenyl)-4-oxoazetidin-3-yl)-*N*-(phenylsulfonyl)glycinate (**6p**)

Yield 58 mg, 36%, dr > 95:5, orange oil. ^1^H NMR (400 MHz, CDCl_3_) δ 7.64–7.59 (m, 2H), 7.53 (q, *J* = 7.1 Hz, 1H), 7.40 (t, *J* = 7.8 Hz, 2H), 7.19 (d, *J* = 8.7 Hz, 2H), 6.92 (d, *J* = 8.7 Hz, 2H), 4.80 (d, *J* = 2.0 Hz, 1H), 4.40 (d, *J* = 2.0 Hz, 1H), 4.28 (d, *J* = 18.3 Hz, 1H), 4.16 (d, *J* = 18.3 Hz, 1H), 3.84 (s, 3H), 3.69 (s, 3H), 3.43 (dt, *J* = 14.6, 7.6 Hz, 1H), 2.72 (dt, *J* = 14.0, 6.7 Hz, 1H), 1.41 (p, *J* = 7.5 Hz, 2H), 1.26 (dddd, *J* = 9.9, 7.3, 5.8, 2.1 Hz, 2H), 0.85 (t, *J* = 7.2 Hz, 3H). ^13^C NMR (101 MHz, CDCl_3_) δ 169.8, 163.9, 160.1, 138.5, 133.3, 129.1, 128.4, 127.7, 127.6, 114.4, 71.8, 62.8, 55.5, 52.5, 48.1, 40.3, 29.6, 20.2, 13.6. HRMS (ESI) *m*/*z*: [M + H]^+^ Calcd for C_23_H_29_N_2_O_6_S^+^ 461.1741; found 461.1746.

#### 4.1.17. *N*-(Cyanomethyl)-*N*-((3RS,4RS)-1-ethyl-2-oxo-4-(p-tolyl)azetidin-3-yl)benzenesulfonamide (**12a**)

Yield 85 mg, 57%; dr > 95:5, dark brown oil. ^1^H NMR (400 MHz, CDCl_3_) δ 7.64–7.54 (m, 3H), 7.46–7.39 (m, 2H), 7.22 (d, *J* = 8.1 Hz, 2H), 7.10 (d, *J* = 8.1 Hz, 2H), 4.79 (d, *J* = 2.0 Hz, 1H), 4.66 (d, *J* = 18.3 Hz, 1H), 4.59 (d, *J* = 2.1 Hz, 1H), 4.29 (d, *J* = 18.5 Hz, 1H), 3.52 (dq, *J* = 14.8, 7.4 Hz, 1H), 2.92–2.80 (m, 1H), 2.41 (s, 3H), 1.10 (t, *J* = 7.3 Hz, 3H). ^13^C NMR (101 MHz, CDCl_3_) δ 162.8, 139.4, 137.1, 134.1, 132.1, 129.9, 129.6, 127.9, 127.1, 114.9, 71.2, 60.6, 35.8, 34.2, 21.4, 12.7. HRMS (ESI) *m*/*z*: [M + H]^+^ Calcd for C_20_H_22_N_3_O_3_S^+^ 384.1376; found 384.1374.

#### 4.1.18. *N*-Benzyl-*N*-((2RS,3RS)-1-butyl-2-(4-methoxyphenyl)-4-oxoazetidin-3-yl)-4-fluorobenzenesulfonamide (**12b**)

Yield 101 mg, 66%; dr > 95:5, yellow oil. ^1^H NMR (500 MHz, CDCl_3_) δ 7.89 (ddd, *J* = 8.9, 5.0, 2.5 Hz, 2H), 7.36 (dd, *J* = 7.7, 1.9 Hz, 2H), 7.33–7.26 (m, 3H), 7.15 (t, *J* = 8.6 Hz, 2H), 6.95 (d, *J* = 8.7 Hz, 2H), 6.84 (d, *J* = 8.7 Hz, 2H), 4.58 (d, *J* = 15.5 Hz, 1H), 4.53 (d, *J* = 1.7 Hz, 1H), 4.29 (d, *J* = 15.5 Hz, 1H), 4.16 (d, *J* = 2.1 Hz, 1H), 3.80 (s, 3H), 3.30 (dt, *J* = 14.5, 7.4 Hz, 1H), 2.61 (dt, *J* = 13.8, 6.7 Hz, 1H), 1.20 (q, *J* = 7.0 Hz, 2H), 1.13 (dt, *J* = 13.6, 6.9 Hz, 2H), 0.79 (t, *J* = 7.2 Hz, 3H). ^13^C NMR (126 MHz, CDCl_3_) δ 165.3 (d, *J* = 255.1 Hz), 165.0, 160.0, 136.4, 135.6 (d, *J* = 3.3 Hz), 130.4 (d, *J* = 9.4 Hz), 128.8, 128.4, 128.3, 127.8, 127.5, 116.4 (d, *J* = 22.5 Hz), 114.5, 72.6, 62.5, 55.4, 51.0, 40.2, 29.6, 20.2, 13.7. ^19^F NMR (376 MHz, CDCl_3_) δ-104.9. HRMS (ESI) *m*/*z*: [M + H]^+^ Calcd for C_27_H_30_FN_2_O_4_S^+^ 497.1905; found 497.1908.

#### 4.1.19. Methyl *N*-((2RS,3RS)-1-Butyl-2-(4-methoxyphenyl)-4-oxoazetidin-3-yl)-*N*-((2-nitrophenyl)sulfonyl)glycinate (**12c**)

Yield 214 mg, 67%; dr > 95:5, yellow oil. ^1^H NMR (400 MHz, CDCl_3_) δ 7.72–7.62 (m, 3H), 7.53 (ddd, *J* = 8.4, 6.9, 1.8 Hz, 1H), 7.24 (d, *J* = 8.7 Hz, 2H), 6.96 (d, *J* = 8.7 Hz, 2H), 4.92 (d, *J* = 1.9 Hz, 1H), 4.57 (d, *J* = 1.8 Hz, 1H), 4.45 (d, *J* = 18.7 Hz, 1H), 4.38 (d, *J* = 18.8 Hz, 1H), 3.87 (s, 3H), 3.76 (s, 3H), 3.47 (dt, *J* = 14.7, 7.6 Hz, 1H), 2.76 (dt, *J* = 13.6, 6.6 Hz, 1H), 1.51–1.38 (m, 2H), 1.36–1.23 (m, 2H), 0.89 (t, *J* = 7.3 Hz, 3H). ^13^C NMR (101 MHz, CDCl_3_) δ 170.0, 163.4, 160.3, 148.2, 134.1, 132.2, 131.9, 131.4, 128.4, 127.4, 124.6, 114.5, 71.7, 62.5, 55.5, 52.6, 48.6, 40.4, 29.6, 20.2, 13.7. HRMS (ESI) *m*/*z*: [M + H]^+^ Calcd for C_23_H_28_N_3_O_8_S^+^ 506.1592; found 506.1593.

#### 4.1.20. Methyl *N*-((2RS,3RS)-1-Butyl-2-(4-methoxyphenyl)-4-oxoazetidin-3-yl)-*N*-(methylsulfonyl)glycinate (**12d**)

Yield 94 mg, 53%; dr 94:6, light yellow oil. ^1^H NMR (500 MHz, CDCl_3_) δ 7.23 (d, *J* = 8.6 Hz, 2H), 6.92 (d, *J* = 8.7 Hz, 2H), 4.75 (d, *J* = 2.0 Hz, 1H), 4.39 (d, *J* = 1.9 Hz, 1H), 4.24 (d, *J* = 18.6 Hz, 1H), 4.09 (d, *J* = 18.6 Hz, 1H), 3.81 (s, 3H), 3.73 (s, 3H), 3.47 (dt, *J* = 14.0, 7.6 Hz, 1H), 3.05 (s, 3H), 2.75 (dt, *J* = 13.7, 6.7 Hz, 1H), 1.47–1.39 (m, 2H), 1.34–1.23 (m, 2H), 0.86 (t, *J* = 7.3 Hz, 3H). ^13^C NMR (126 MHz, CDCl_3_) δ 170.2, 164.3, 160.2, 128.0, 127.6, 114.6, 72.7, 62.8, 55.4, 52.6, 49.0, 41.3, 40.2, 29.6, 20.2, 13.7. HRMS (ESI) *m*/*z*: [M + H]^+^ Calcd for C_18_H_27_N_2_O_6_S^+^ 399.1584; found 399.1590.

#### 4.1.21. Methyl 2-(((3RS,4RS)-1-Ethyl-2-oxo-4-(p-tolyl)azetidin-3-yl)thio)acetate (**12e**)

Yield 189 mg, 50%; dr > 95:5, orange oil. ^1^H NMR (400 MHz, CDCl_3_) δ 7.20 (s, 4H), 4.46 (d, *J* = 2.1 Hz, 1H), 3.98 (d, *J* = 2.3 Hz, 1H), 3.67 (s, 3H), 3.56–3.44 (m, 2H), 3.38 (d, *J* = 15.3 Hz, 1H), 2.92 (dq, *J* = 14.3, 7.2 Hz, 1H), 2.35 (s, 3H), 1.08 (t, *J* = 7.3 Hz, 3H). ^13^C NMR (101 MHz, CDCl_3_) δ 170.5, 165.5, 139.0, 133.6, 129.8, 126.6, 62.9, 58.6, 52.6, 35.9, 32.6, 21.3, 12.9. HRMS (ESI) *m*/*z*: [M + H]^+^ Calcd for C_15_H_20_NO_3_S^+^ 294.1158; found 294.1163.

#### 4.1.22. 2-(((3RS,4RS)-1-Ethyl-2-oxo-4-(p-tolyl)azetidin-3-yl)thio)acetonitrile (**12f**)

Yield 120 mg, 47%; dr > 95:5, light brown oil. ^1^H NMR (500 MHz, CDCl_3_) δ 7.29–7.19 (m, 4H), 4.66 (d, *J* = 2.0 Hz, 1H), 4.06 (d, *J* = 1.6 Hz, 1H), 3.63–3.45 (m, 3H), 2.99 (dqd, *J* = 14.5, 7.2, 0.9 Hz, 1H), 2.39 (s, 3H), 1.14 (t, *J* = 7.3 Hz, 3H). ^13^C NMR (126 MHz, CDCl_3_) δ 164.3, 139.4, 133.0, 130.0, 126.6, 116.8, 62.4, 58.1, 36.2, 21.3, 15.6, 12.8. HRMS (ESI) *m*/*z*: [M + Na]^+^ Calcd for C_14_H_16_N_2_NaOS^+^ 283.0876; found 283.0877.

#### 4.1.23. 2-(((2RS,3RS)-1-Butyl-2-(4-methoxyphenyl)-4-oxoazetidin-3-yl)thio)-*N*-(4-fluorophenyl)acetamide (**12g**)

Yield 102 mg, 60%; dr > 95:5, light brown oil. ^1^H NMR (500 MHz, CDCl_3_) δ 9.23 (s, 1H), 7.63–7.54 (m, 2H), 7.23–7.18 (m, 2H), 7.06–6.97 (m, 2H), 6.95–6.87 (m, 2H), 4.37 (d, *J* = 2.1 Hz, 1H), 3.91 (dd, *J* = 2.1, 0.8 Hz, 1H), 3.81 (s, 3H), 3.63 (d, *J* = 15.5 Hz, 1H), 3.51–3.41 (m, 1H), 3.37 (d, *J* = 15.6 Hz, 1H), 2.85 (dt, *J* = 13.7, 6.7 Hz, 1H), 1.45 (dt, *J* = 14.5, 7.3 Hz, 2H), 1.39–1.21 (m, 2H), 0.87 (t, *J* = 7.3 Hz, 3H). ^13^C NMR (126 MHz, CDCl_3_) δ 167.2, 166.8, 160.5, 160.5, 159.5 (d, *J* = 243.2 Hz), 134.2 (d, *J* = 2.6 Hz), 127.9, 127.5, 121.7 (d, *J* = 8.0 Hz), 115.6 (d, *J* = 22.4 Hz), 114.7, 62.8, 58.3, 55.5, 41.0, 36.1, 29.6, 20.2, 13.6. ^19^F NMR (376 MHz, CDCl_3_) δ-118.1. HRMS (ESI) *m*/*z*: [M + Na]^+^ Calcd for C_22_H_25_FN_2_NaO_3_S^+^ 439.1462; found 439.1462.

#### 4.1.24. Methyl 2-(2-((2RS,3SR)-1-Butyl-2-(4-methoxyphenyl)-4-oxoazetidin-3-yl)-1,3-dithian-2-yl)acetate (**12m**)

Yield 18 mg, 35%; light yellow oil. ^1^H NMR (500 MHz, CDCl_3_) δ 7.30 (d, *J* = 8.7 Hz, 2H), 6.90 (d, *J* = 8.7 Hz, 2H), 4.87 (d, *J* = 2.2 Hz, 1H), 3.93 (d, *J* = 2.0 Hz, 1H), 3.81 (s, 3H), 3.60 (s, 3H), 3.46 (d, *J* = 14.5 Hz, 1H), 3.35 (d, *J* = 14.5 Hz, 1H), 3.08 (ddd, *J* = 14.3, 12.1, 2.0 Hz, 2H), 2.88 (dt, *J* = 14.5, 3.9 Hz, 1H), 2.76–2.62 (m, 2H), 2.12 (dtd, *J* = 13.5, 5.4, 2.9 Hz, 1H), 1.96–1.84 (m, 1H), 1.47 (ddt, *J* = 11.7, 9.7, 3.4 Hz, 2H), 1.41–1.30 (m, 2H), 0.87 (t, *J* = 7.3 Hz, 3H). ^13^C NMR (126 MHz, CDCl_3_) δ 169.2, 165.7, 159.8, 129.5, 128.3, 114.4, 65.8, 57.4, 55.4, 51.9, 49.1, 42.9, 40.1, 29.9, 26.3, 26.3, 24.5, 20.4. HRMS (ESI) *m*/*z*: [M + H]^+^ Calcd for C_21_H_30_NNaO_4_S_2_^+^ 424.1611; found 424.1615.

#### 4.1.25. 2,2,2-Trifluoroethyl *N*-((3RS,4RS)-1-butyl-2-oxo-4-(p-tolyl)azetidin-3-yl)-*N*-(phenylsulfonyl)glycinate (**16**)

Yield 109 mg, 70%; dr > 95:5, light yellow oil. ^1^H NMR (400 MHz, CDCl_3_) δ 7 7.61–7.51 (m, 3H), 7.45–7.37 (m, 2H), 7.21 (d, *J* = 8.7 Hz, 2H), 6.94 (d, *J* = 8.7 Hz, 2H), 4.77 (d, *J* = 2.0 Hz, 1H), 4.58–4.41 (m, 2H), 4.38 (d, *J* = 18.6 Hz, 1H), 4.37 (d, *J* = 1.8 Hz, 1H), 4.30 (d, *J* = 18.6 Hz, 1H), 3.85 (s, 3H), 3.43 (dt, *J* = 14.8, 7.6 Hz, 1H), 2.73 (dt, *J* = 13.6, 6.8 Hz, 1H), 1.41 (p, *J* = 7.5 Hz, 2H), 1.32–1.20 (m, 2H), 0.85 (t, *J* = 7.2 Hz, 3H). ^13^C NMR (101 MHz, CDCl_3_) δ 168.2, 163.6, 160.2, 138.0, 133.5, 129.3, 128.4, 127.6, 127.4, 122.8 (q, *J* = 277.0 Hz), 114.5, 71.4, 62.9, 61.1 (q, *J* = 37.0 Hz), 55.5, 47.8, 40.4, 29.6, 20.2, 13.6. ^19^F NMR (376 MHz, CDCl_3_) δ-73.6. HRMS (ESI) *m*/*z*: [M+H]^+^ Calcd for C_24_H_28_F_3_N_2_O_6_S^+^ 529.1615; found 529.1619.

### 4.2. N-((2RS,3RS)-1-Butyl-2-(4-methoxyphenyl)-4-oxoazetidin-3-yl)-N-(phenylsulfonyl)glycine (**18**)

Compound **16** (37 mg, 0.07 mmol) or **6p** (55 mg, 0.12 mmol) was dissolved in a mixture of MeOH and water (3 + 3 mL), followed by addition of NaOH (5 equiv.). After stirring for 24 h at room temperature, MeOH was removed in vacuo, and the residue was acidified with HCl conc to pH 1 and partitioned between water (5 mL) and ethyl acetate (10 mL). The organic layer was separated, dried over sodium sulfate, filtered and concentrated to give pure title compound. Yield 27 mg, 90% (from hydrolysis of compound **16**), light yellow oil. Yield 44 mg, 80% (from hydrolysis of compound **6p**), light yellow oil. ^1^H NMR (400 MHz, CDCl_3_) δ 7.64 (d, *J* = 7.5 Hz, 2H), 7.56 (t, *J* = 7.5 Hz, 1H), 7.42 (t, *J* = 7.8 Hz, 2H), 7.21 (d, *J* = 8.5 Hz, 2H), 6.94 (d, *J* = 8.7 Hz, 2H), 4.78 (d, *J* = 2.0 Hz, 1H), 4.39 (d, *J* = 1.8 Hz, 1H), 4.26 (d, *J* = 18.5 Hz, 1H), 4.19 (d, *J* = 18.6 Hz, 1H), 3.85 (s, 3H), 3.44 (dt, *J* = 14.7, 7.6 Hz, 1H), 2.75 (dt, *J* = 14.2, 7.0 Hz, 1H), 1.43 (p, *J* = 7.6 Hz, 2H), 1.28 (dq, *J* = 15.6, 4.3, 3.7 Hz, 2H), 0.86 (t, *J* = 7.2 Hz, 3H). ^13^C NMR (101 MHz, CDCl_3_) δ 172.0, 164.2, 160.3, 138.1, 133.5, 129.3, 128.4, 127.7, 127.4, 114.5, 71.7, 63.0, 55.5, 48.0, 40.5, 29.6, 20.3, 13.7. HRMS (ESI) *m*/*z*: [M + Na]^+^ Calcd for C_22_H_26_N_2_O_6_NaS^+^ 469.1404; found 469.1413. IR (KBr): ῦ = 1760 (vs) cm^−1^ (C=O).

### 4.3. (2RS,3SR)-4-Butyl-3-(4-methoxyphenyl)-5-oxo-1-(phenylsulfonyl)piperazine-2-carboxylic Acid (**19**)

Preparation 1 (Scheme 6b). In a screw-cap vial equipped with a magnetic stir bar, *N*-butyl-1-(4-methoxyphenyl)methanimine (40 mg, 0.21 mmol) and compound **14** (80 mg, 0.19 mmol) were mixed in chlorobenzene (5 mL). Then, acetic anhydride (21 mg, 0.21 mmol) was added. The resulting mixture was placed in a bath pre-heated to 80 °C. After 16 h, the reaction mixture was cooled to RT and concentrated. The residue was dissolved in a mixture of MeOH and water (6 + 6 mL) followed by the addition of NaOH (19 mg, 0.49 mmol, 5 equiv.). After stirring for 24 h at room temperature, MeOH was removed in vacuo and the residue was diluted with water (5 mL) and extracted with ethyl acetate (5 mL). The aqueous layer was separated and acidified with HCl conc to pH 1 and extracted with ethyl acetate (2 × 10 mL). Combined organic layers were dried over sodium sulfate, filtered and concentrated to give pure compound **19**. Yield 45 mg, 56%, dr > 95:5, light orange oil.

^1^H NMR (400 MHz, CDCl_3_) δ 7.50 (tt, *J* = 7.0, 1.4 Hz, 1H), 7.41 (dd, *J* = 8.5, 1.4 Hz, 2H), 7.37–7.29 (m, 2H), 7.00 (d, *J* = 8.7 Hz, 2H), 6.80 (d, *J* = 8.7 Hz, 2H), 5.07 (d, *J* = 2.3 Hz, 1H), 4.84 (d, *J* = 2.1 Hz, 1H), 4.24 (d, *J* = 17.1 Hz, 1H), 4.06 (d, *J* = 17.1 Hz, 1H), 3.90 (dt, *J* = 13.6, 7.6 Hz, 1H), 3.83 (s, 3H), 2.58 (dt, *J* = 13.9, 7.1 Hz, 1H), 1.51–1.34 (m, 2H), 1.31–1.12 (m, 2H), 0.85 (t, *J* = 7.3 Hz, 3H). ^13^C NMR (101 MHz, CDCl_3_) δ 171.7, 165.0, 160.0, 138.4, 133.0, 129.0, 129.0, 127.5, 127.3, 114.7, 61.2, 61.2, 55.5, 46.2, 45.7, 29.1, 20.0, 13.8. HRMS (ESI) *m*/*z*: [M + Na]^+^ Calcd for C_22_H_26_N_2_O_6_NaS^+^ 469.1404; found 469.1411. IR (KBr): ῦ = 1738 (s), 1611 (s) cm^−1^ (C=O).

Preparation 2 (Scheme 7b). A mixture of *N*-(phenylsulfonyl)iminodiacetic acid (123 mg, 0.45 mmol), *N*-butyl-1-(4-methoxyphenyl)methanimine (95 mg, 0.5 mmol) and acetic anhydride (50 mg, 0.5 mmol) in PhCl (1 mL) was placed in a pre-heated bath (150 °C) and stirred for 16h. After cooling to room temperature, the reaction mixture was partitioned between ethyl acetate (5 mL) and saturated sodium bicarbonate solution (5 mL). The aqueous layer was separated, cooled to 0 °C and acidified with HCl concentrated to pH 1, followed by extraction with ethyl acetate (2 × 5 mL). Combined organic layers were dried over sodium sulfate, filtered and concentrated to give pure compound **19**. Yield 90 mg, 47%; dr > 95:5, light orange oil. 

## Figures and Tables

**Table 1:** Condition findings for the reaction of monoester **4** with (*E*)-*N*-ethyl-1-(*p*-tolyl)methanimine.

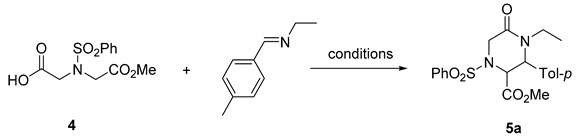				
Entry	Reagent	T (°C)	Solvent	Yield (% *^a,b^*)
1	CDI	130	PhCl (chlorobenzene)	0
2	(COCl)_2_	130/25	PhCl	0
3	(CF_3_CO)_2_O	130	PhCl	0
4	Ac_2_O	80	PhCl	0
5		110	PhCl	36
6		130	PhCl	66, 64 *^c^*
7		150	PhCl	26
8		130	1,4-dioxane	66
9		130	*o*-xylene	51
10		130	PhCl	23 *^d^*

*^a^* Reactions were conducted with ratio **4**:imine:activating agent = 1/1.1/1.1, at 0.06 M concentration using dry solvent. *^b^* Isolated yields. *^c^* Conducted with 1.3 equiv. of imine and 1.3 equiv. of Ac_2_O. *^d^* 1 equiv. of *N*,*N*-diisopropylethylamine was added.

## Data Availability

Not applicable.

## Supplementary Materials

The following supporting information can be downloaded at: www.mdpi.com/article/10.3390/molecules27082469/s1. Synthesis of starting materials and their analytical data, crystallographic data, copies of NMR spectra, Table S1 (diastereomeric ratios from crude reaction mixtures and after purification). References [30,31,32,33,34,35,36] are cited in the supplementary materials.
