# Supplementary material for: Dicarboxylic Acid Monoesters in β- and δ-Lactam Synthesis
Source: Molecules. 2022 Apr 11;27(8):2469. doi: 10.3390/molecules27082469 (PMC9032910; doi:10.3390/molecules27082469)
Supplement: Supplementary file 1 [file molecules-27-02469-s001.zip › molecules-1662283-supplementary.pdf]

ELECTRONIC SUPPORTING INFORMATION FOR

Dicarboxylic acid monoesters in reactions with imines

Anna Ananeva [1], Olga Bakulina [1*], Dmitry Dar'in [1], Grigory Kantin [1] and Mikhail Krasavin [1,2*]

[1] Saint Petersburg State University, 26 Universitetskii prospect, Peterhof 198504 Russian Federation
1; ananjewa.anna98@yandex.ru (A.A..), o.bakulina@spbu.ru (O.B.), d.dariin@spbu.ru (D.D.), kanting@mail.ru (G.K.)
[2] [b]Immanuel Kant Baltic Federal University, Kaliningrad 236041 Russian Federation; m.krasavin@spbu.ru (M.K.)
* Correspondence: o.bakulina@spbu.ru (O.B.), m.krasavin@spbu.ru (M.K.)

E-mail: m.krasavin@spbu.ru

Table of contents

General information 2

General procedure for preparation of starting materials and their analytical data: 2

Synthesis of compounds 13 and 14, general scheme 6

General procedure for preparation of *Tert-butyl N-(2-((1,1,1,3,3,3-hexafluoropropan-2-yl)oxy)-2-oxoethyl)-N-(phenylsulfonyl)glycinate (S3)* and *Tert-butyl N-(2-oxo-2-(2,2,2-trifluoroethoxy)ethyl)-N-(phenylsulfonyl)glycinate (S4)*....................7

General procedure for preparation of *N-(2-Oxo-2-(2,2,2-trifluoroethoxy)ethyl)-N-(phenylsulfonyl)glycine (13)* and *N-(2-((1,1,1,3,3,3-Hexafluoropropan-2-yl)oxy)-2-oxoethyl)-N-(phenylsulfonyl)glycine (14)*........8

Table S1. 9

Crystallographic data 13

References 14

Copies of ^{1}H and ^{13}C NMR spectra 15

General information

NMR spectra were acquired with 400 MHz Bruker Avance III spectrometer (400.13 MHz for ^{1}H and 100.61 MHz for ^{13}C) in $CDCl_3$ or DMSO-d_6 and were referenced to residual solvent proton signals (δ_H = 7.26 and 2.50, respectively) and solvent carbon signals (δ_C = 77.16 and 39.52, respectively). Mass spectra were acquired with HRMS-ESI-qTOF spectrometer Nexera LCMS-9030 or MaXis II Bruker Daltonic GmbH (electrospray ionization mode, positive ions detection). Flash column chromatography on silica (Merck, 230-400 mesh) was performed with Biotage Isolera Prime instrument. TLC was performed on aluminium-backed pre-coated plates (0.25 mm) with silica gel 60 F254 with a suitable solvent system and was visualized using UV fluorescence. Preparative HPLC was carried out on compact preparative system ECOM ECS28P00, equipped with spectrophotometric detector or Shimadzu LC-20AP. Column: YMC-Pack SIL-06, 5 μm, 250×20 mm or Agilent Zorbax prepHT XDB-C18, 5 μm, 21.2×150 mm. Chlorobenzene was distilled from P_2O_5 and stored over molecular sieves 4Å (>24h). 2-(Benzenesulfonyl-(cyanomethyl)amino)acetic acid **(11a)** was obtained from commercial sources.

General procedure for preparation of starting materials and their analytical data:

***N-(2-Methoxy-2-oxoethyl)-N-(phenylsulfonyl)glycine* (4)**

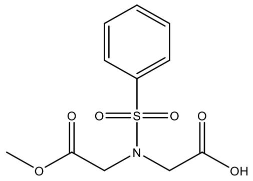

To the mixture of 2,2'-((phenylsulfonyl)azanediyl)diacetic acid[1] (1g, 3.7 mmol) in ethyl acetate (30 mL) was added trifluoroacetic anhydride (1.9 g, 9.25 mmol) in a round-bottom flask. The reaction mixture was left stirring at room temperature for one day and then concentrated under reduced pressure. The resulting light yellow semi-solid was mixed with methanol (15 mL) and left stirring at room temperature for 1 day. After completion, the reaction mixture was concentrated under reduced pressure and the pure title product was obtained in 97% yield (1 g) as white solid. ^{1}H NMR (400 MHz, DMSO-d_6) δ 7.81 (d, J = 7.5 Hz, 2H), 7.66 (t, J = 7.3 Hz, 1H), 7.57 (t, J = 7.6 Hz, 2H), 4.17 (s, 2H), 4.05 (s, 2H), 3.53 (s, 3H). ^{13}C NMR (101 MHz, DMSO-d_6) δ 169.8, 169.0, 139.5, 132.9, 129.1, 126.9, 51.8, 48.4, 48.4. HRMS (ESI) m/z: $[M+Na]^+$ Calcd for $C_{11}H_{13}SNO_6^+$ 310.0356; Found 310.0357

N-Benzyl-N-((4-fluorophenyl)sulfonyl)glycine (11b)

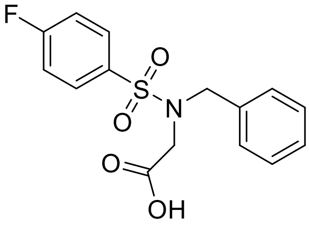

Preparation and NMR data in accordance with[2].

N-(2-Methoxy-2-oxoethyl)-N-((2-nitrophenyl)sulfonyl)glycine (11c)

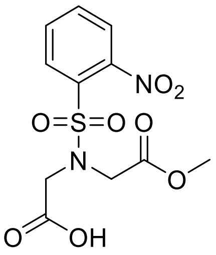

Preparation and NMR data in accordance with[3].

N-(2-Methoxy-2-oxoethyl)-N-(methylsulfonyl)glycine (11d)

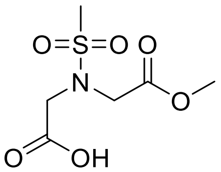

To the mixture of 2,2'-((methylsulfonyl)azanediyl)diacetic acid[4] (0.5 g, 2.4 mmol) in ethyl acetate (15 mL) was added trifluoroacetic anhydride (1.2 g, 5.9 mmol) in a round-bottom flask. The reaction mixture was left stirring at room temperature for one day and then concentrated under reduced pressure. The resulting light yellow semi-solid was mixed with methanol (8 mL) and left stirring at room temperature for 1 day. After completion, the reaction mixture was concentrated under reduced pressure and the pure title product was obtained in 97% yield (0.52 g) as white solid. ^{1}H NMR (400 MHz, DMSO-d_6+ $CDCl_3$) δ 11.93 (s, 1H), 4.08 (s, 2H), 4.02 (s, 2H), 3.66 (s, 3H), 2.94 (s, 3H). ^{13}C NMR (101 MHz, DMSO-d_6+ $CDCl_3$) δ 170.2, 169.0, 51.8, 48.1, 48.0, 40.0. HRMS (ESI) m/z: $[M+H]^+$ Calcd for $C_6H_{12}NNaSO_6^+$ 226.0380; Found 226.0355

2-((2-Methoxy-2-oxoethyl)thio)acetic acid (11e)

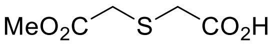

To a solution of 2,2'-thiodiacetic acid (500 mg, 3.33 mmol) in ethyl acetate (27 mL) in a round-bottom flask trifluoroacetic anhydride (1.8 g, 8.33 mmol) was added in one portion. The reaction mixture was left stirring at room temperature for one day and then concentrated under reduced pressure. The resulting crude anhydride was mixed with dry DCM (3 mL) and then added

dropwise to a stirred mixture of triethylamine (674 mg, 6.66 mmol) and methanol (25 mL) at room temperature. After 1 day the mixture was concentrated under reduced pressure, followed by addition of DCM (20 mL) and water (20 mL). The pH of aqueous layer was adjusted to 1 with concentrated HCl under cooling with ice. The aqueous layer was extracted with DCM (3×20 mL), then combined organic phase was washed with water, dried over Na_2SO_4 and evaporated to give pure title compound. Yield 25% (62 mg). ^{1}H NMR (400 MHz, $CDCl_3$) δ 9.64 (s, 1H), 3.74 (s, 3H), 3.43 (s, 2H), 3.41 (s, 2H). ^{13}C NMR (101 MHz, $CDCl_3$) δ 175.5, 170.5, 52.7, 46.0, 33.68, 33.63. HRMS (ESI) m/z: $[M+Na]^+$ Calcd for $C_5H_8SNaO_4^+$ 187.0036; Found 187.0039

2-((Cyanomethyl)thio)acetic acid (11f)

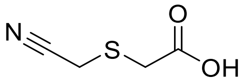

Preparation and NMR data in accordance with[5]

2-((2-((4-Fluorophenyl)amino)-2-oxoethyl)thio)acetic acid (11g)

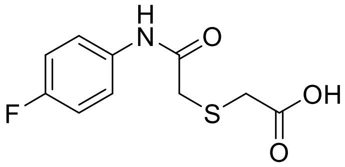

In a 100 ml Erlenmeyer flask, NaOH (920 mg, 23 mmol) was dissolved in water (30 mL) and 2-mercaptoacetic acid (0.73 mL, 10.5 mmol) was added to the resulting mixture. A solution of 2-chloro-*N*-(4-fluorophenyl)acetamide (1.9 g, 10 mmol) in MeOH (15 mL) was further added to the mixture. The resulting solution was heated with a reflux condensor at 45-50 °C. After that, the mixture was evaporated by half of the volume and then extracted with $CHCl_3$ (3×5 mL). The aqueous water layer was then acidified to pH = 1 and a second liquid phase was formed, which solidified in the refrigerator upon standing. Then resulting solid was filtered, washed with water and hexane. The obtained white powder was dried at 70°C. Yield 63% (1.5 g). ^{1}H NMR (400 MHz, DMSO-d_6+ $CDCl_3$) δ 12.42 (s, 1H), 9.91 (s, 1H), 7.58 – 7.47 (m, 2H), 6.94 (t, J = 8.9 Hz, 2H), 3.36 (s, 2H), 3.36 (s, 2H). ^{13}C NMR (101 MHz, DMSO-d_6+ $CDCl_3$) δ 171.0, 167.0, 158.1 (d, J = 241.6 Hz), 134.7 (d, J = 2.7 Hz), 120.8 (d, J = 7.7 Hz), 114.7 (d, J = 22.0 Hz), 35.7, 33.7. ^{19}F NMR (376 MHz, DMSO-d_6+ $CDCl_3$) δ -118.95. HRMS (ESI) m/z: $[M+Na]^+$ Calcd for $C_{10}H_{10}FNNaSO_3^+$ 266.0258; Found 266.0261

2-(2-(2-Methoxy-2-oxoethyl)-1,3-dithian-2-yl)acetic acid (11m)

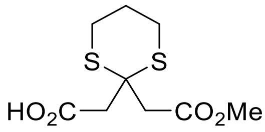

Acetic anhydride (0.6 mL was added to 2,2'-(1,3-dithiane-2,2-diyl)diacetic acid (100 mg, 0.42 mmol) in a round-bottom flask equipped with magnetic stir bar and a rubber septum. The mixture was heated to 40°C in an oil bath under stirring for 1 day. The resulting orange-colored solution was concentrated under reduced pressure to give pale pink crystals which were used in the next stage directly.

The resulting crude anhydride was mixed with dry DCM (5 mL) and added dropwise to the stirred mixture of triethylamine (85 mg, 0.84 mmol) and methanol (5 mL) at room temperature. After 1 day the reaction mixture was concentrated under reduced pressure and diluted with DCM (20 mL) and water (20 mL). The pH of aqueous layer was adjusted to 1 with concentrated HCl under cooling with ice. The aqueous layer was extracted with DCM (3×20 mL), then combined organic phase was washed with water, dried over Na_2SO_4 and evaporated to give the title product. Yield 50% (52 mg), yellow oil. ^1H NMR (400 MHz, $CDCl_3$) δ 3.72 (s, 3H), 3.37 (s, 2H), 3.32 (s, 2H), 2.94 – 2.89 (m, 4H), 2.04 – 1.95 (m, 2H). 13C NMR (126 MHz, $CDCl_3$) δ 173.5, 169.9, 52.1, 46.3, 42.3, 42.2, 26.6, 24.4. HRMS (ESI) m/z: $[M+Na]^+$ Calcd for $C_9H_{14}S_2NaO_4^+$ 273.0226; Found 273.0232

Synthesis of compounds 13 and 14, general scheme

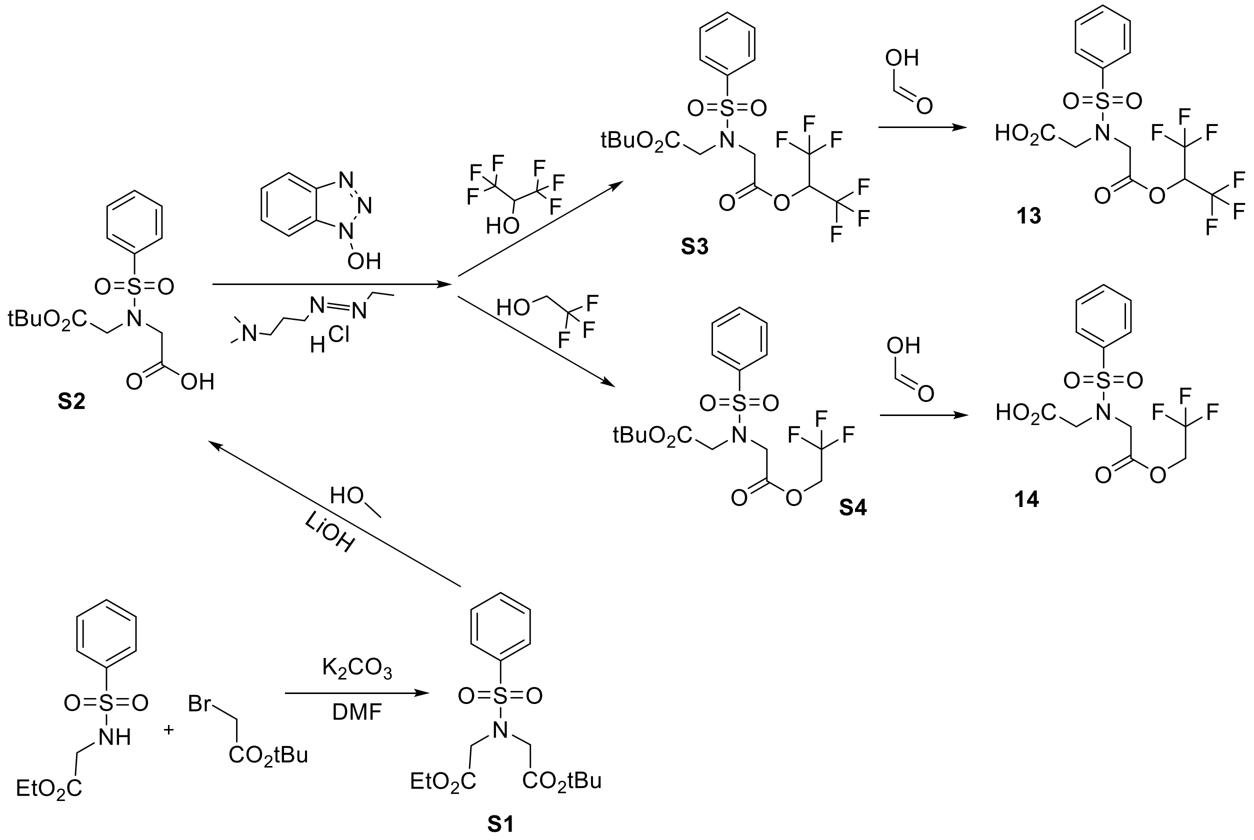

Tert-butyl N-(2-ethoxy-2-oxoethyl)-N-(phenylsulfonyl)glycinate (S1)

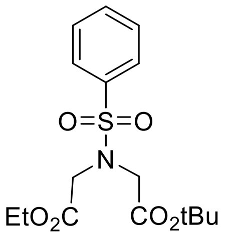

A solution of ethyl (phenylsulfonyl)glycinate (2 g, 8.2 mmol) in DMF was treated with K_2CO_3 (3.3 g, 23.7 mmol) and tert-butyl bromoacetate (1.9 g, 9.9 mmol). The reaction mixture was stirred at 35 °C. After 5 h (TLC control), the resulting yellow suspension was cooled to RT and diluted with H_2O and EtOAc. The organic phase was separated, washed with brine, dried over Na_2SO_4, filtered and the solvent was removed under reduced pressure. Yield 86% (2.5 g). ^{1}H NMR (400 MHz, $CDCl_3$) δ 7.92 – 7.78 (m, 2H), 7.63 – 7.53 (m, 1H), 7.52 – 7.45 (m, 2H), 4.20 (s, 2H), 4.17 – 4.02 (m, 4H), 1.38 (s, 9H), 1.21 (t, J = 7.2 Hz, 3H). ^{13}C NMR (101 MHz, $CDCl_3$) δ 168.8, 167.7, 139.9, 132.8, 128.9, 127.3, 82.4, 61.4, 48.9, 48.4, 27.9, 14.1. HRMS (ESI) m/z: $[M+Na]^+$ Calcd for $C_{16}H_{23}NNaO_6S^+$ 380.1138; Found 380.1140

N-(2-(tert-butoxy)-2-oxoethyl)-N-(phenylsulfonyl)glycine (S2)

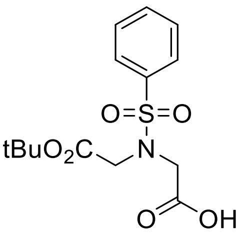

Tert-butyl *N*-(2-methoxy-2-oxoethyl)-N-(phenylsulfonyl)glycinate **S1** (2.2 g, 6.12 mmol) was dissolved in CH_3OH (60 mL). The resulting solution was cooled to 0 °C and slowly poured into a cold solution of LiOH (7.35 mmol, 176.4 mg) in H_2O (60 mL) and then left stirring at room temperature overnight. After evaporation of MeOH in vacuo, the aqueous layer was acidified with CH_3COOH to pH = 3-4, and extracted with EtOAc (3×50 mL). The combined organic phase was washed with water (3×100 mL), dried over Na_2SO_4, filtered and concentrated. The resulting solid was ground with a hexane/Et_2O mixture (5%), filtered and dried in vacuo. Yield is 56% (1.1 g). ^1H NMR (400 MHz, DMSO-d_6) δ 12.75 (s, 1H), 7.88 – 7.78 (m, 2H), 7.71 – 7.61 (m, 1H), 7.62 – 7.52 (m, 2H), 4.05 (d, J = 5.3 Hz, 4H), 1.29 (s, 9H). ^{13}C NMR (101 MHz, DMSO-d_6) δ 169.9, 167.4, 139.7, 132.8, 129.1, 126.9, 81.2, 49.0, 48.4, 27.5. HRMS (ESI) m/z: $[M+Na]^+$ Calcd for $C_{14}H_{19}NNaO_6S^+$ 352.0825; Found 352.0827

General procedure for preparation of *Tert-butyl N-(2-((1,1,1,3,3,3-hexafluoropropan-2-yl)oxy)-2-oxoethyl)-N-(phenylsulfonyl)glycinate (S3)* and *Tert-butyl N-(2-oxo-2-(2,2,2-trifluoroethoxy)ethyl)-N-(phenylsulfonyl)glycinate (S4)*

N-(2-(tert-butoxy)-2-oxoethyl)-N-(phenylsulfonyl)glycine **S2** (1 equiv., 300 mg, 0.9 mmol) was dissolved in dichloromethane (0.1 M, 9 mL) and cooled to 0 °C under stirring. EDCI (1.2 equiv., 209 mg, 1.1 mmol), HOBt (1.2 equiv., 147.7 mg, 1.1 mmol) and 1,1,1,3,3,3-hexafluoroisopropanol (10 eq., 1.5 g, 9.1 mmol) or 1,1,1-trifluoroethanol (910 mg, 9.1 mmol) were then added to the reaction mixture. After 30 minutes at 0 °C the ice bath was removed and stirring was continued overnight at room temperature. The solvent was removed in vacuo, and the residue was dissolved in ethyl acetate, washed with 1N HCl and sat. $NaHCO_3$ (3×10 mL). The organic phase was dried over sodium sulfate, filtered and concentrated in vacuo to give pure title product.

***Tert*-butyl *N*-(2-oxo-2-(2,2,2-trifluoroethoxy)ethyl)-N-(phenylsulfonyl)glycinate (S3)**

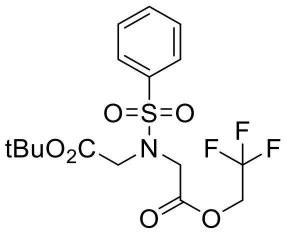

Yield: 74% (278 mg). ^1H NMR (400 MHz, $CDCl_3$) δ 7.84 (dt, J = 7.0, 1.4 Hz, 2H), 7.64 – 7.55 (m, 1H), 7.54 – 7.40 (m, 1H), 4.45 (q, J = 8.4 Hz, 2H), 4.34 (s, 2H), 4.09 (s, 2H), 1.38 (s, 9H). ^{13}C NMR (101 MHz, $CDCl_3$) δ 167.5, 167.4, 139.4, 133.0, 129.1, 127.3, 122.6 (d, J = 277.4 Hz), 82.7, 60.8 (q, J = 37.1 Hz), 48.8, 48.1, 27.9. ^{19}F NMR (376 MHz, $CDCl_3$) δ -73.71. HRMS (ESI) m/z: $[M+Na]^+$ Calcd for $C_{16}H_{20}F_3NaNO_6S^+$ 434.0856; Found 434.0860

***Tert*-butyl *N*-(2-((1,1,1,3,3,3-hexafluoropropan-2-yl)oxy)-2-oxoethyl)-N-(phenylsulfonyl)glycinate (S4).**

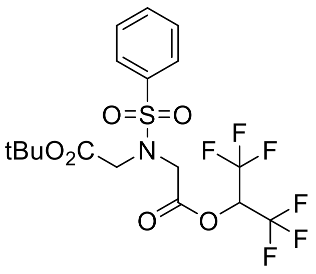

Yield: 81% (352 mg). ^{1}H NMR (400 MHz, $CDCl_3$) δ 7.88 – 7.78 (m, 2H), 7.64 – 7.55 (m, 1H), 7.56 – 7.47 (m, 2H), 5.70 (hept, *J* = 6.1 Hz, 1H), 4.45 (s, 2H), 4.07 (s, 2H), 1.38 (s, 9H). ^{13}C NMR (101 MHz, $CDCl_3$) δ 167.4, 166.5, 139.3, 133.3, 129.3, 127.4, 120.2 (q, *J* = 284.4 Hz), 83.1, 67.4 (sept, *J* = 35.1 Hz), 48.9, 48.0, 28.0. ^{19}F NMR (376 MHz, $CDCl_3$) δ -73.17. HRMS (ESI) m/z: $[M+K]^+$ Calcd for $C_{17}H_{19}F_6KNO_6S^+$ 518.0469; Found 518.0474

General procedure for preparation of *N-(2-Oxo-2-(2,2,2-trifluoroethoxy)ethyl)-N-(phenylsulfonyl)glycine (13)* and *N-(2-((1,1,1,3,3,3-Hexafluoropropan-2-yl)oxy)-2-oxoethyl)-N-(phenylsulfonyl)glycine (14)*

Compound **S3** or **S4** obtained from the previous stage was dissolved in formic acid (1mL). After stirring for 16h at room temperature, the mixture was concentrated in vacuo and partitioned between EtOAc (20 mL) and water (10 mL). The organic phase was dried over sodium sulfate, filtered and concentrated in vacuo to obtain the crude product, which was purified by column chromatography using gradient elution with mobile phase CH_2Cl_2/EtOAc, 1-25% of EA.

N-(2-Oxo-2-(2,2,2-trifluoroethoxy)ethyl)-N-(phenylsulfonyl)glycine (13)

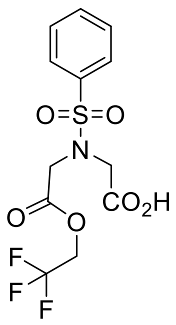

Yield 140 mg, 37%, yellow oil. ^{1}H NMR (400 MHz, DMSO-d_6) δ 12.80 (s, 1H), 7.87 – 7.78 (m, 2H), 7.71 – 7.62 (m, 1H), 7.60 – 7.52 (m, 2H), 4.70 (q, *J* = 9.1 Hz, 2H), 4.32 (s, 2H), 4.06 (s, 2H). ^{13}C NMR (101 MHz, DMSO-d_6) δ 169.6, 167.5, 139.3, 133.0, 129.1, 126.9, 123.2 (d, *J* = 277.5 Hz), 60.2 (q, *J* = 35.4 Hz), 48.3, 48.2. ^{19}F NMR (376 MHz, DMSO-d_6) δ -72.2. HRMS (ESI) m/z: $[M+H]^+$ Calcd for $C_{12}H_{13}SF_3NO_6^+$ 356.0410; Found 356.0415.

N-(2-((1,1,1,3,3,3-Hexafluoropropan-2-yl)oxy)-2-oxoethyl)-N-(phenylsulfonyl)glycine (14)

Yield 188 mg, 43%, yellow solid. ^{1}H NMR (400 MHz, $CDCl_3$+DMSO-d_6) ^{1}H NMR (400 MHz, DMSO-d_6) δ 7.86 – 7.67 (m, 2H), 7.60 – 7.44 (m, 1H), 7.48 – 7.31 (m, 2H), 5.62 (pd, J = 6.0, 1.5 Hz, 1H), 4.40 (s, 2H), 4.06 (d, J = 11.4 Hz, 2H). ^{13}C NMR (101 MHz, $CDCl_3$+DMSO-d_6) δ169.6, 165.9, 138.7, 132.7, 128.7, 126.8, 120.5 (q, J = 278.3 Hz), 67.9 – 64.2 (m), 47.6, 47.3. ^{19}F NMR (376 MHz, $CDCl_3$+DMSO-d_6) δ -73.2. HRMS (ESI) m/z: [M+Na]+ Calcd for $C_{13}H_{11}SF_6NNaO_6^+$ 446.0103; Found 446.0107

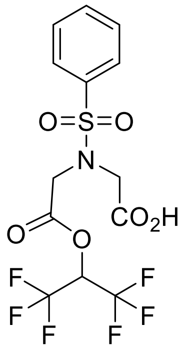

Table S1. Diastereomeric ratios from crude reaction mixtures and after purification. Selected ^{1}H NMR characteristics of H-3 protons of corresponding isomers were analyzed:

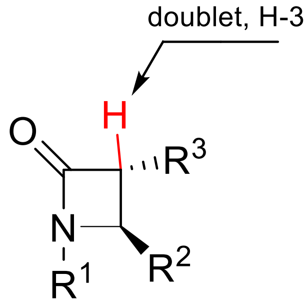

major isomer = isomer 1

Compound	R	Isomer 1. δ, ppm (J, Hz)	Isomer 2. δ, ppm (J, Hz)	Dr (isomer 1:2)
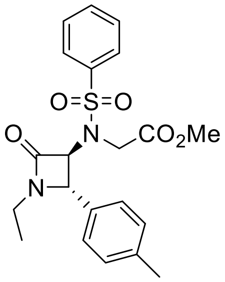	I[a]	4.85 (4.9)	5.25 (1.8)	95:5
	II[b]	4.85 (4.9)	5.25 (1.9)	95:5
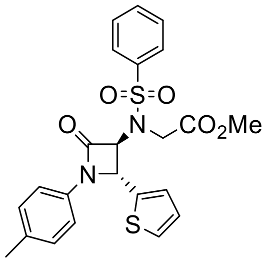	I	5.43 (2.1)	5.63 (2.3)	>95:5
	II	5.43 (2.2)	5.63 (2.1)	>95:5
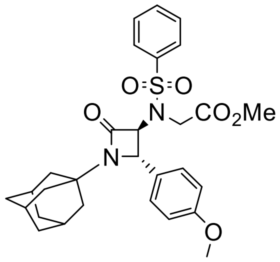	I	5.11 (4.9)	-	>95:5
	II	5.10 (4.9)	-	>95:5

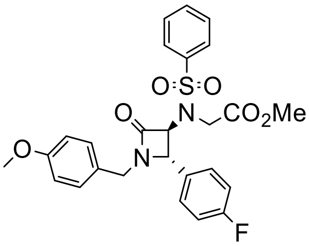	I	4.63 (2.1)	5.17 (4.7)	89:11
	II	4.62 (2.1)	5.17 (4.9)	86:14
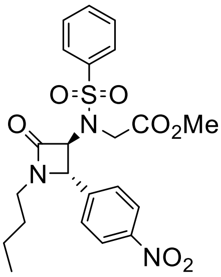	I	5.09 (2.0)	5.27 (4.7)	42:58
	II	5.08 (1.9)	5.26 (4.8)	42:58
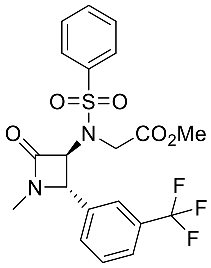	I	4.92 (2.0)	5.31 (4.6)	92:8
	II	4.91 (2.0)	5.3 (4.7)	92:8
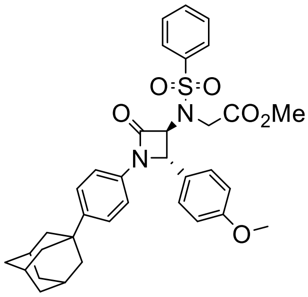	I	5.24 (2.3)	5.41 (5.2)	>95:5
	II	5.24 (2.2)	5.41 (5.1)	>95:5
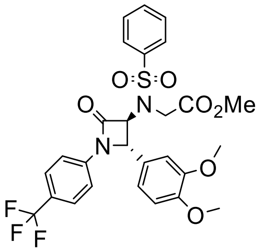	I	5.36 (2.4)	5.46 (5.6)	93:7
	II	5.35 (2.3)	5.45 (5.3)	93:7
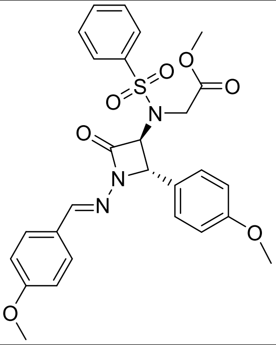	I	5.27 (2.3)	5.85 (5.0)	>95:5
	II	5.4 (2.0)	-	
	I	5.43 (2.3)	6.12 (3.5)	>95:5

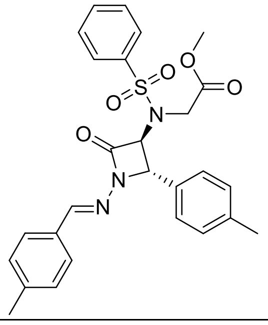	II	5.26 (2.2)	-	
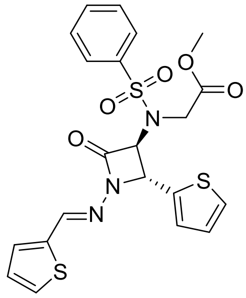	I	5.74 (2.1)	6.39 (5.3)	>95:5
	II	5.74 (2.1)	-	
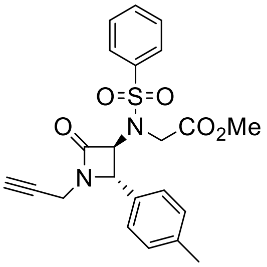	I	4.93 (2.1)	5.05 (2.6)	89:11
	II[b]	4.92 (2.1)	5.05 (2.5)	88:12
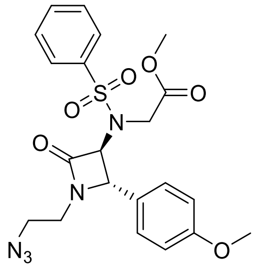	I	4.68 (5.5)	5.28 (4.7)	>95:5
	II	4.9 (2.0)	5.29 (5.1)	>95:5
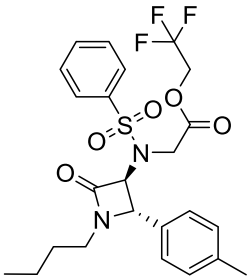	I	4.8 (1.8)	5.1 (1.7)	>95:5
	II	4.77 (2.0)	4.81 (2.0)	>95:5
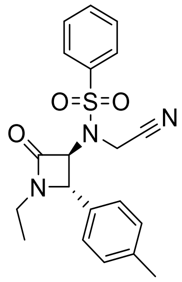	I	4.79 (2.0)	5.17 (4.7)	>95:5
	II[b]	4.79 (2.0)	5.17 (4.7)	>95:5
	I	4.53 (2.4)	5.20 (4.0)	>95:5

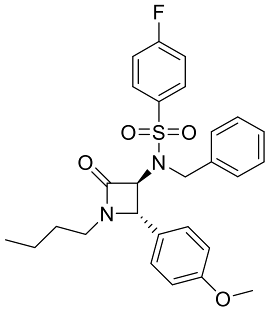	II	4.53 (2.4)	5.20 (5.0)	>95:5
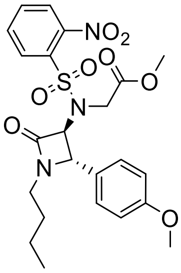	I	4.90 (1.8)	5.45 (4.3)	>95:5
	II	4.90 (1.9)	5.45 (4.7)	>95:5
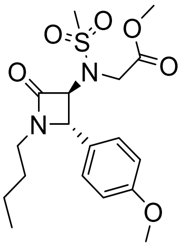	I	4.76 (2.1)	5.11 (4.9)	94:6
	II	4.75 (2.1)	5.1 (4.7)	94:6
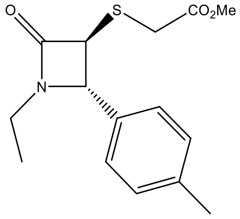	I	4.5 (2.0)	4.9 (5.3)	>95:5
	II	4.5 (2.1)	4.9 (5.0)	>95:5
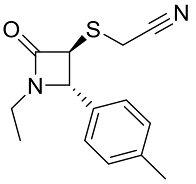	I	4.63 (2.1)	5.18 (2.9)	>95:5
	II	4.65 (2.0)	-	
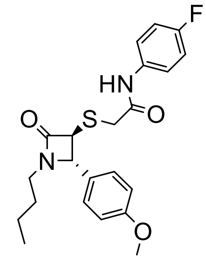	I	4.37 (2.1)	4.92 (5.3)	>95:5
	II	4.37 (2.2)	4.92 (5.1)	>95:5
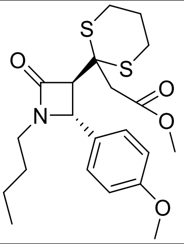	I	4.86 (2.4)	-	>95:5
	II	4.87 (2.3)	-	>95:5

[a] – crude reaction mixture, [b] – after purification

Crystallographic data

X-ray single crystal analyses were performed on Rigaku "XtaLAB Synergy-S" or "SuperNova" diffractometer with monochromated Cu Kα radiation. The temperature was kept at 100 K during data collection. Using Olex[6], the structure was solved with the SHELXT[7] structure solution program using Direct Methods and refined with the SHELXL[7] refinement package using Least Squares minimisation. *CCDC 2154010* and *2154011* contain the supplementary crystallographic data for this paper. These data can be obtained free of charge from The Cambridge Crystallographic Data Centre via http://www.ccdc.cam.ac.uk.

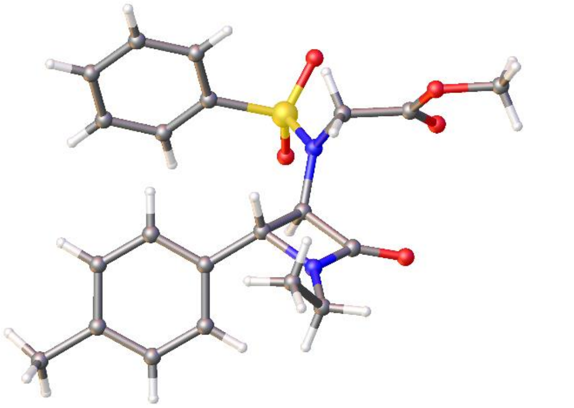

Figure S1. ORTEP representation of compound **6a** drawn at 50% probability level

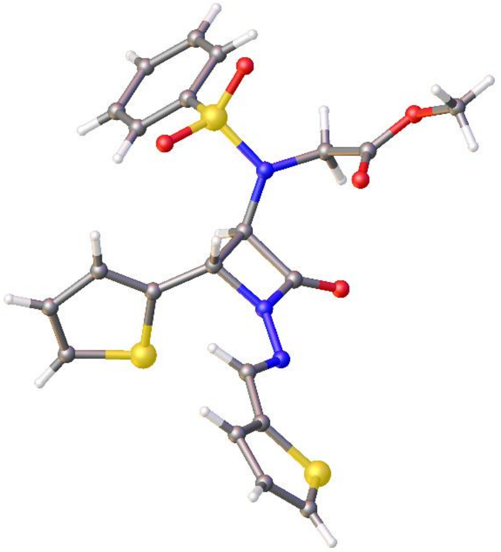

Figure S2. ORTEP representation of compound **6m** drawn at 50% probability level

Table S2. Crystal data and structure refinement for compounds **6a** and **6m**

**Identification code**	**6a**	**6m**
Empirical formula	$C_{21}H_{24}N_2O_5S$	$C_{21}H_{19}N_3O_5S_3$
Formula weight	416.48	489.57
Temperature/K	100.15	100.15
**Crystal system**	orthorhombic	monoclinic
**Space group**	Pbca	P21/n
**a/Å**	19.6670(3)	10.8532(2)
**b/Å**	9.8155(2)	10.5032(2)
**c/Å**	21.0366(4)	20.2793(4)
**α/°**	90	90
**β/°**	90	102.493(2)
**γ/°**	90	90
**Volume/Å^3**	4060.94(13)	2256.97(8)
**Z**	8	4
**ρ_{calc}g/cm^3**	1.362	1.441
**μ/mm^{-1}**	1.722	3.341

**F(000)**	1760.0	1016.0
**Crystal size/mm^3**	0.08 × 0.06 × 0.04	0.08 × 0.06 × 0.04
**Radiation**	CuKα (λ = 1.54184)	CuKα (λ = 1.54184)
**2Θ range for data collection/°**	8.406 to 159.986	8.57 to 152.43
**Index ranges**	-16 ≤ h ≤ 24, -12 ≤ k ≤ 11, -25 ≤ l ≤ 26	-13 ≤ h ≤ 13, -13 ≤ k ≤ 12, -25 ≤ l ≤ 15
**Reflections collected**	16399	10125
**Independent reflections**	4293 [R_{int} = 0.0415, R_{sigma} = 0.0379]	4628 [Rint = 0.0312, Rsigma = 0.0291]
**Data/restraints/parameters**	4293/0/265	4628/0/290
**Goodness-of-fit on F^2**	1.062	1.037
**Final R indexes [I>=2σ (I)]**	R_1 = 0.0443, wR_2 = 0.1141	R1 = 0.0384, wR2 = 0.1009
**Final R indexes [all data]**	R_1 = 0.0506, wR_2 = 0.1178	R1 = 0.0406, wR2 = 0.1032
**Largest diff. peak/hole / e Å^{-3}**	0.32/-0.65	0.50/-0.50
**CCDC**	*2154010*	*2154011*

References

1. Murali Dhar, T. G.; Shen, Z.; Gu, H. H.; Chen, P.; Norris, D.; Watterson, S. H.; Ballentine, S. K.; Fleener, C. A.; Rouleau, K. A.; Barrish, J. C.; Townsend, R.; Hollenbaugh, D. L.; Iwanowicz, E. J., 3-Cyanoindole-based inhibitors of inosine monophosphate dehydrogenase: synthesis and initial structure–Activity relationships. *Bioorganic & Medicinal Chemistry Letters* **2003,** *13* (20), 3557-3560.
2. C.P., D. Morpholine and thiomorpholine tachykinin receptor antagonists 1999-02-16, 1999.
3. Cherney, R. J.; Duan, J. J.; Voss, M. E.; Chen, L.; Wang, L.; Meyer, D. T.; Wasserman, Z. R.; Hardman, K. D.; Liu, R. Q.; Covington, M. B.; Qian, M.; Mandlekar, S.; Christ, D. D.; Trzaskos, J. M.; Newton, R. C.; Magolda, R. L.; Wexler, R. R.; Decicco, C. P., Design, synthesis, and evaluation of benzothiadiazepine hydroxamates as selective tumor necrosis factor-alpha converting enzyme inhibitors. *J Med Chem* **2003,** *46* (10), 1811-23.
4. Usmanova, L.; Bakulina, O.; Dar'in, D.; Krasavin, M., Spontaneous formation of tricyclic lactones following the Castagnoli–Cushman reaction. *Chemistry of Heterocyclic Compounds* **2017,** *53* (4), 474-479.
5. DeMarinis, R. M.; Boehm, J. C.; Dunn, G. L.; Hoover, J. R.; Uri, J. V.; Guarini, J. R.; Phillips, L.; Actor, P.; Weisbach, J. A., Semisynthetic cephalosporins. Synthesis and structure-activity relationships of analogues with 7-acyl groups derived from 2-(cyanomethylthio)acetic acid or 2-[(2,2,2-trifluoroethyl)thio]acetic acid and their sulfoxides and sulfones. *J Med Chem* **1977,** *20* (1), 30-5.
6. Dolomanov, O. V.; Bourhis, L. J.; Gildea, R. J.; Howard, J. A. K.; Puschmann, H., OLEX2: a complete structure solution, refinement and analysis program. *Journal of Applied Crystallography* **2009,** *42* (2), 339-341.
7. Linden, A., Chemistry and structure in Acta Crystallographica Section C. *Acta Crystallogr C Struct Chem* **2015,** *71* (Pt 1), 1-2.

Copies of ^{1}H and ^{13}C NMR spectra

^{1}H and ^{13}C NMR spectra of compound **4**

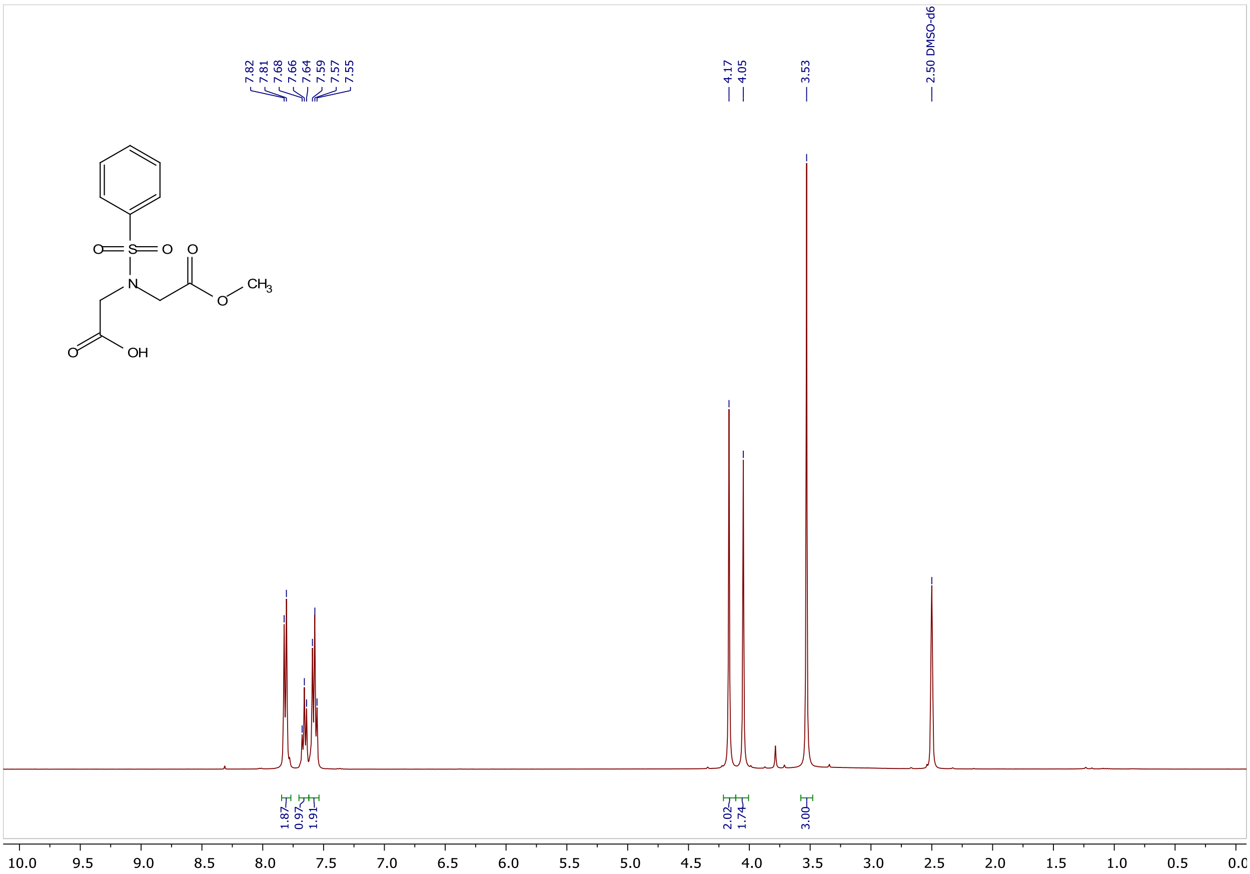

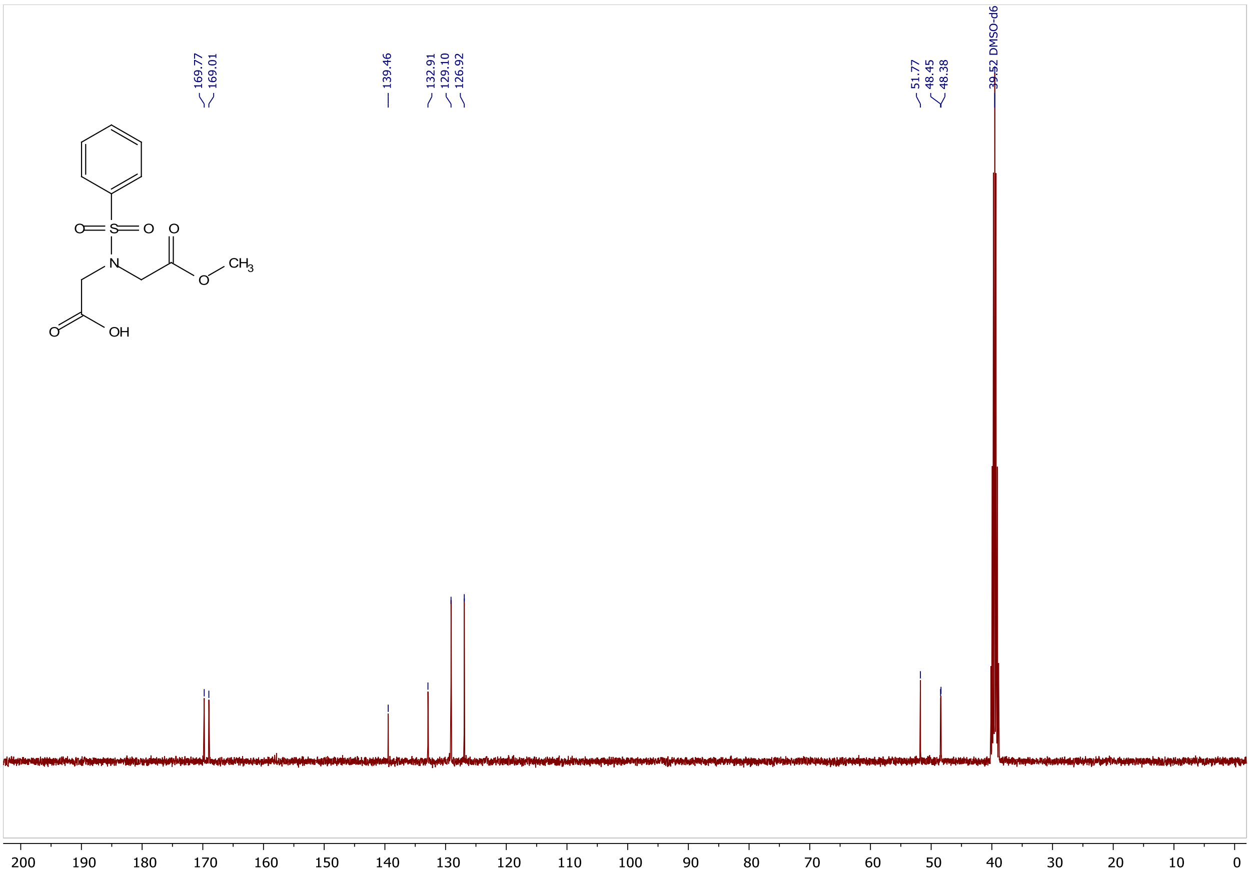

^{1}H and ^{13}C NMR spectra of compound ***11d***

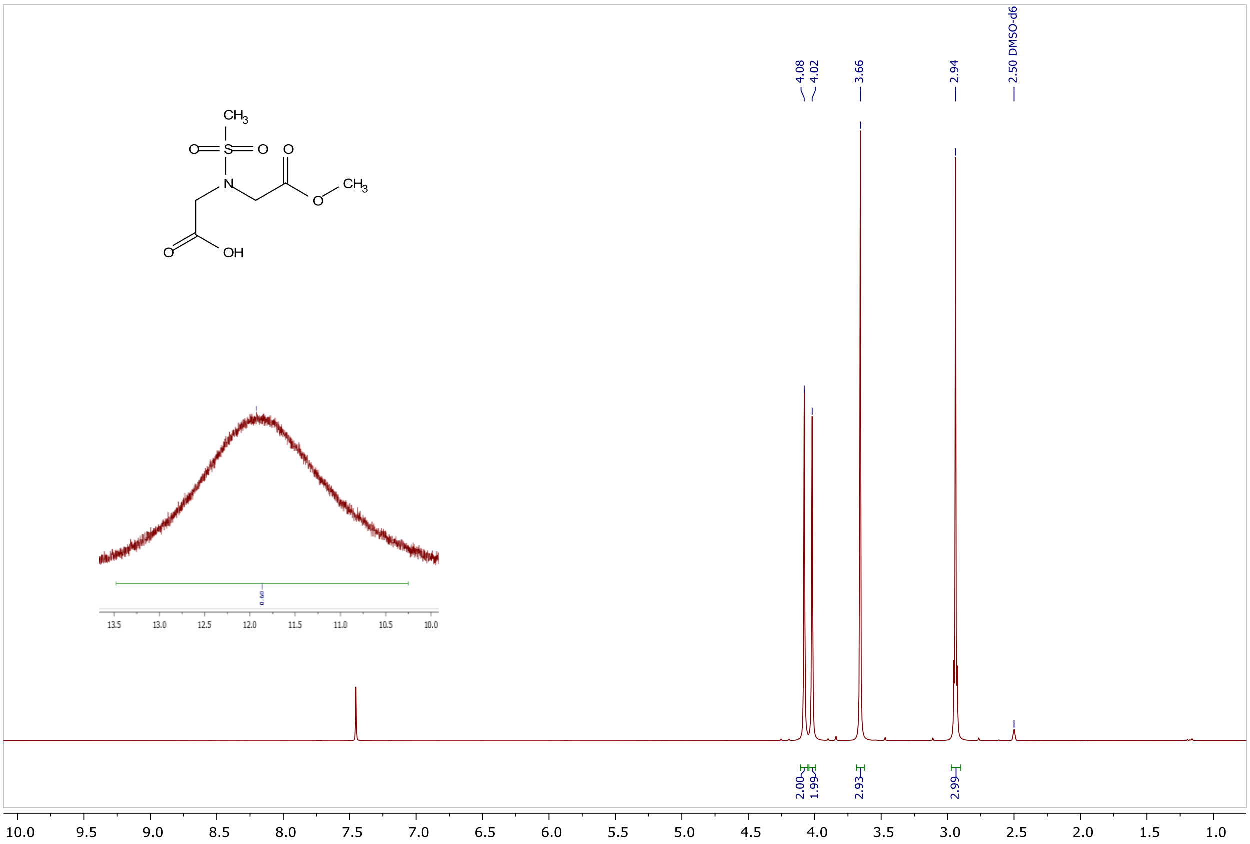

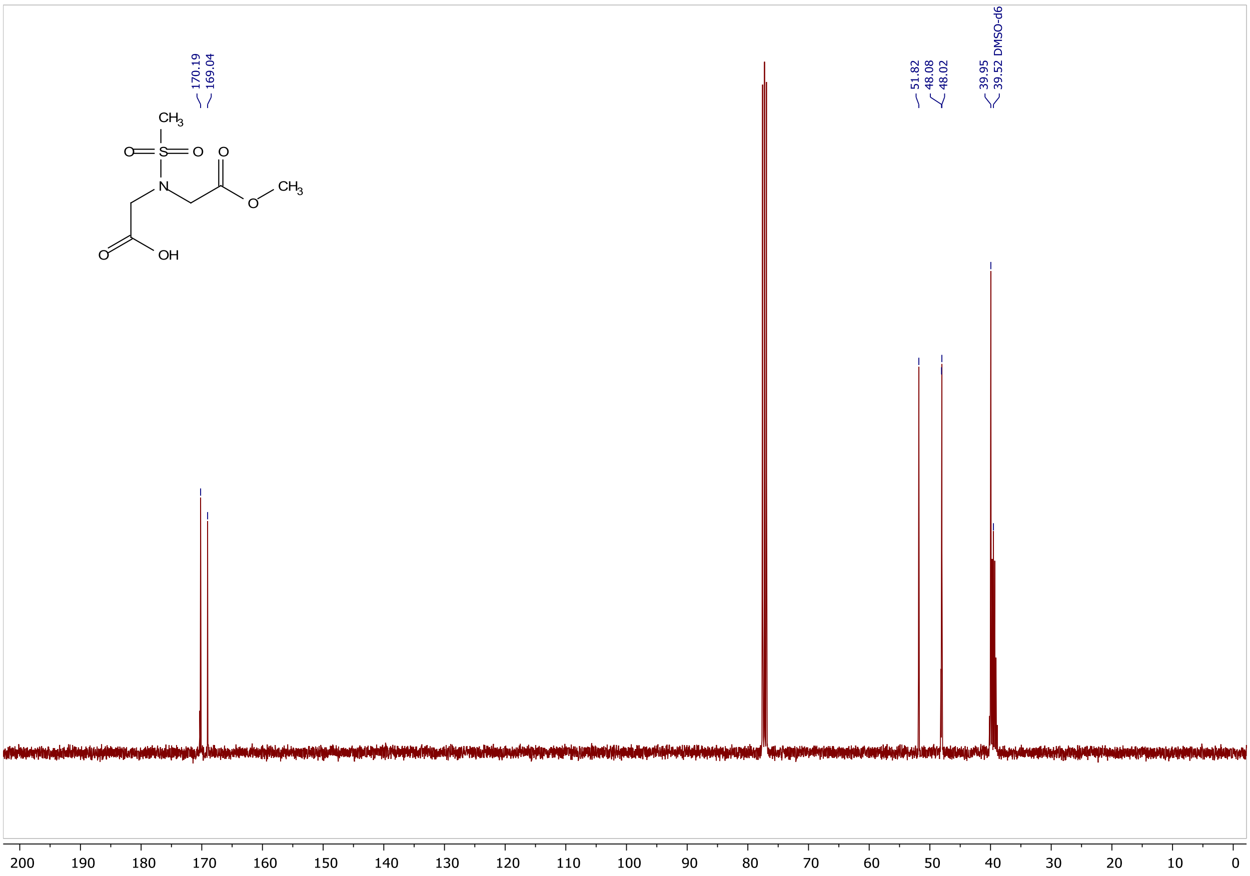

^1H and ^{13}C NMR spectra of compound ***11e***

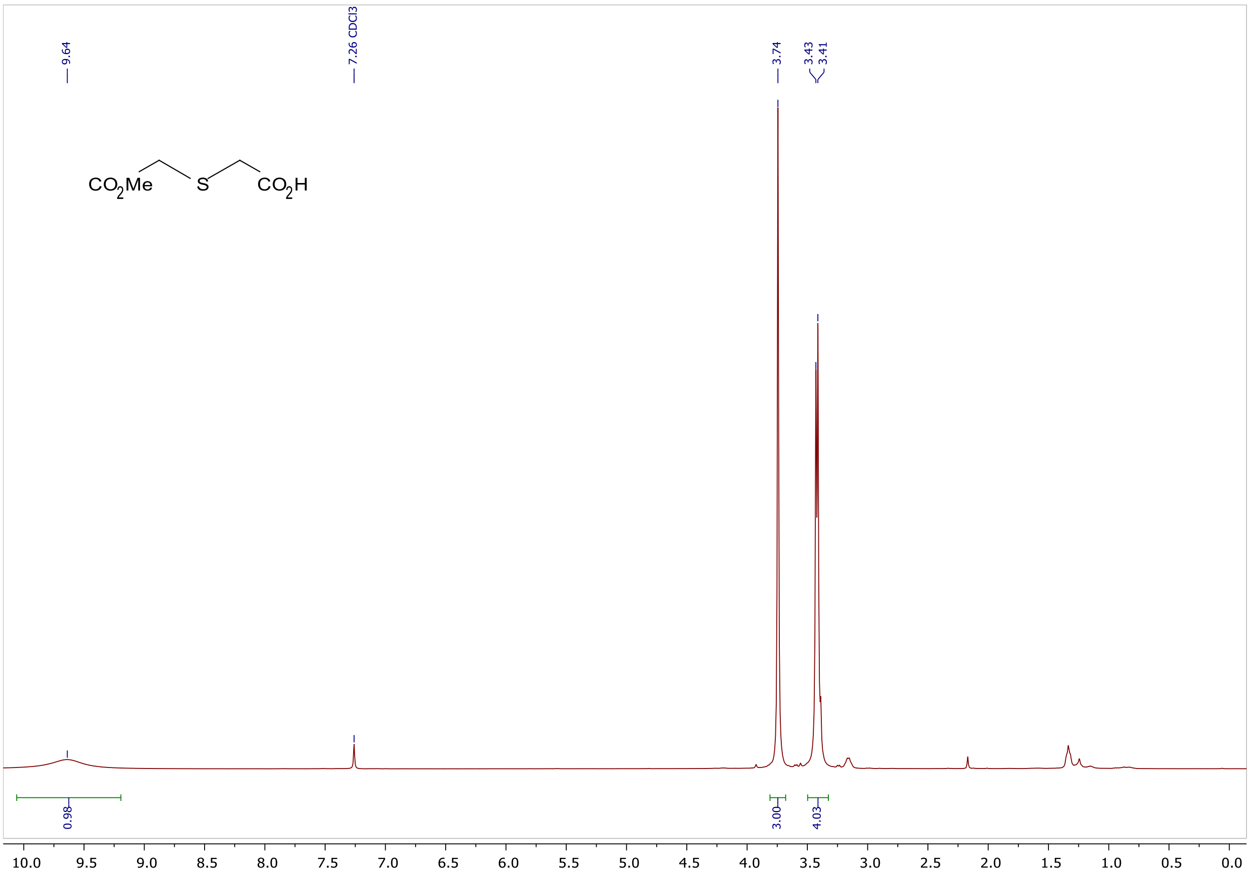

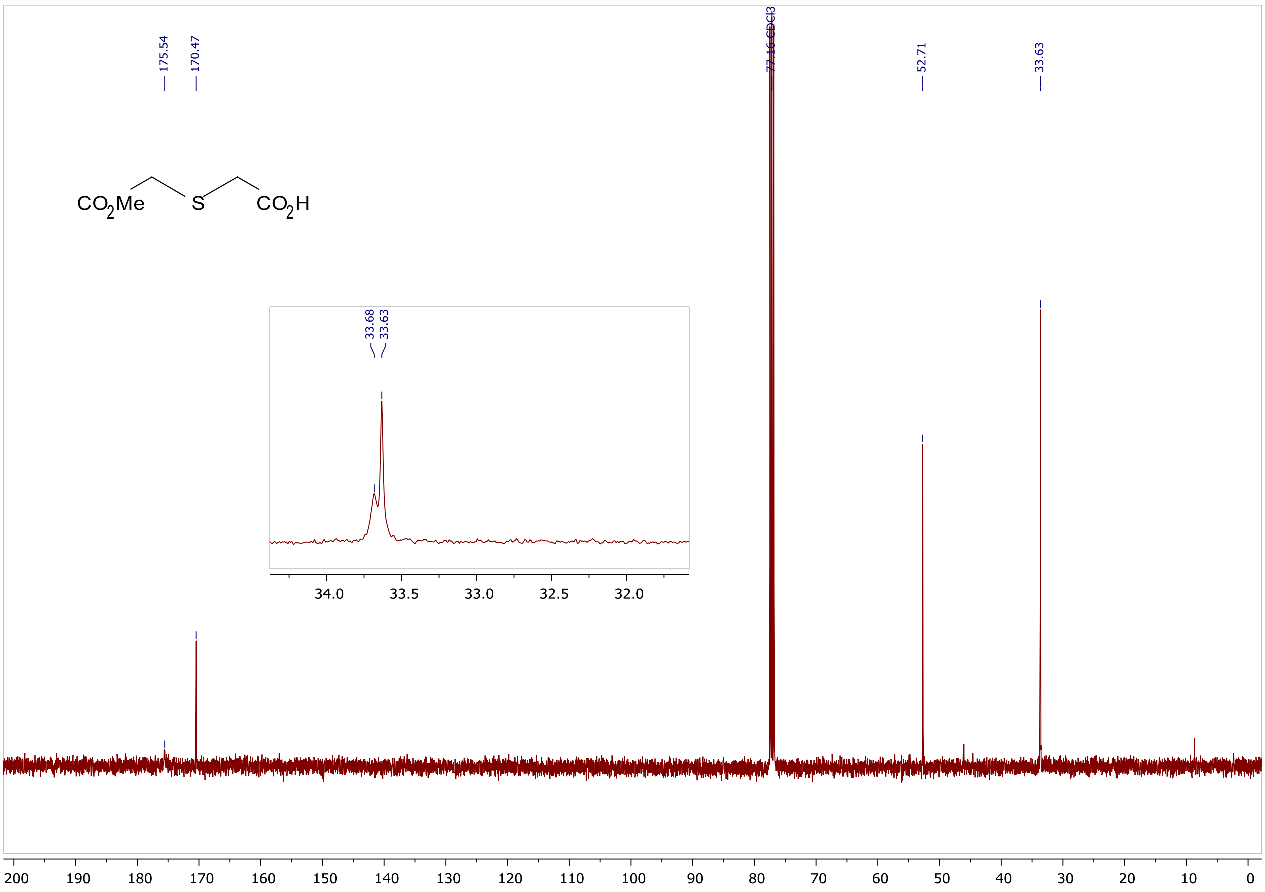

^{1}H, ^{13}C and ^{19}F NMR spectra of compound ***11g***

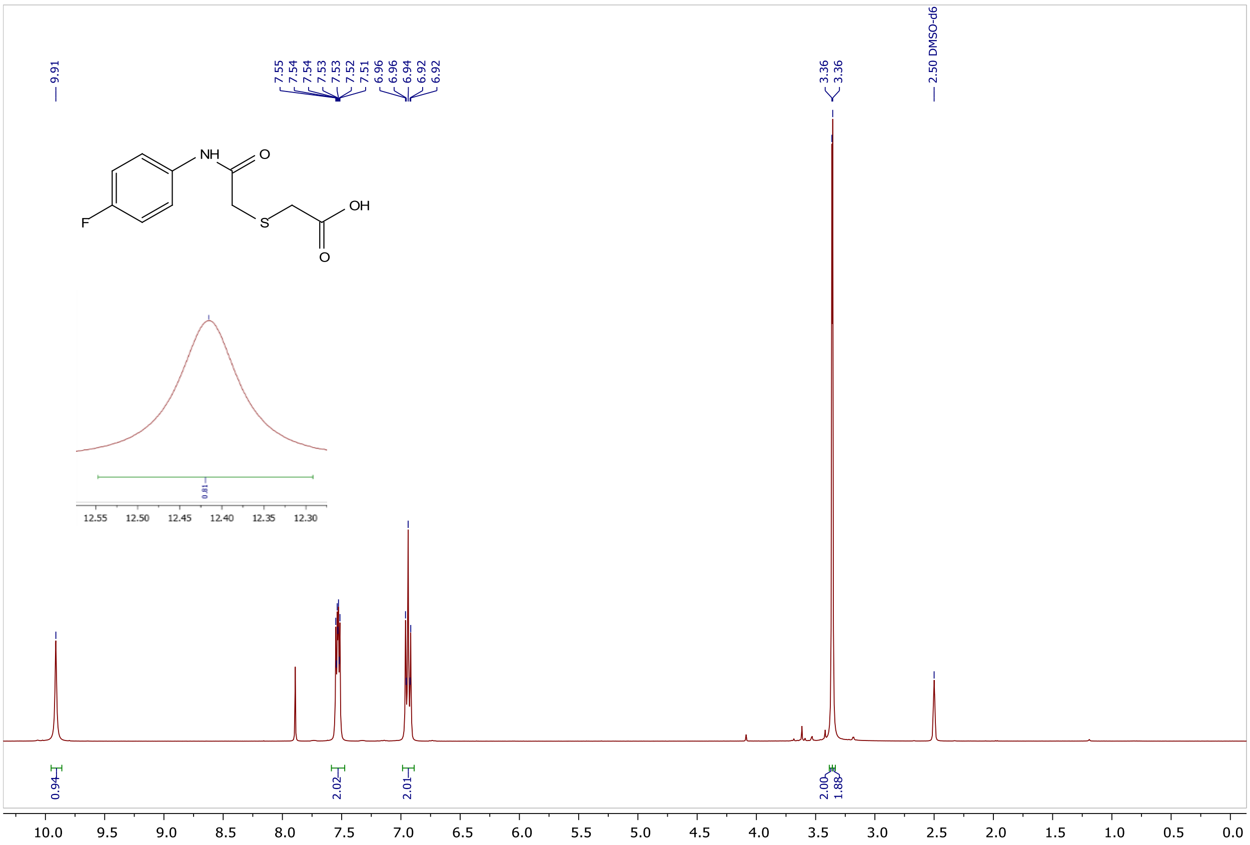

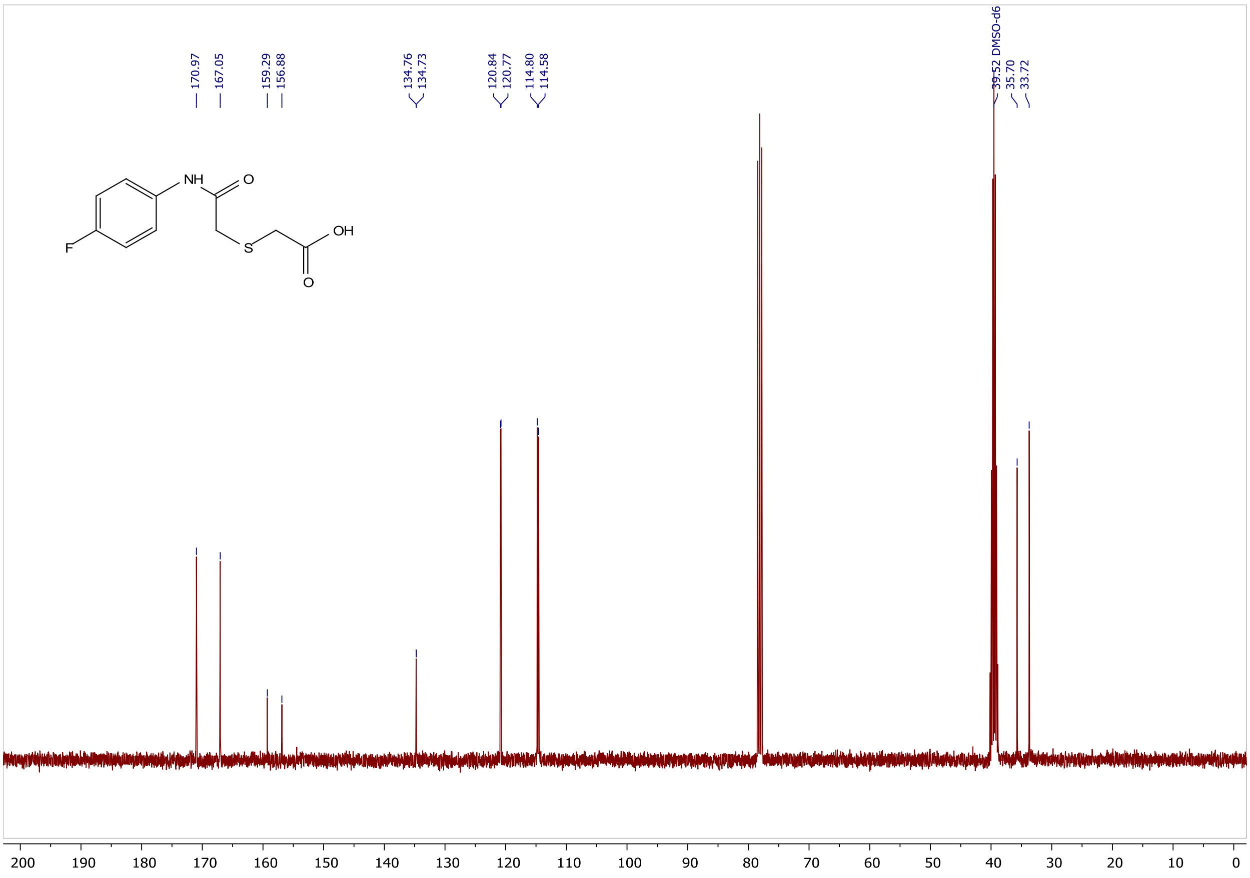

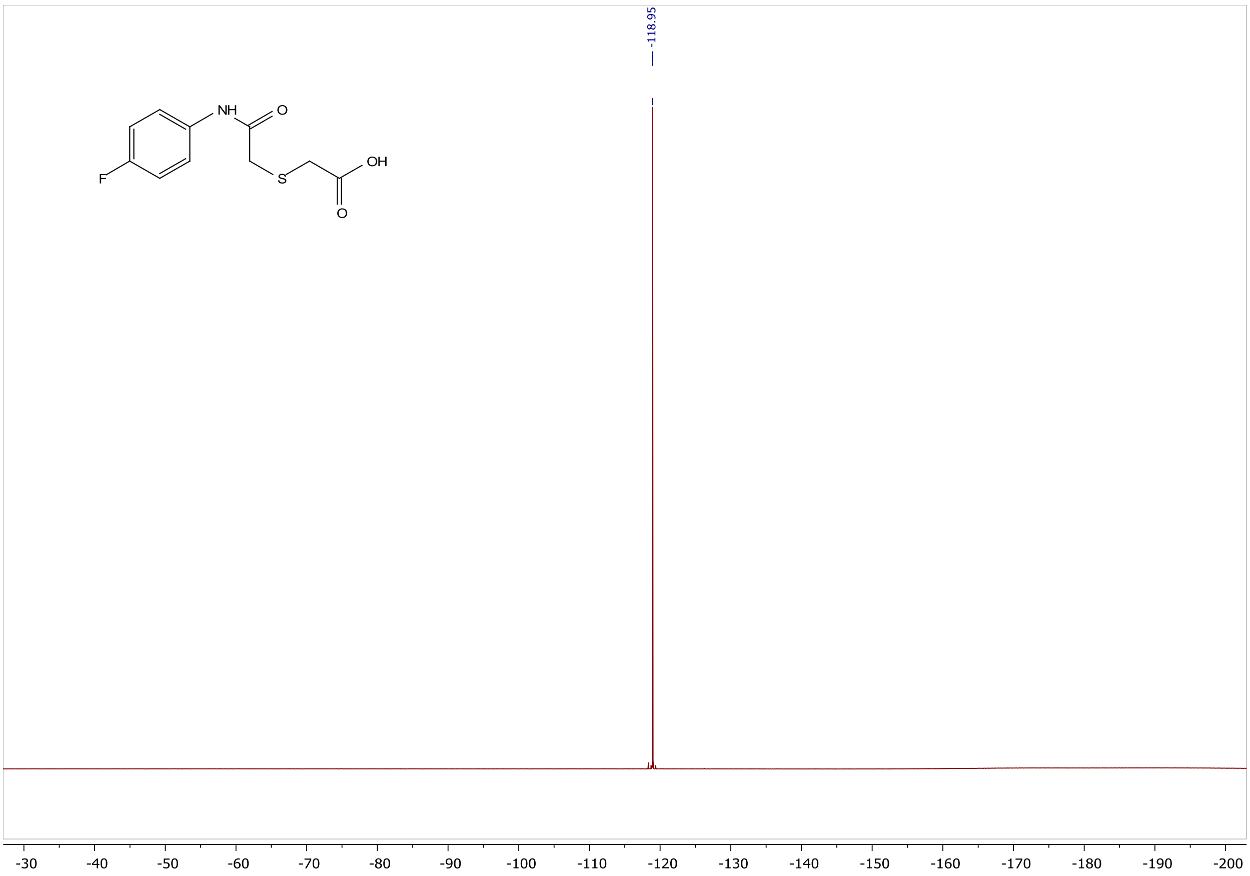

^{1}H and ^{13}C NMR spectra of compound ***11m***

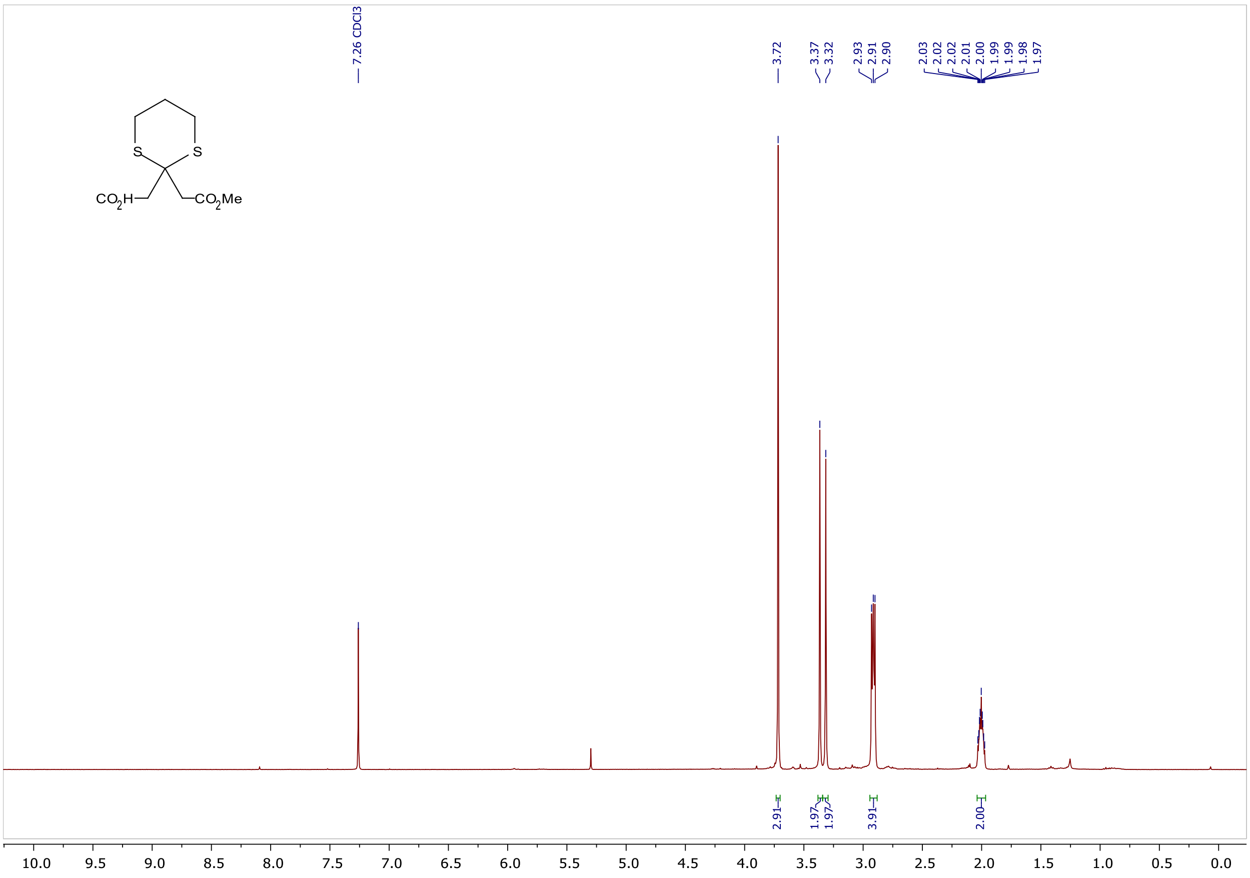

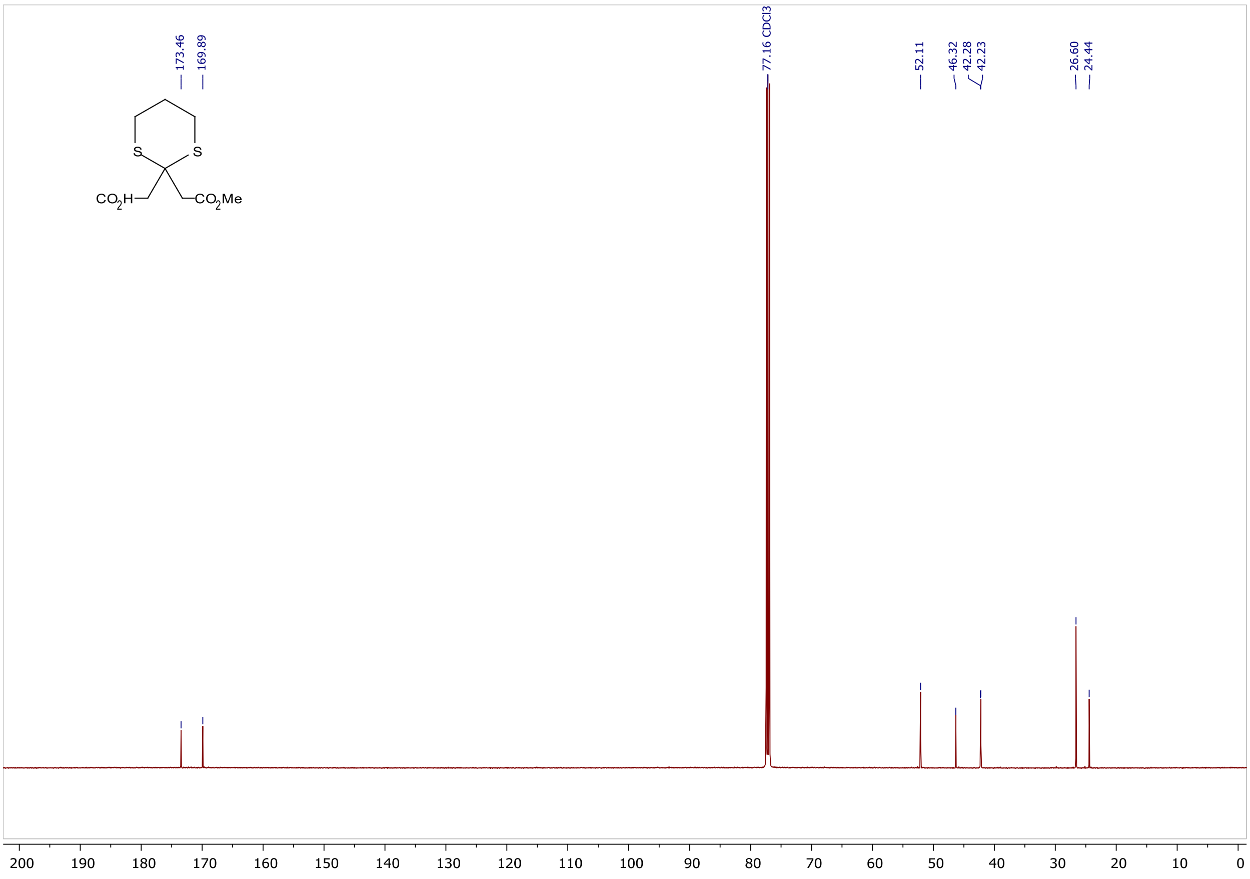

^{1}H and ^{13}C NMR spectra of compound ***S1***

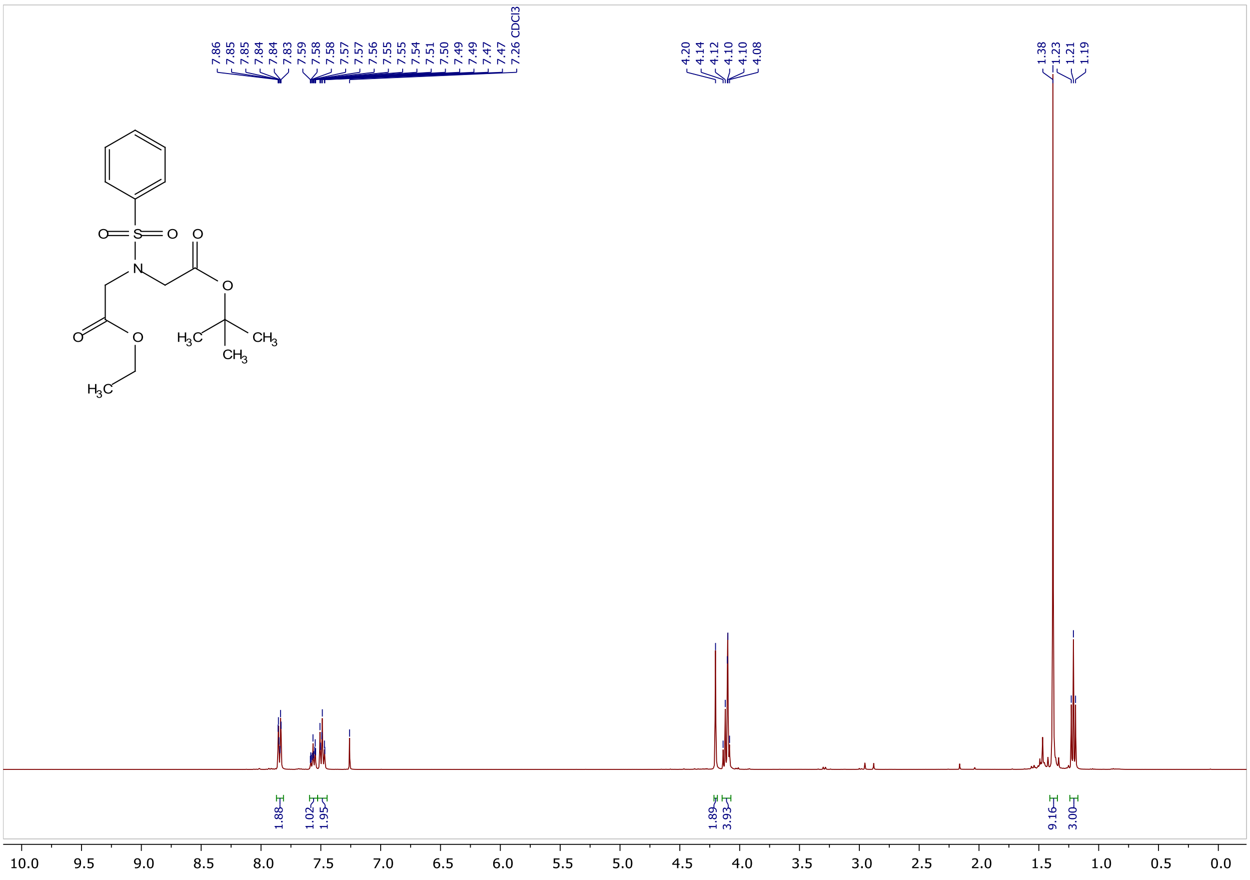

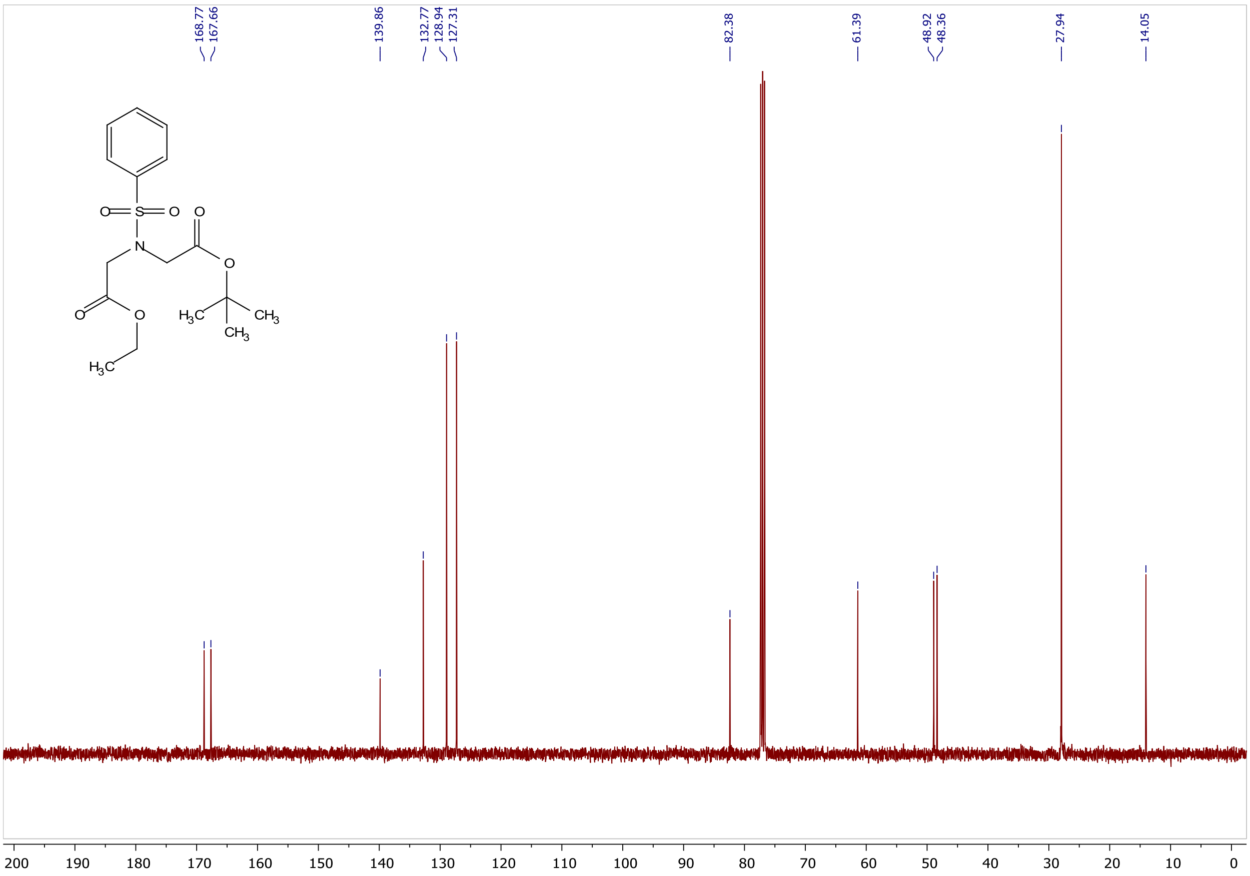

^{1}H and ^{13}C NMR spectra of compound ***S2***

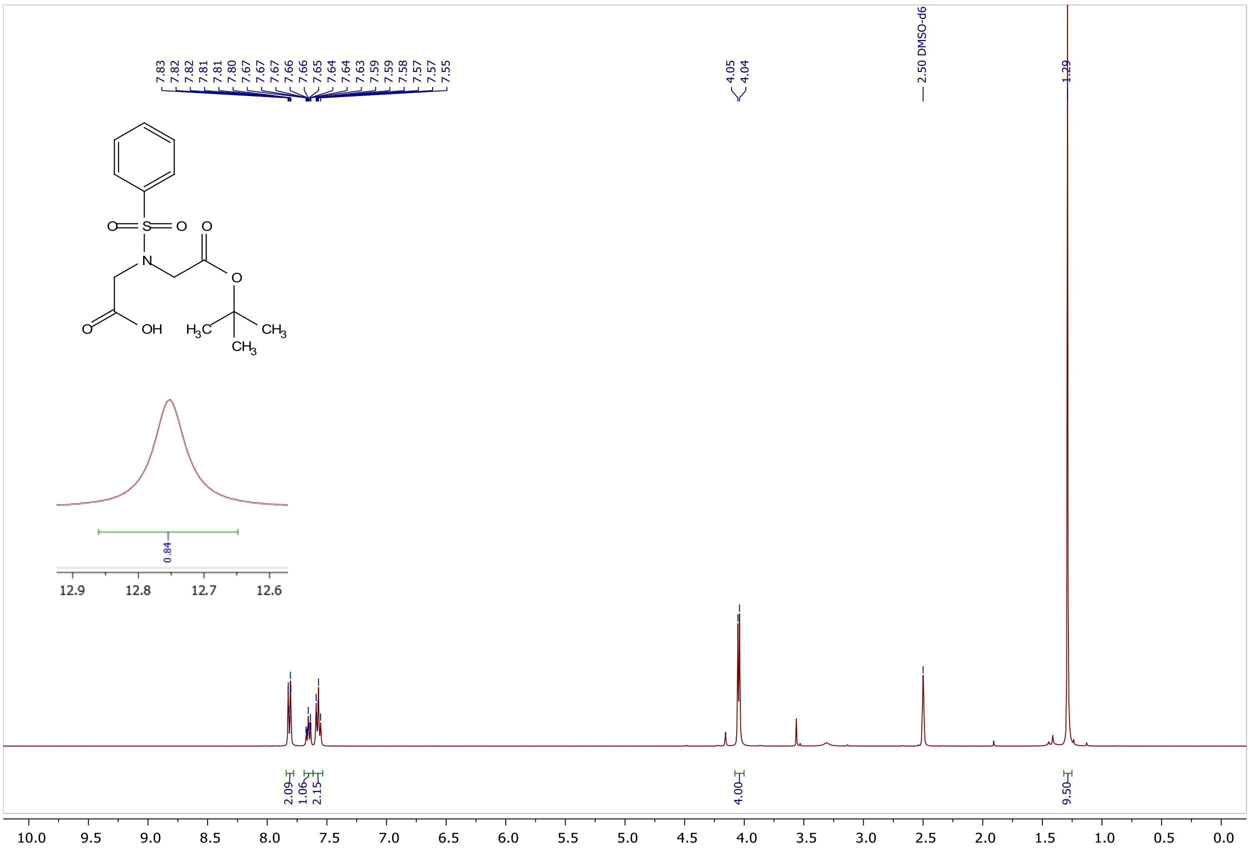

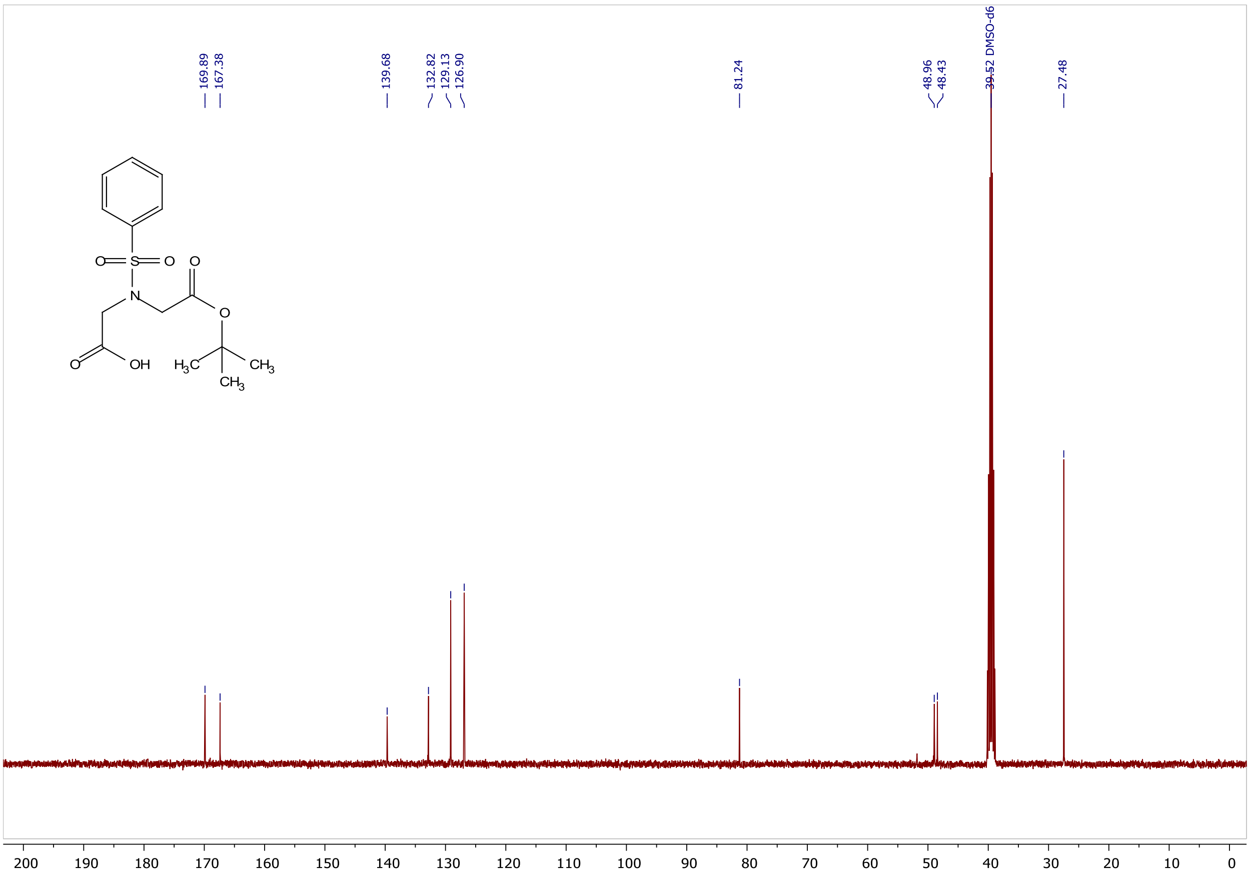

^{1}H, ^{13}C and ^{19}F NMR spectra of compound ***S3***

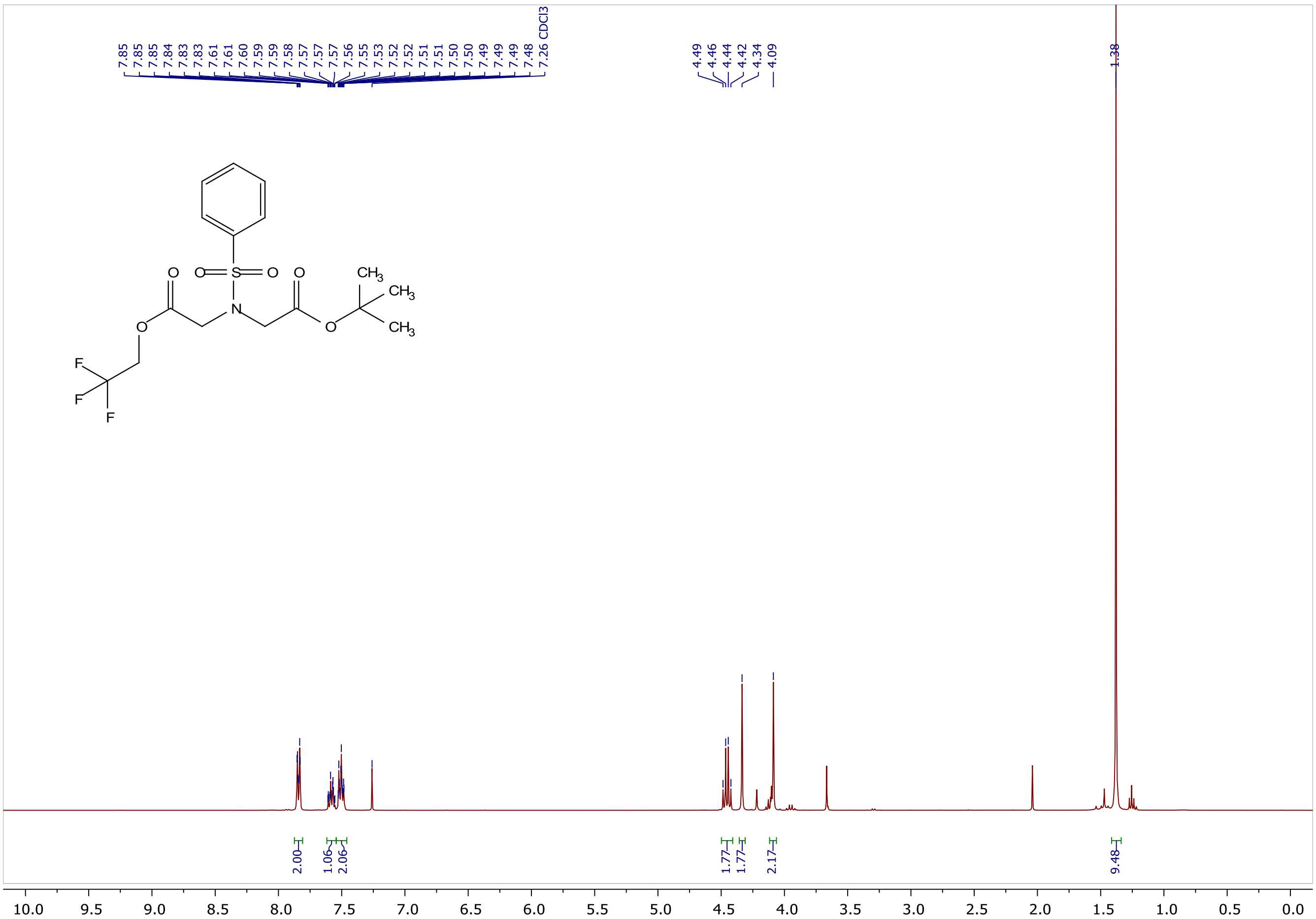

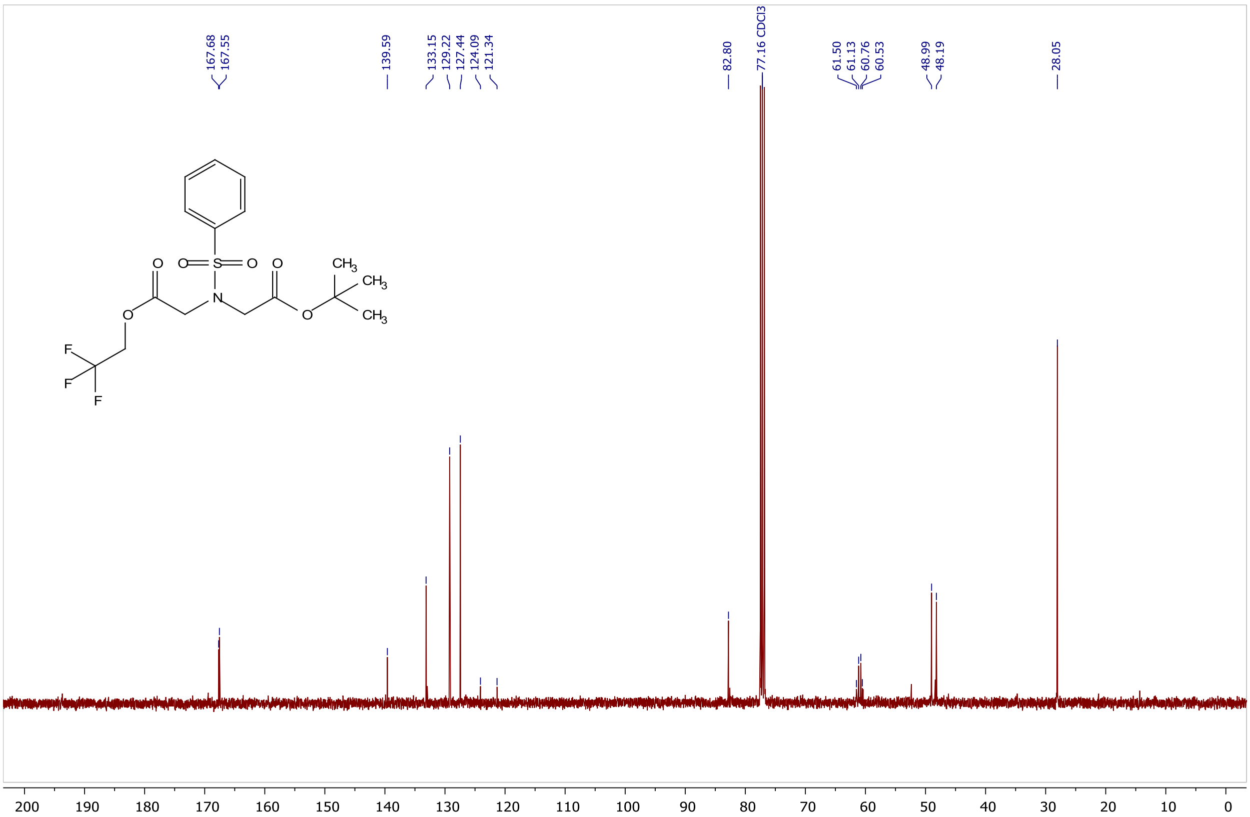
167.68
167.55
139.59
133.15
129.22
127.44
124.09
121.34
82.80
77.16 CDCl3
61.50
61.13
60.76
60.53
48.99
48.19
28.05
200 190 180 170 160 150 140 130 120 110 100 90 80 70 60 50 40 30 20 10 0

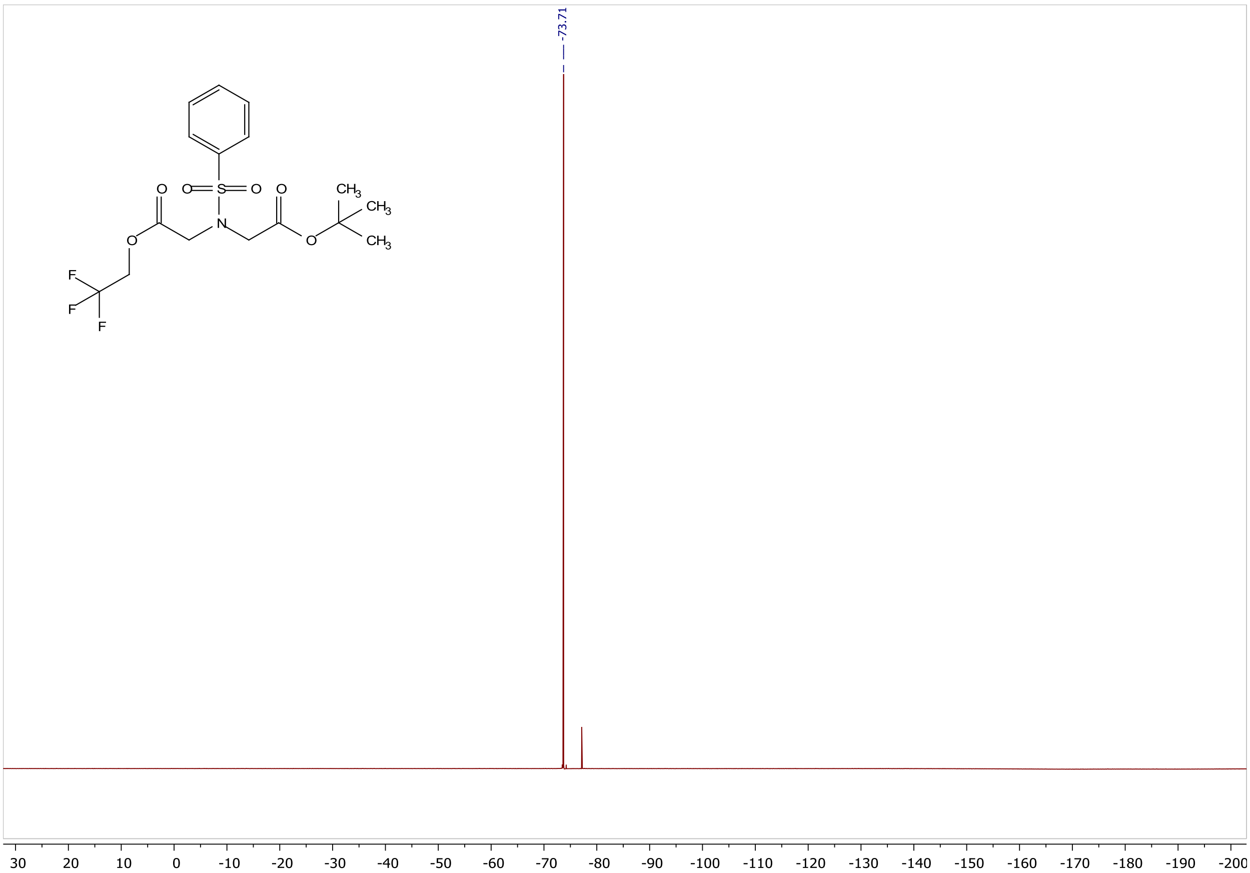
-73.71
30 20 10 0 -10 -20 -30 -40 -50 -60 -70 -80 -90 -100 -110 -120 -130 -140 -150 -160 -170 -180 -190 -200

^{1}H, ^{13}C and ^{19}F NMR spectra of compound ***S4***

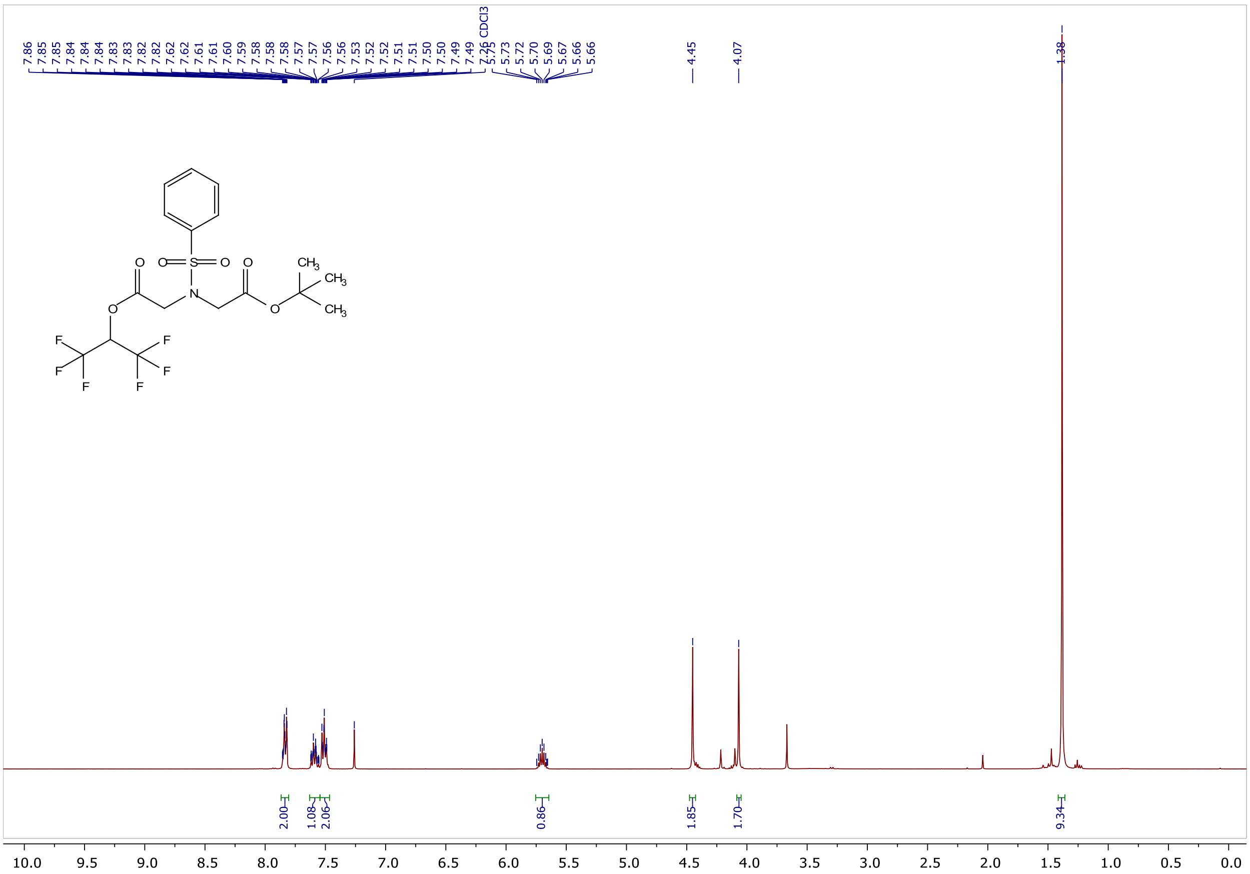

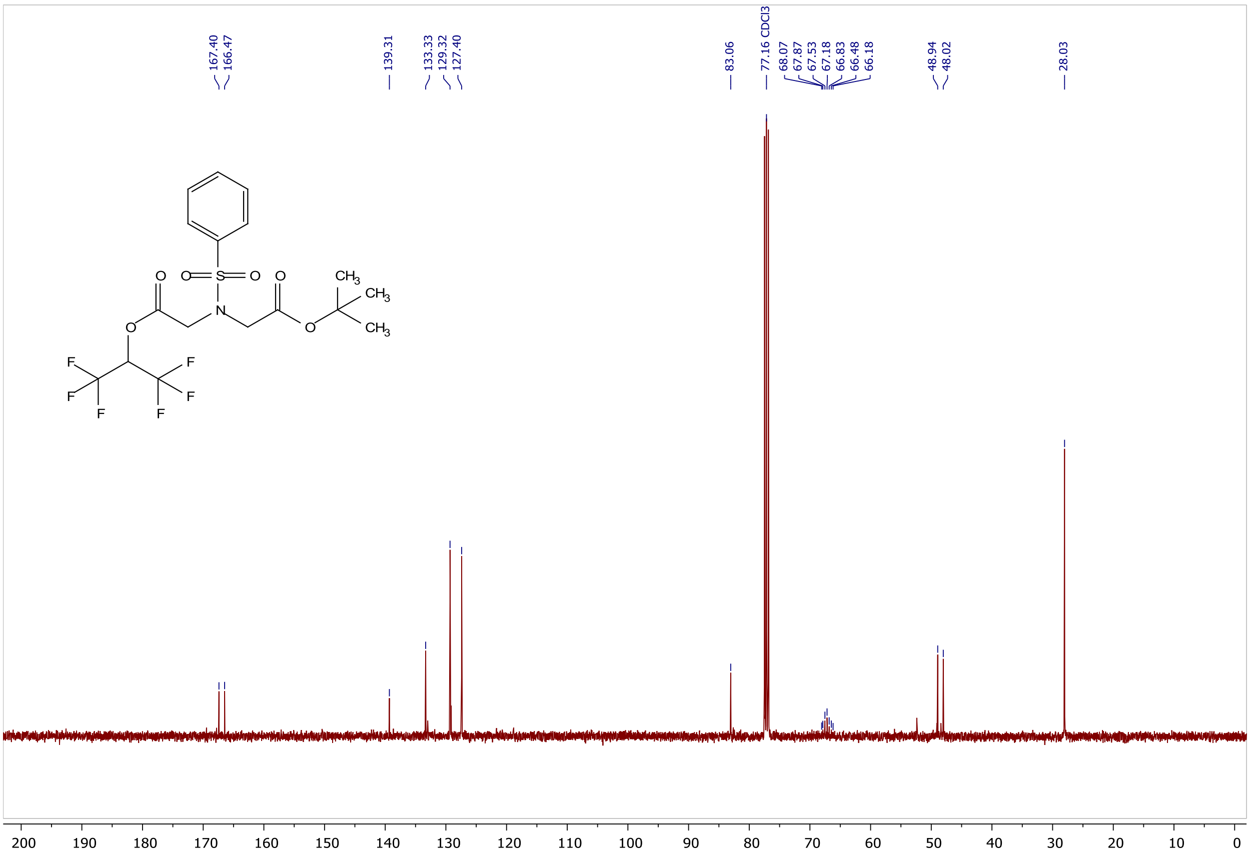

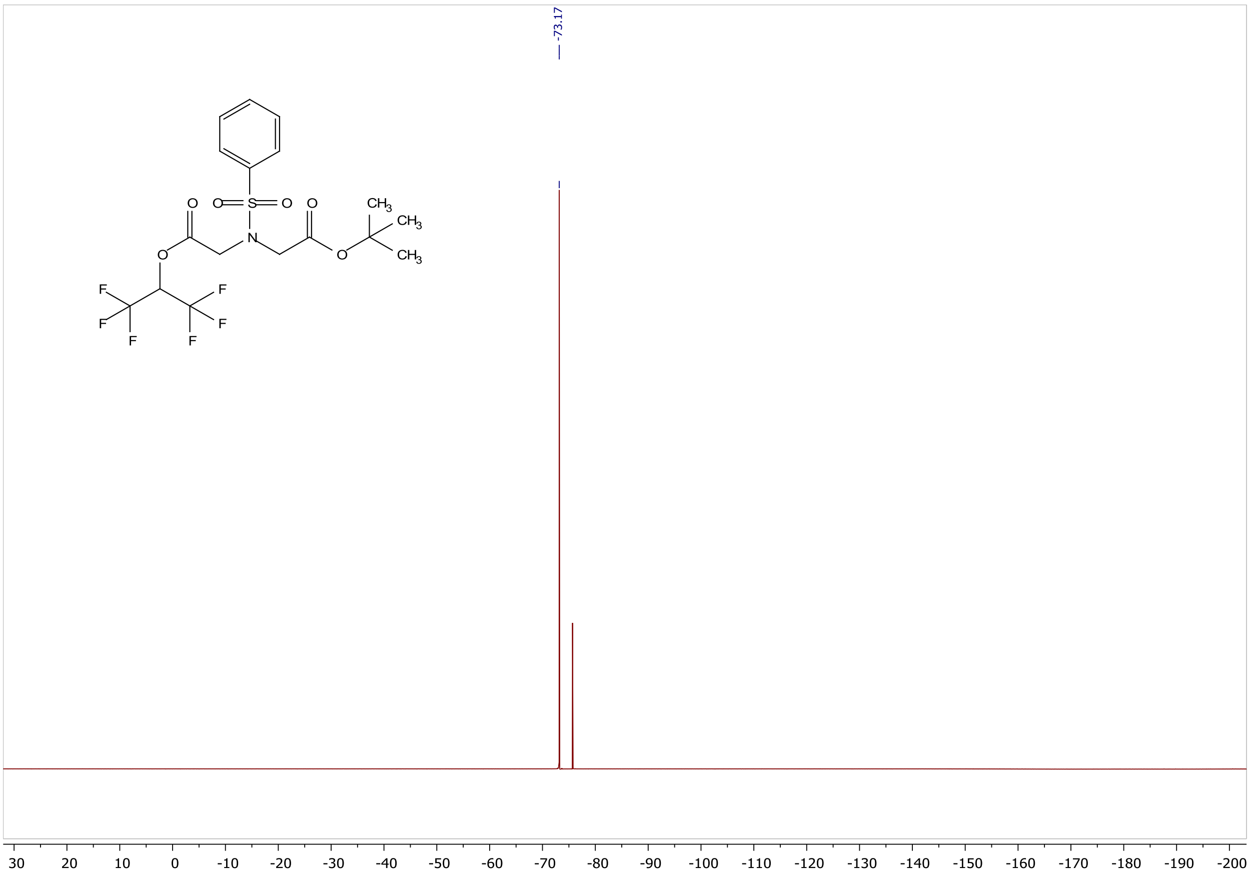

^{1}H, ^{13}C and ^{19}F NMR spectra of compound **13**

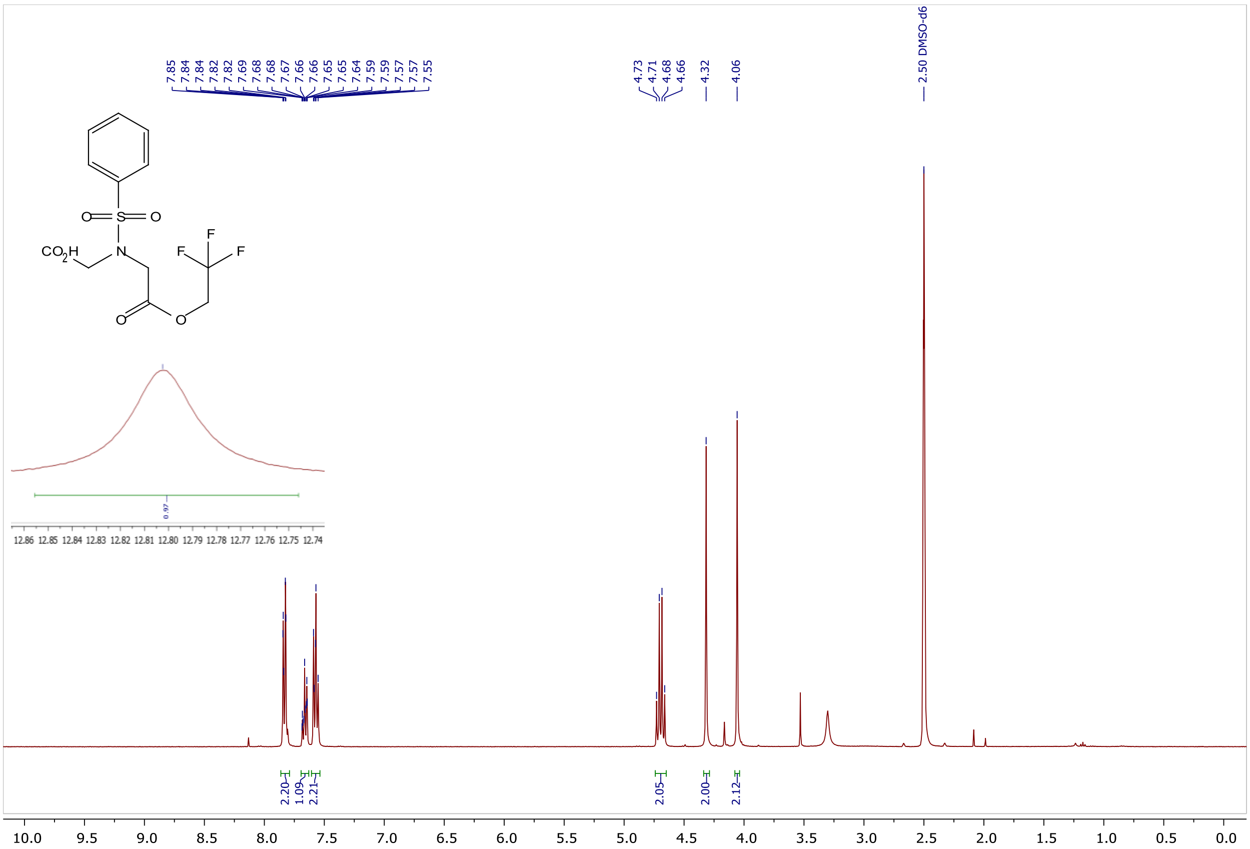

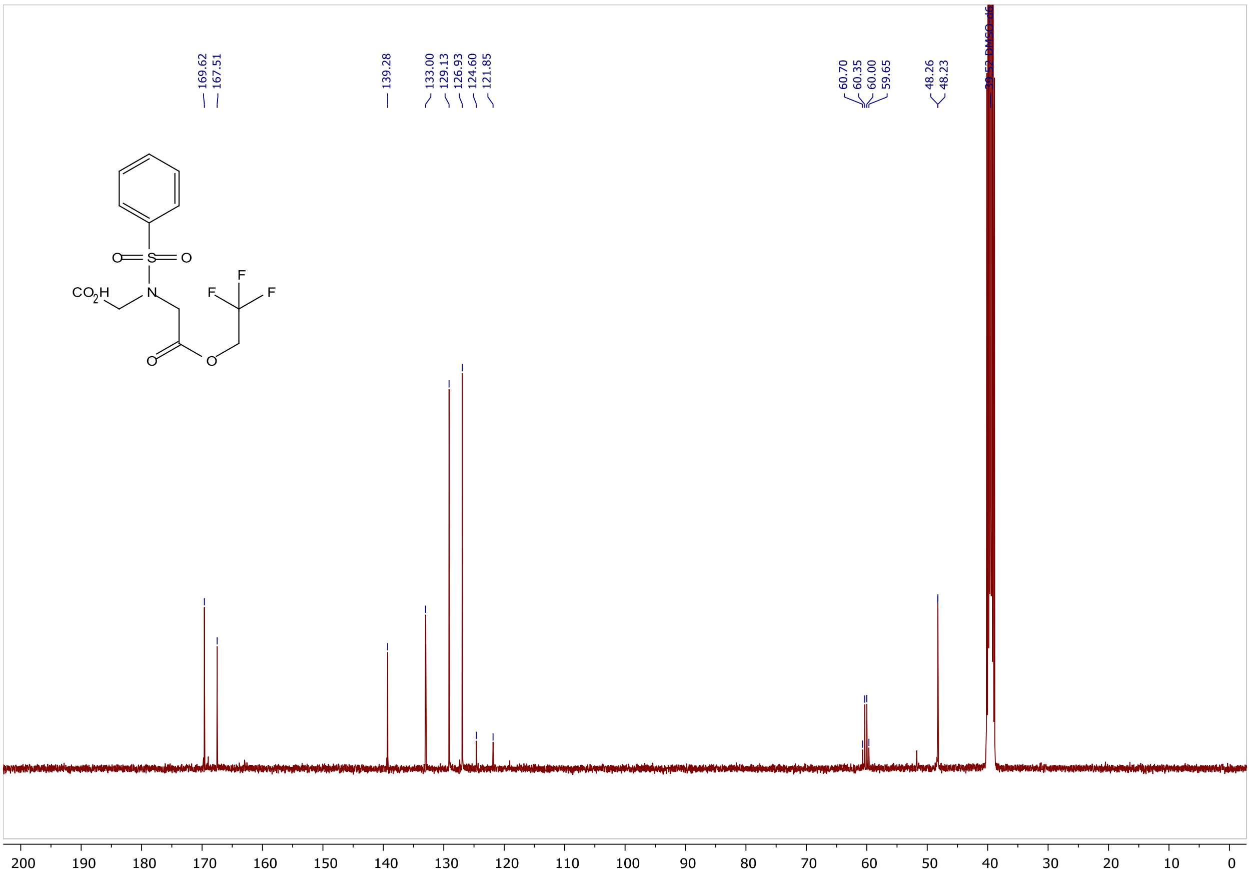
169.62
167.51
139.28
133.00
129.13
126.93
124.60
121.85
60.70
60.35
60.00
59.65
48.26
48.23
CO2H
N
O
S
O
F
F
F
O
O
200 190 180 170 160 150 140 130 120 110 100 90 80 70 60 50 40 30 20 10 0

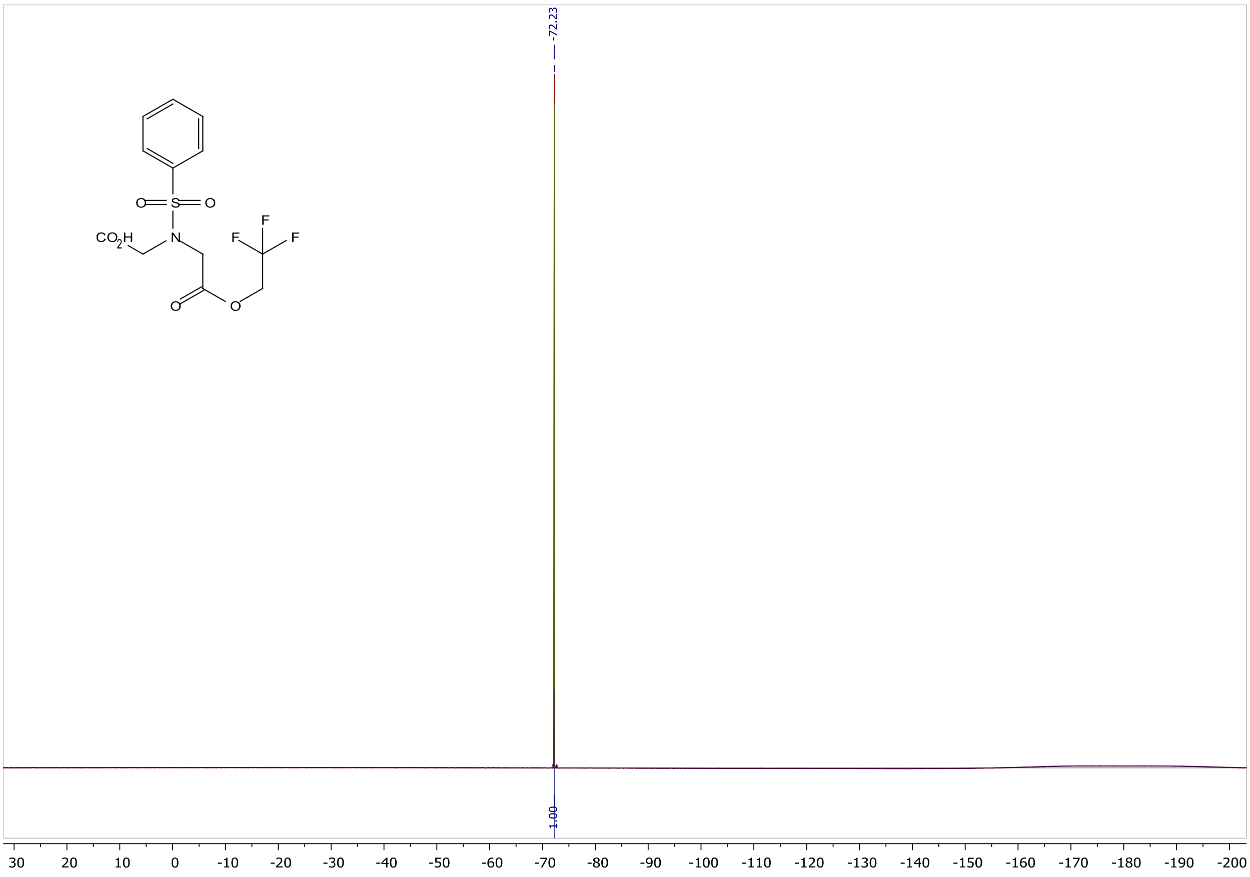
-72.23
1.00
CO2H
N
O
S
O
F
F
F
O
O
30 20 10 0 -10 -20 -30 -40 -50 -60 -70 -80 -90 -100 -110 -120 -130 -140 -150 -160 -170 -180 -190 -200

^{1}H, ^{13}C and ^{19}F NMR spectra of compound **14**

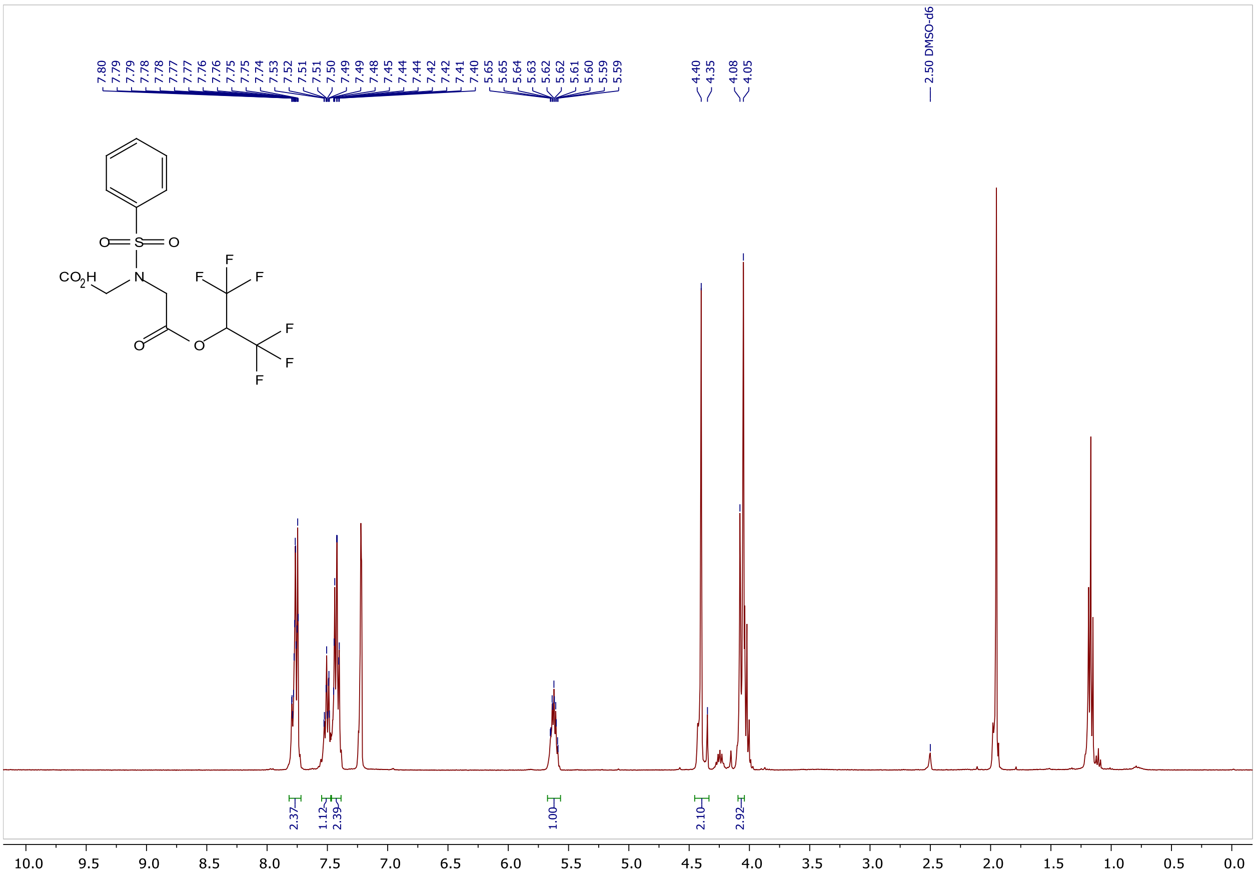

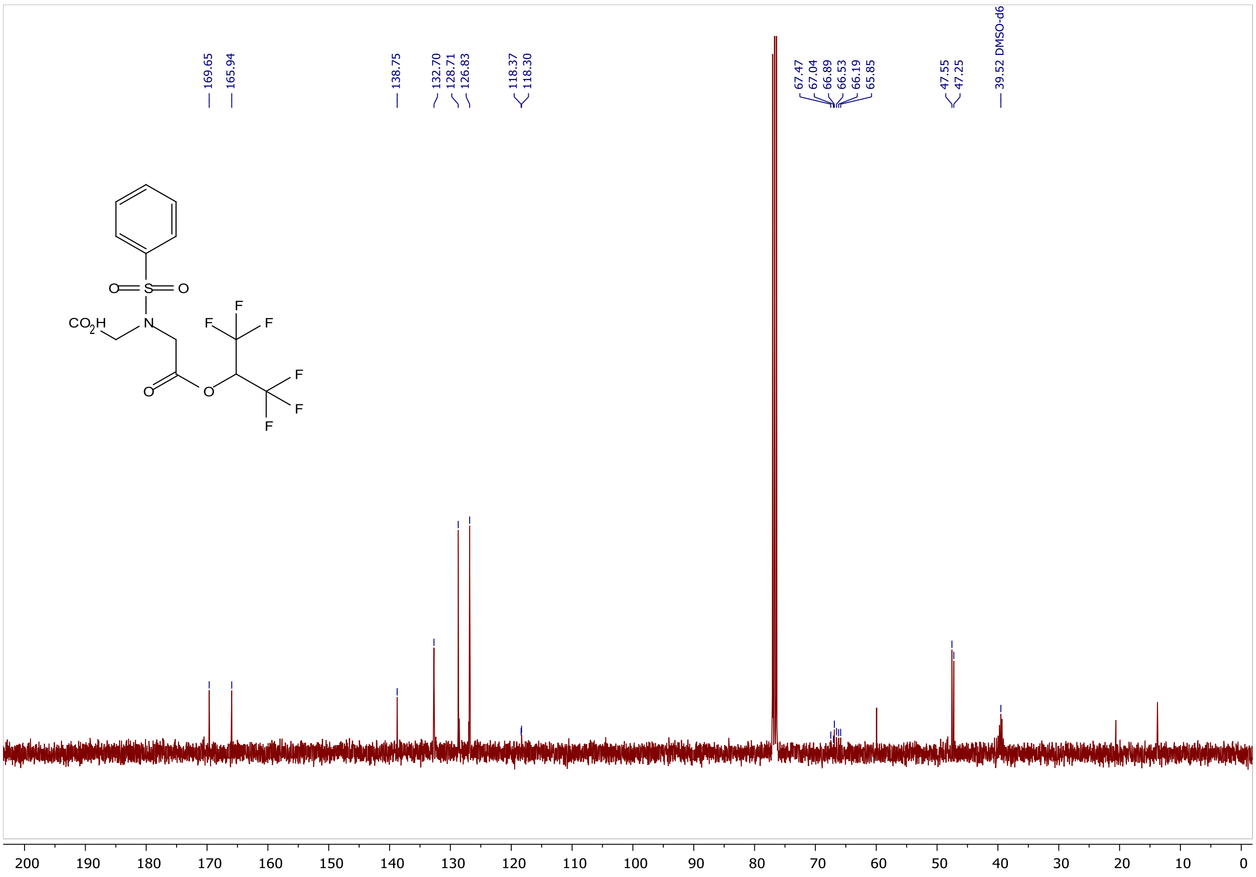

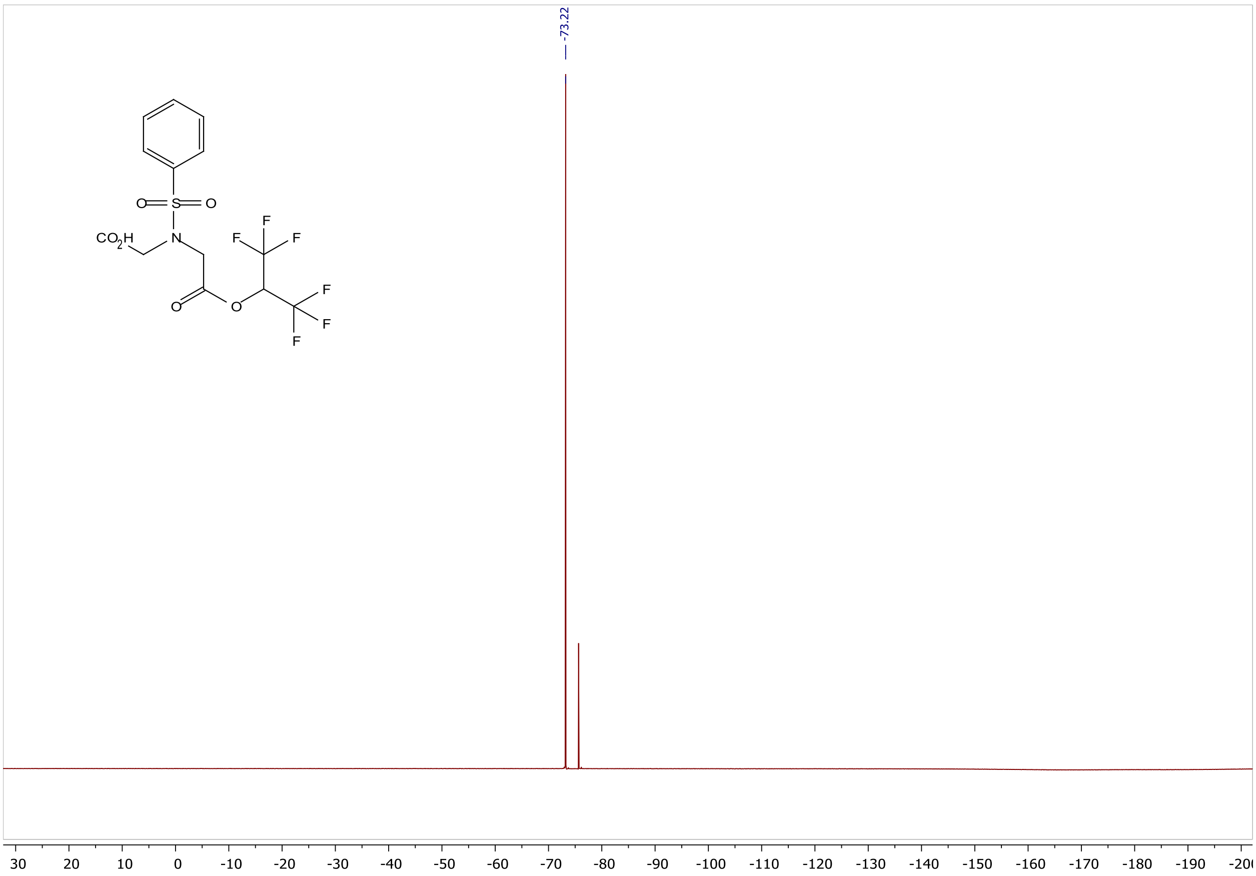

^{1}H and ^{13}C NMR spectra of compound **6a**

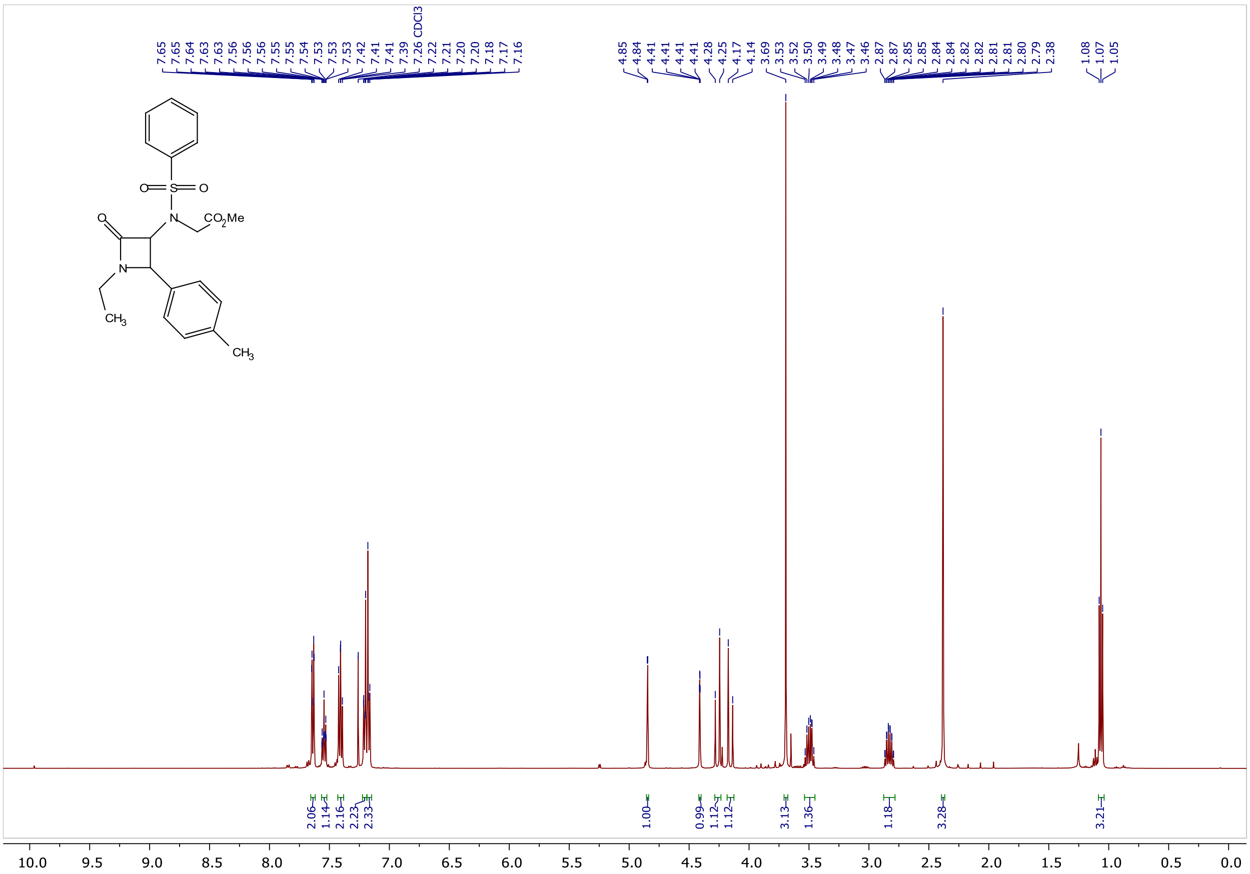

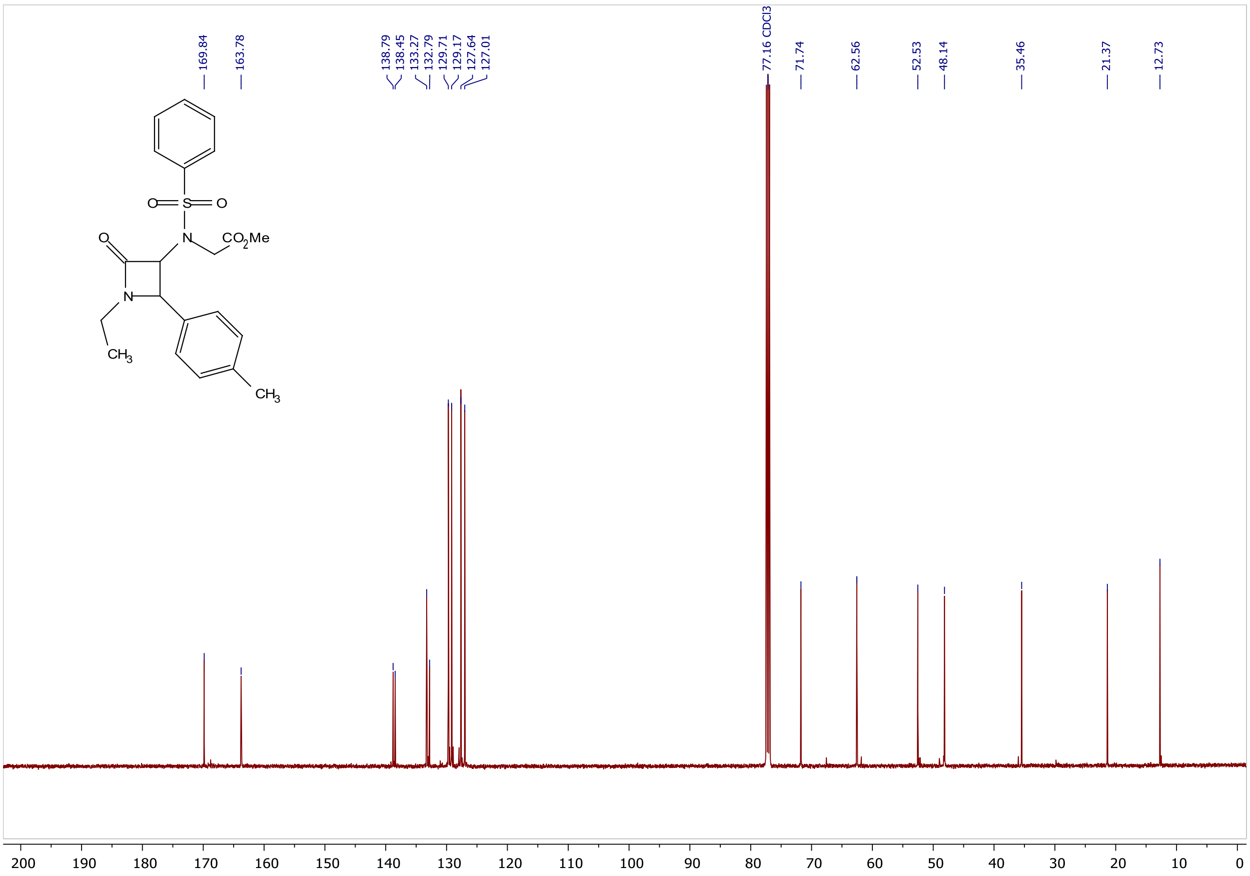

^{1}H and ^{13}C NMR spectra of compound **6b**

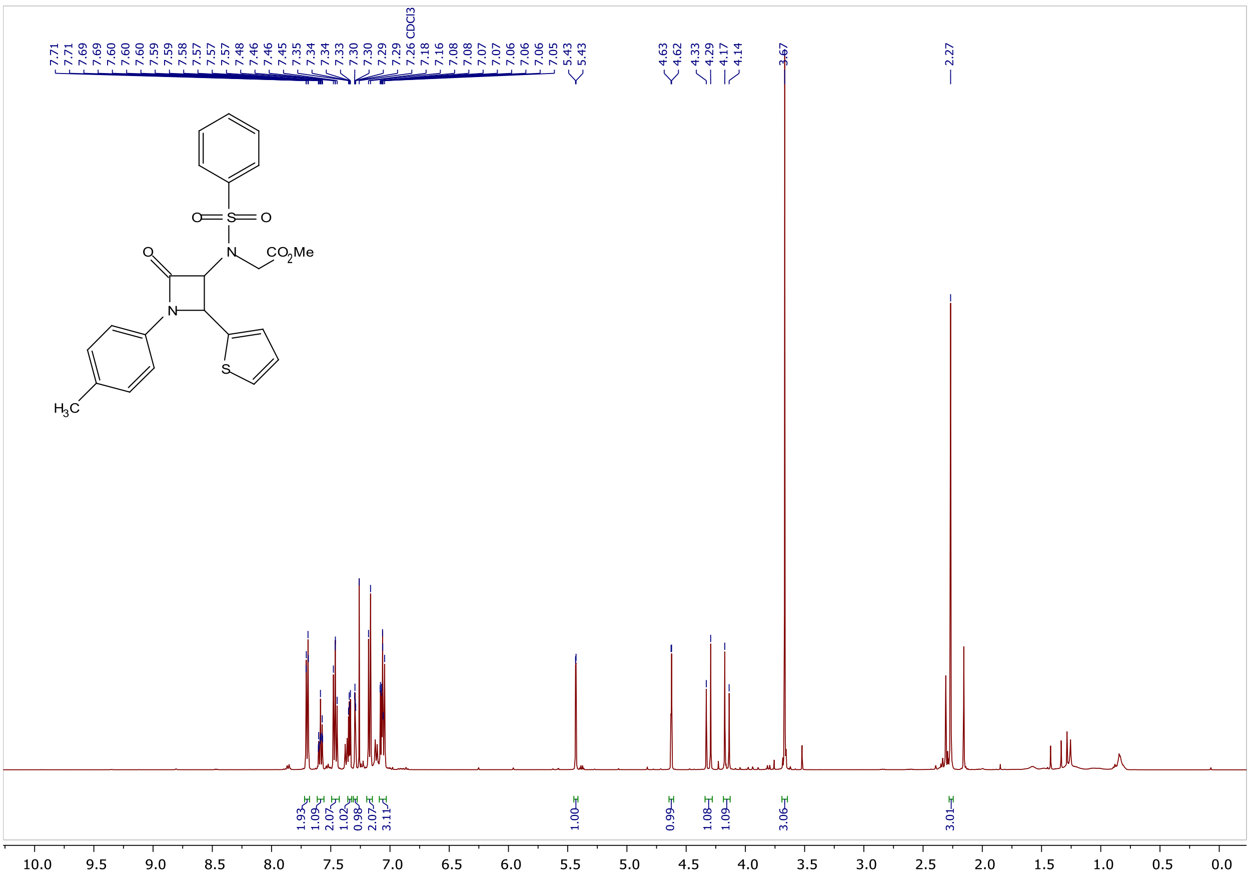

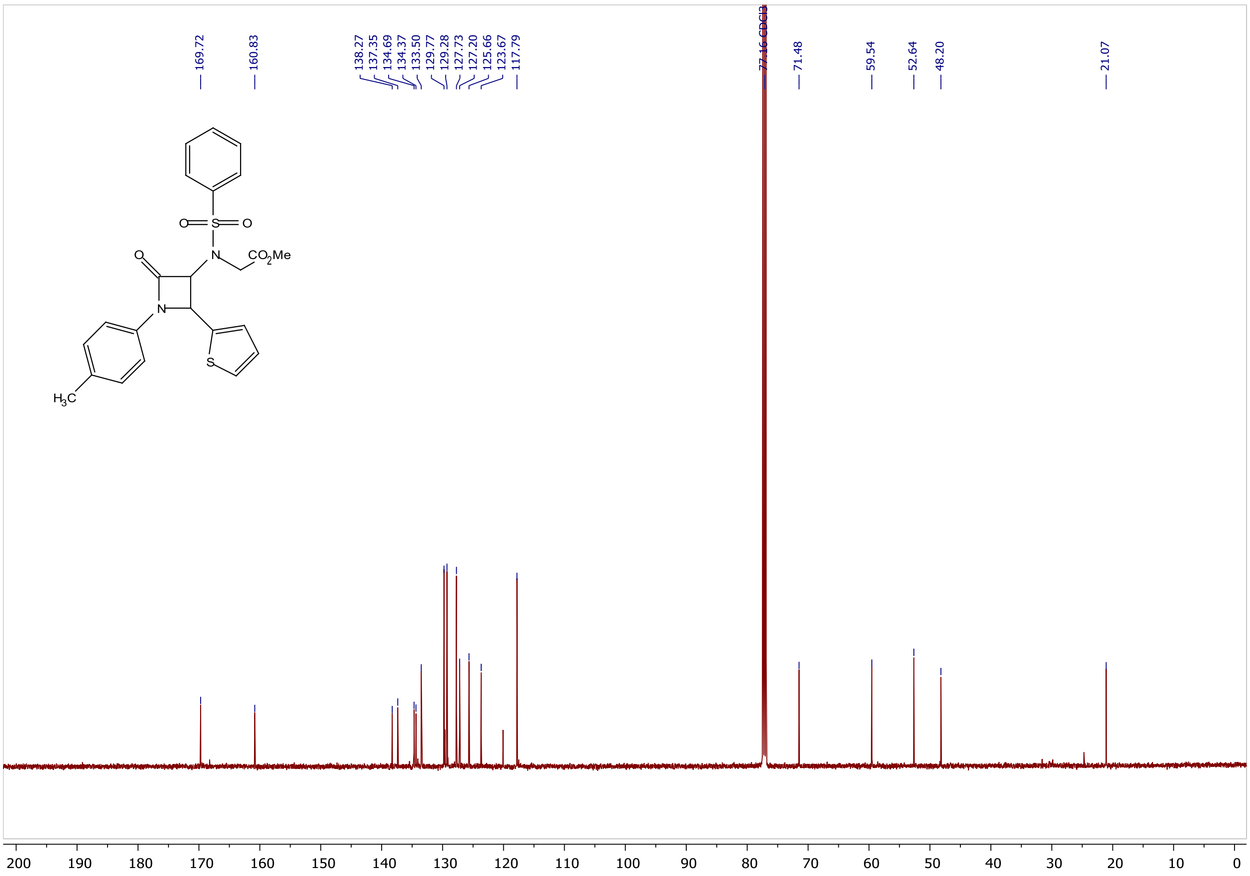

^{1}H and ^{13}C NMR spectra of compound **6c**

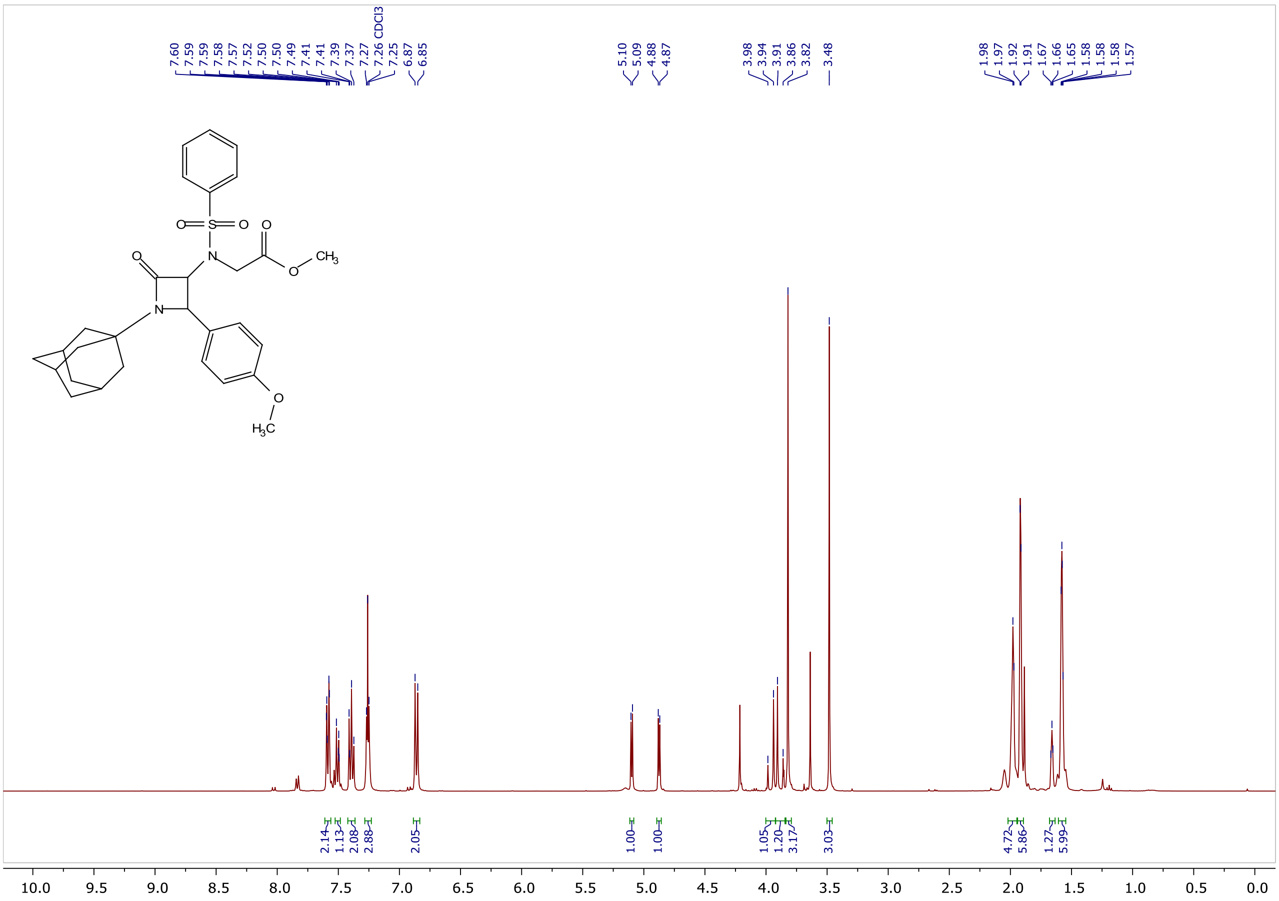

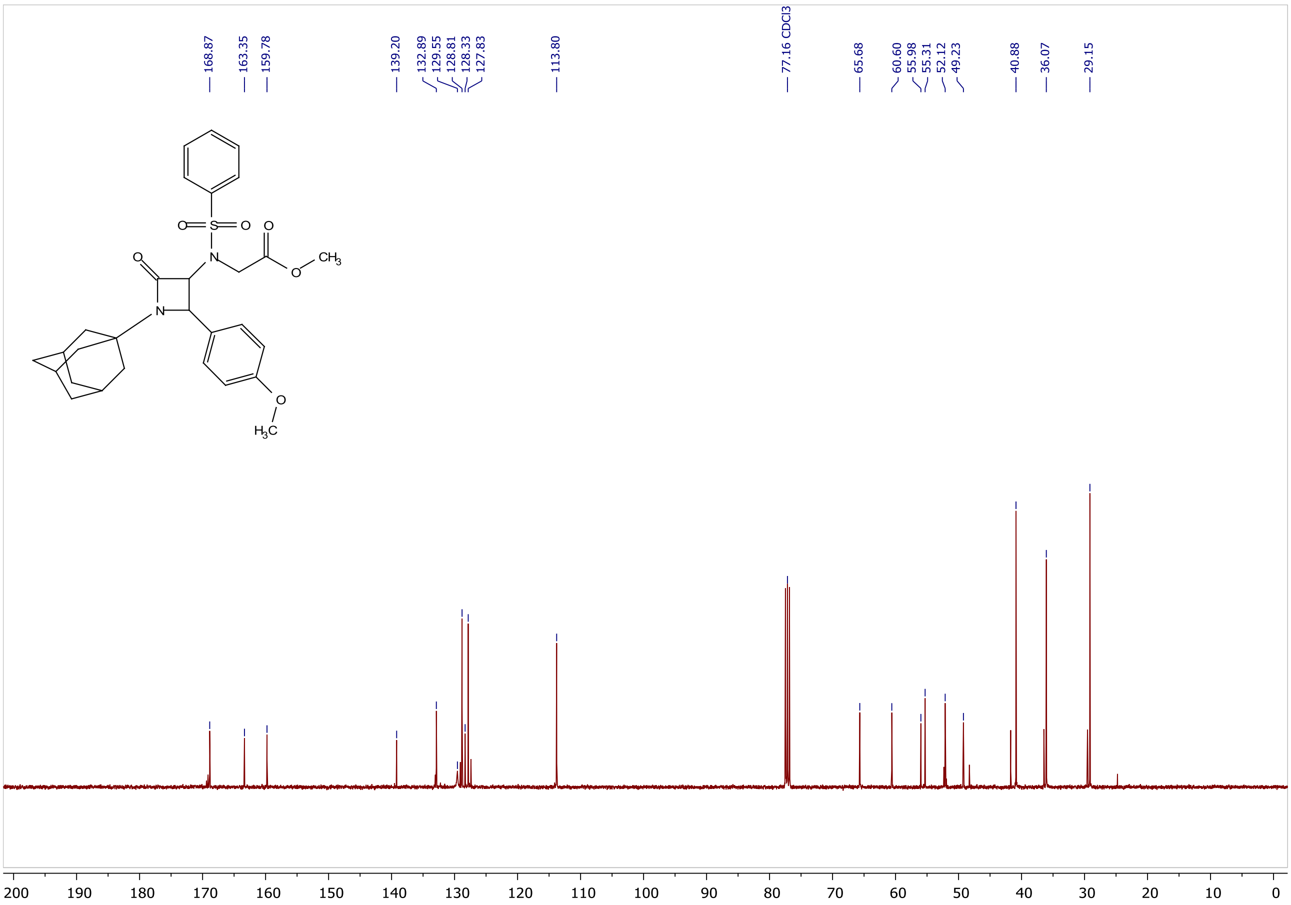

^{1}H, ^{13}C and ^{19}F NMR spectra of compound **6d**

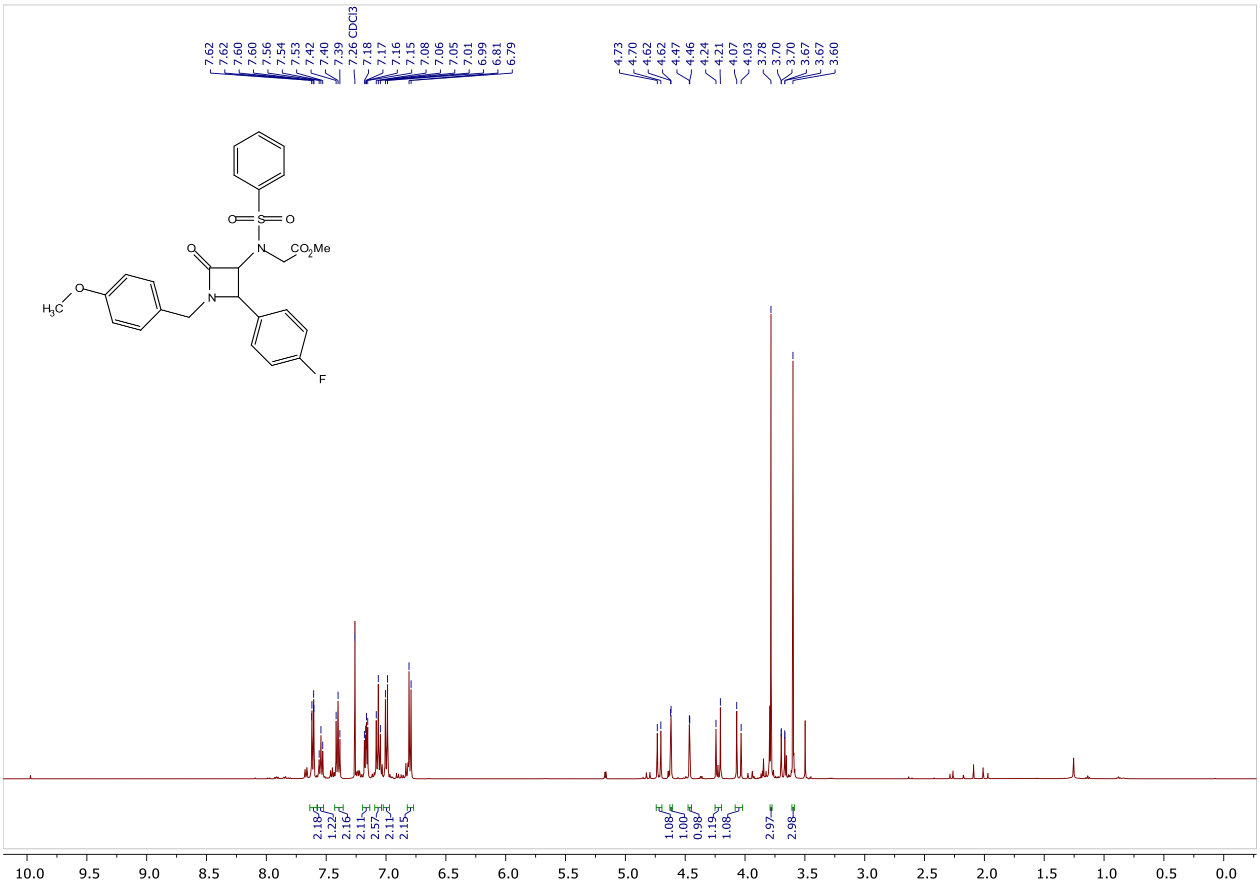

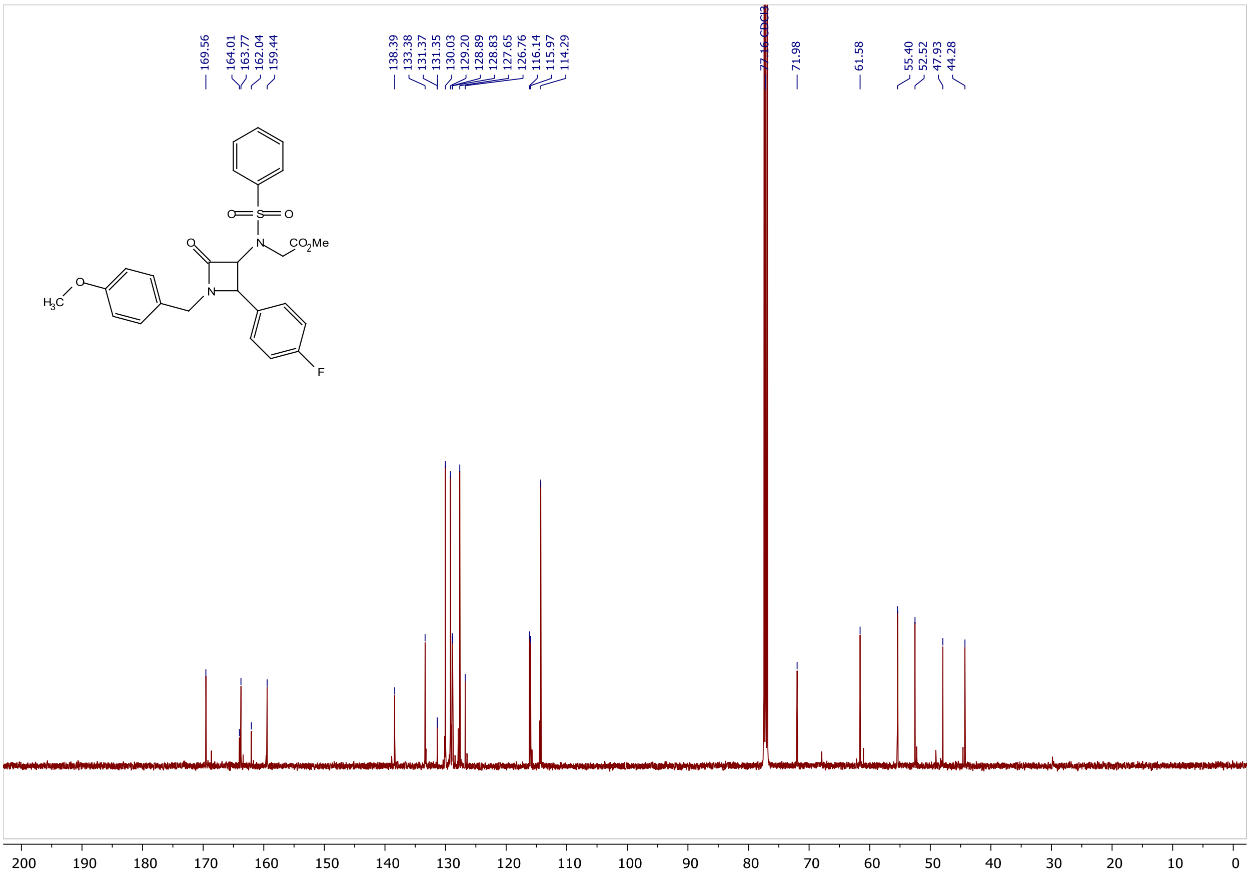
169.56
164.01
163.77
162.04
159.44
138.39
133.38
131.37
131.35
130.03
129.20
128.89
128.83
127.65
126.76
116.14
115.97
114.29
77.16 CDCl3
71.98
61.58
55.40
52.52
47.93
44.28
O
S
N
CO2Me
H3C
F
200 190 180 170 160 150 140 130 120 110 100 90 80 70 60 50 40 30 20 10 0

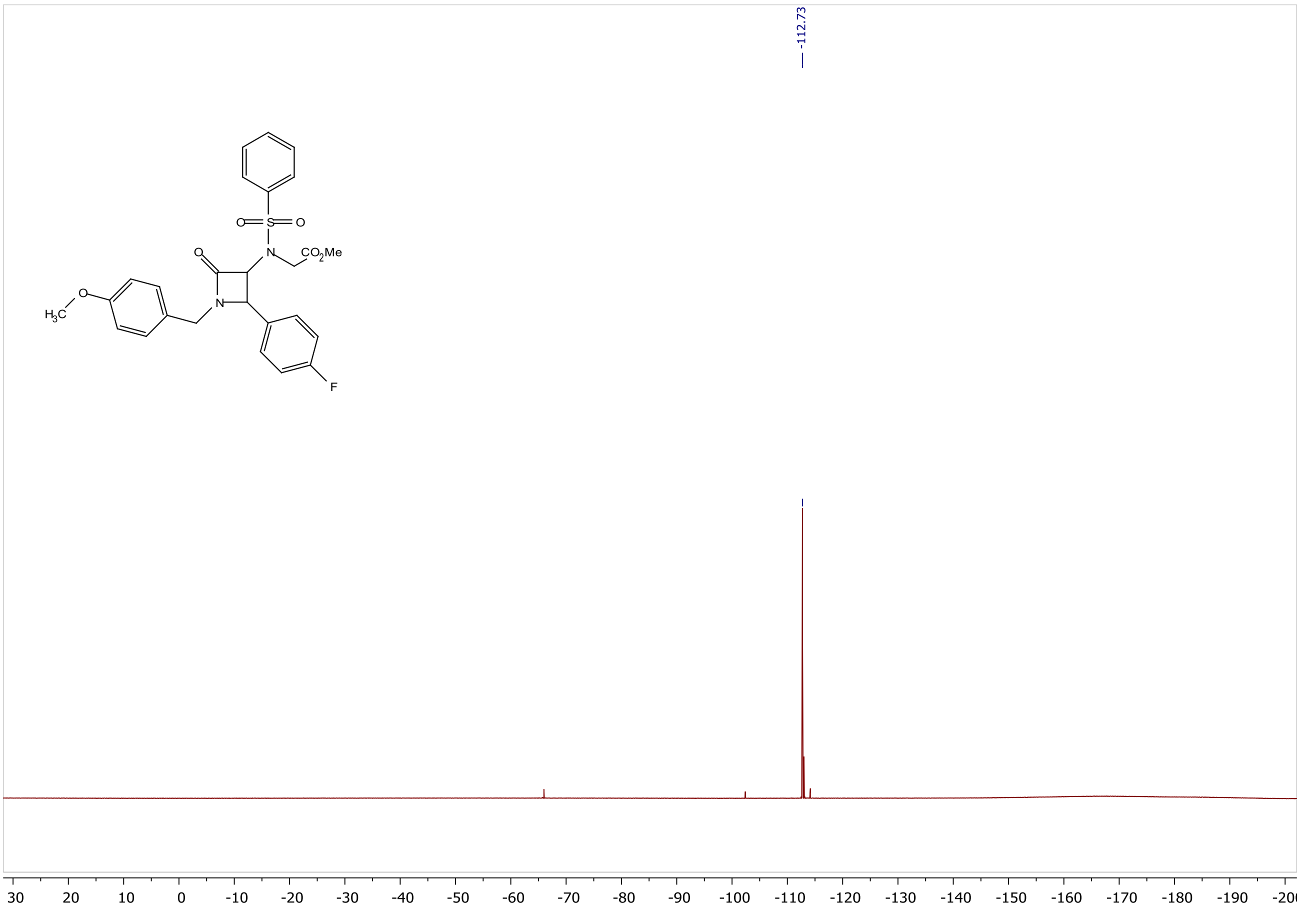
-112.73
O
S
N
CO2Me
H3C
F
30 20 10 0 -10 -20 -30 -40 -50 -60 -70 -80 -90 -100 -110 -120 -130 -140 -150 -160 -170 -180 -190

^{1}H and ^{13}C NMR spectra of compound **6e**

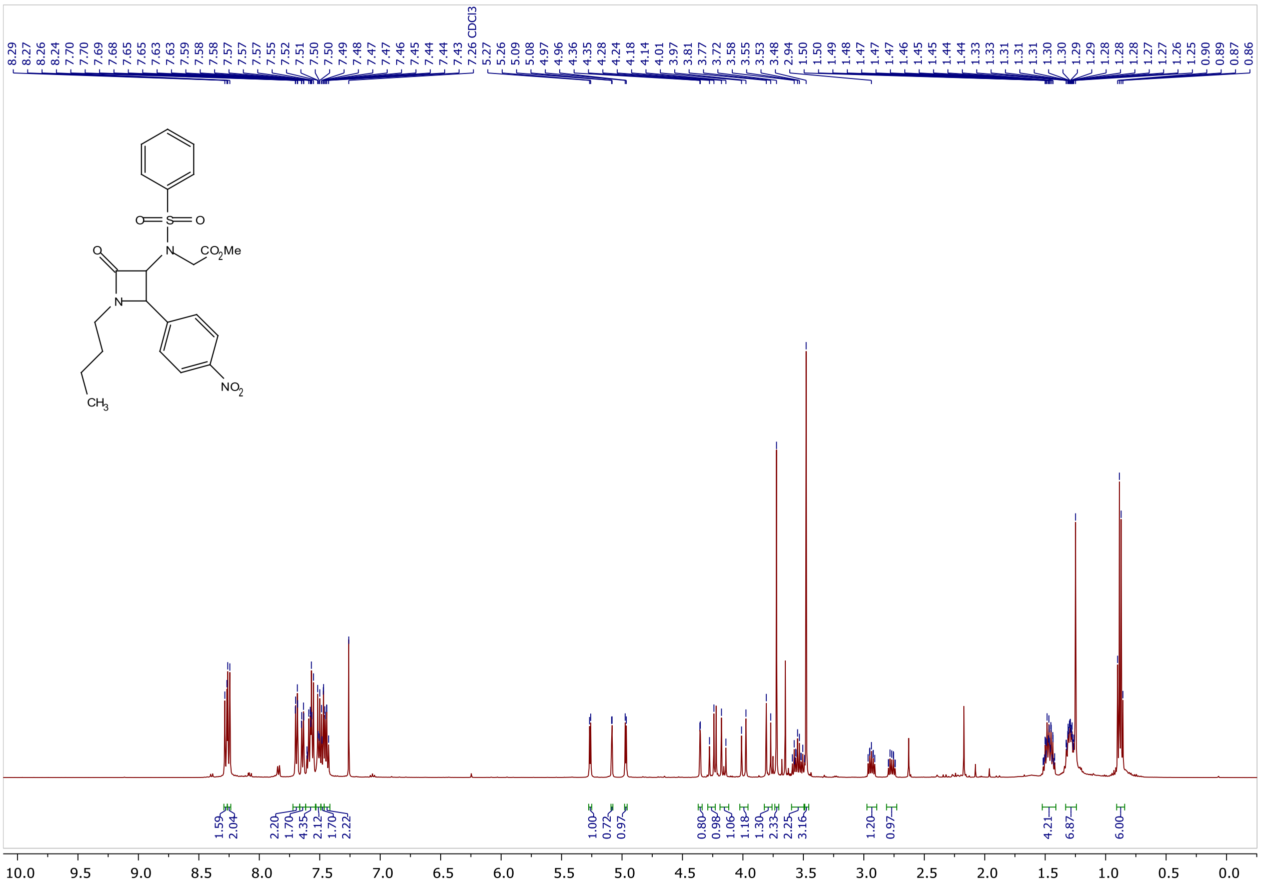

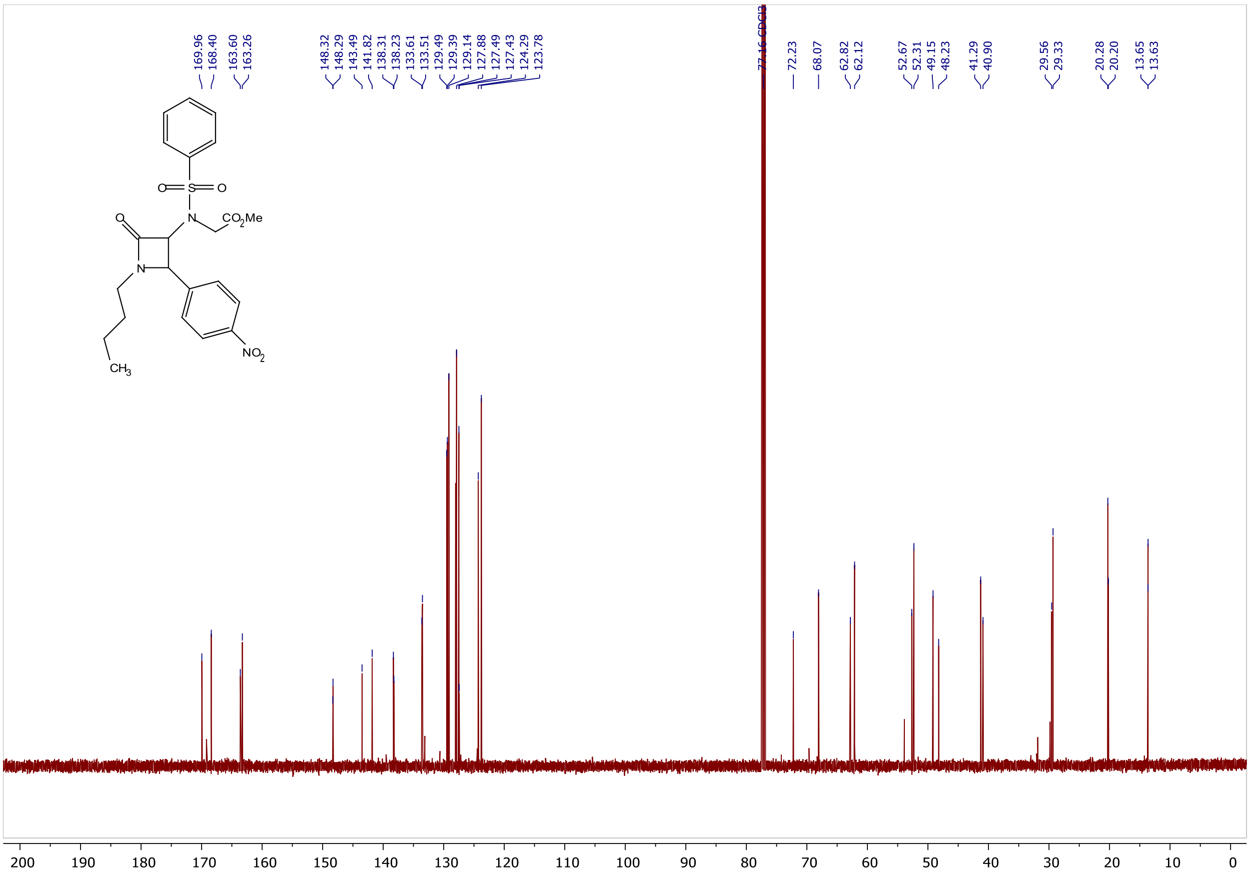

^{1}H, ^{13}C and ^{19}F NMR spectra of compound **6f**

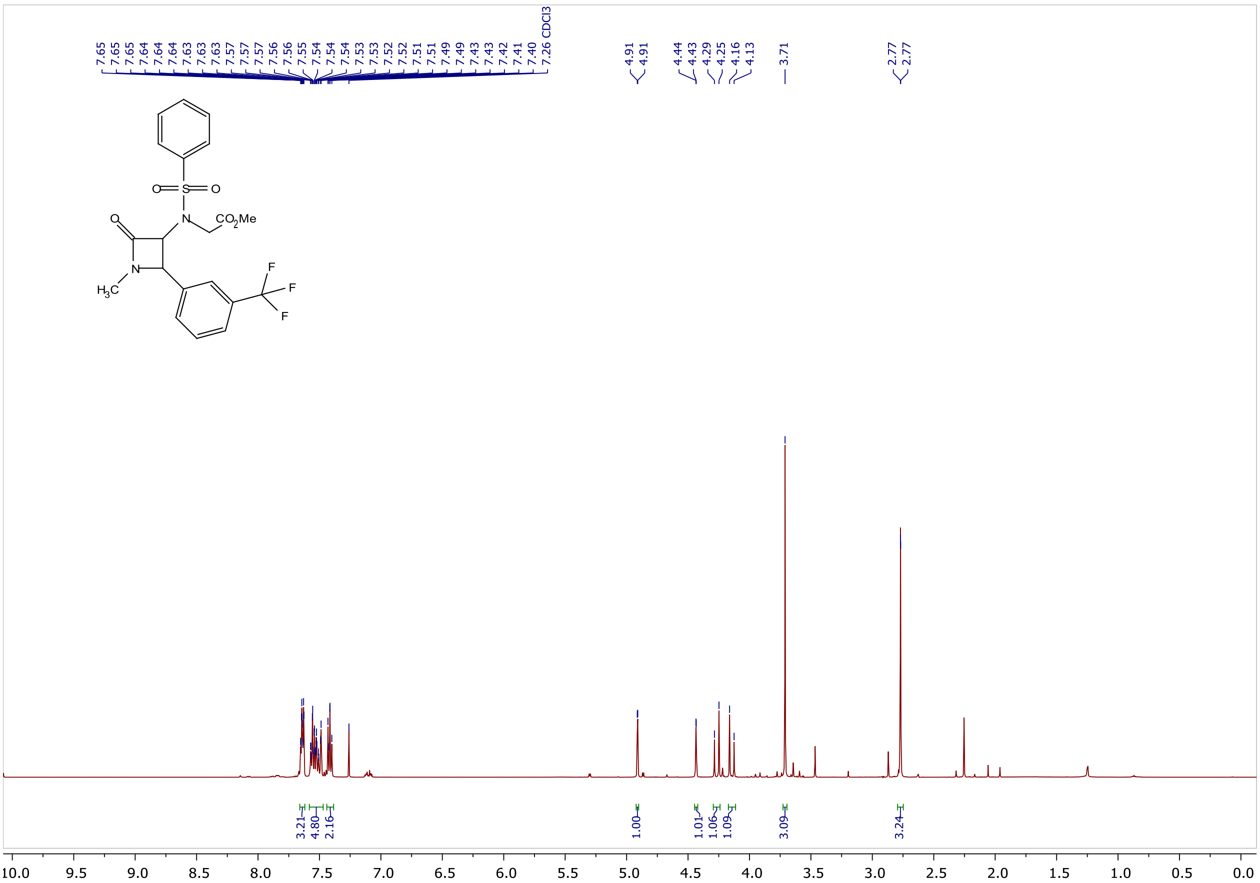

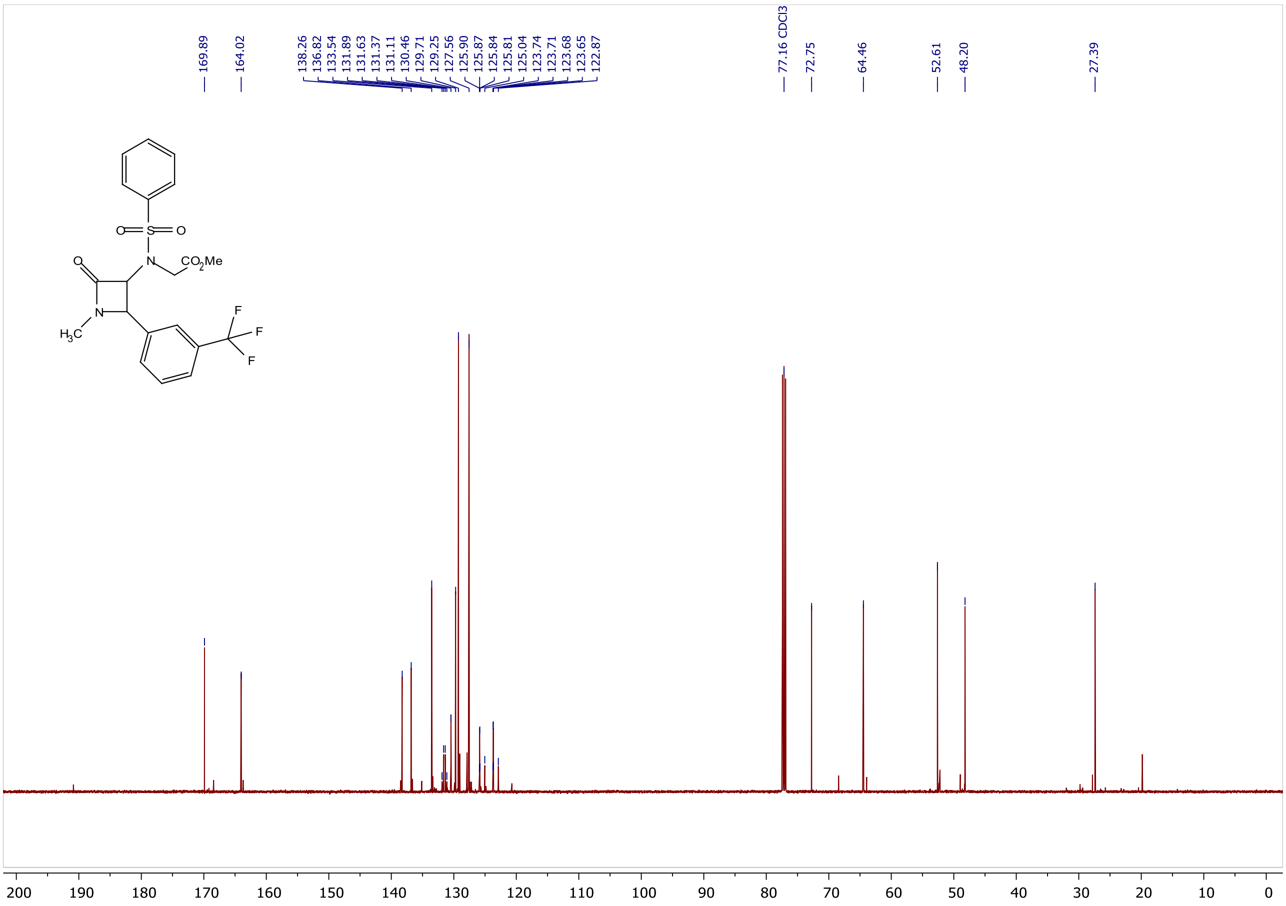

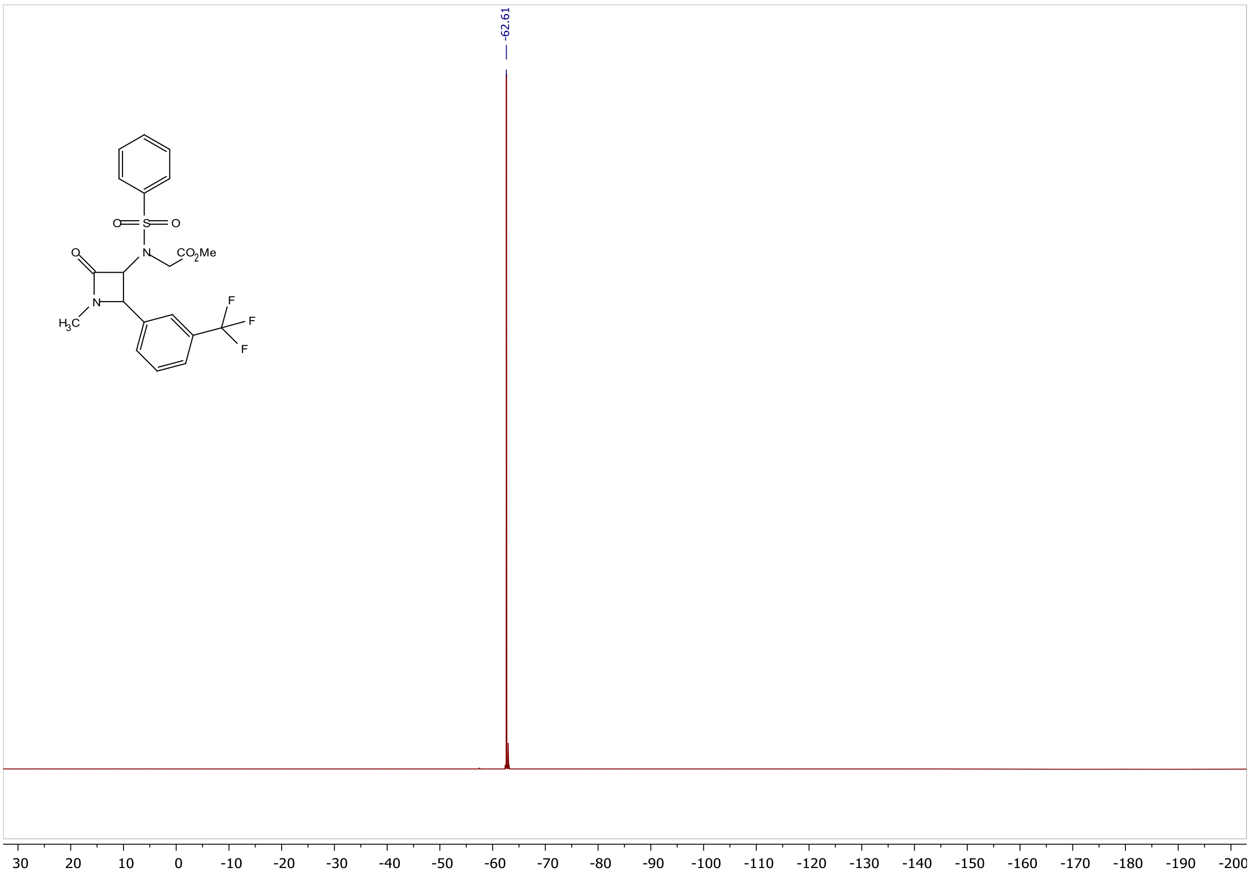

^{1}H and ^{13}C NMR spectra of compound **6g**

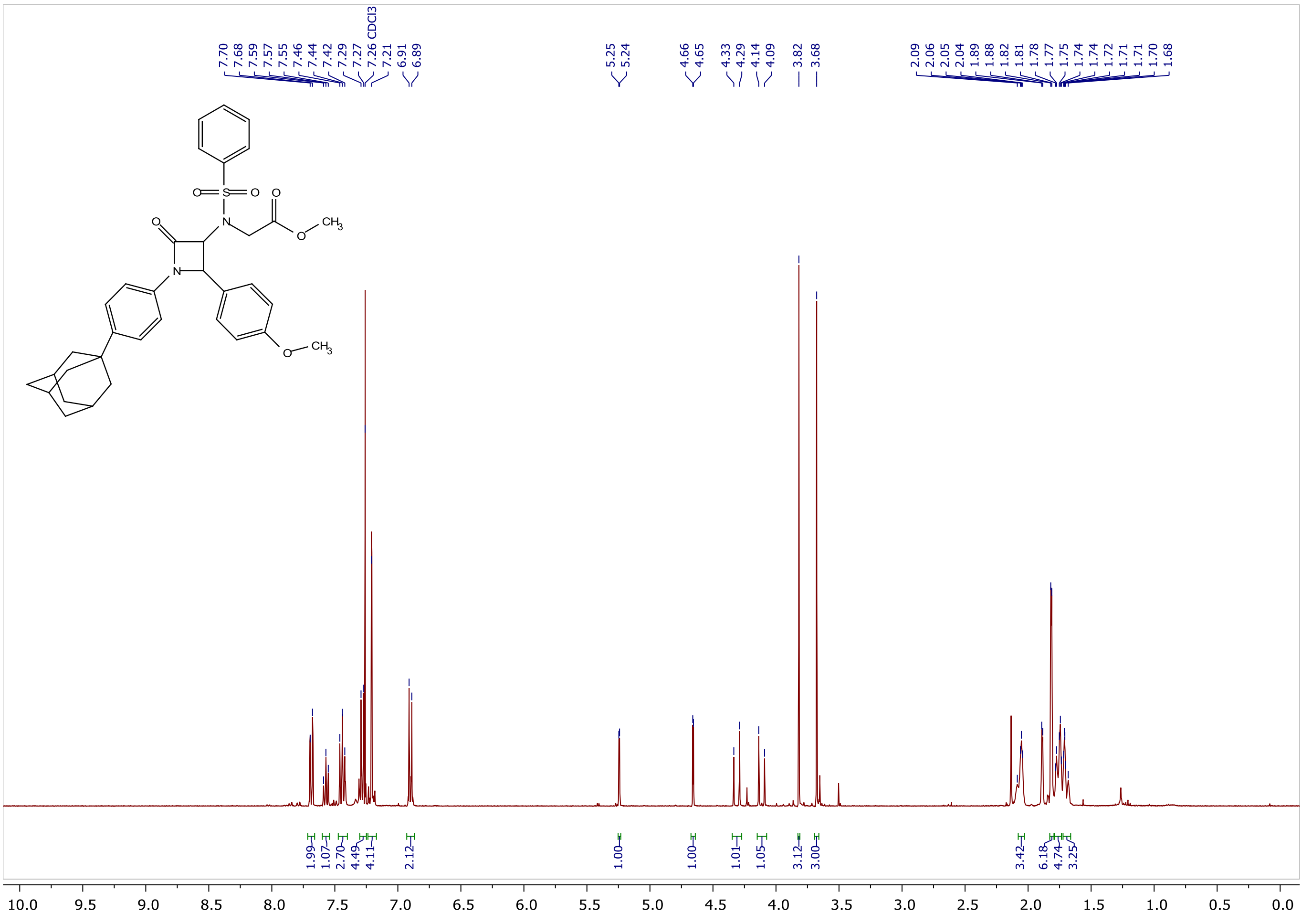

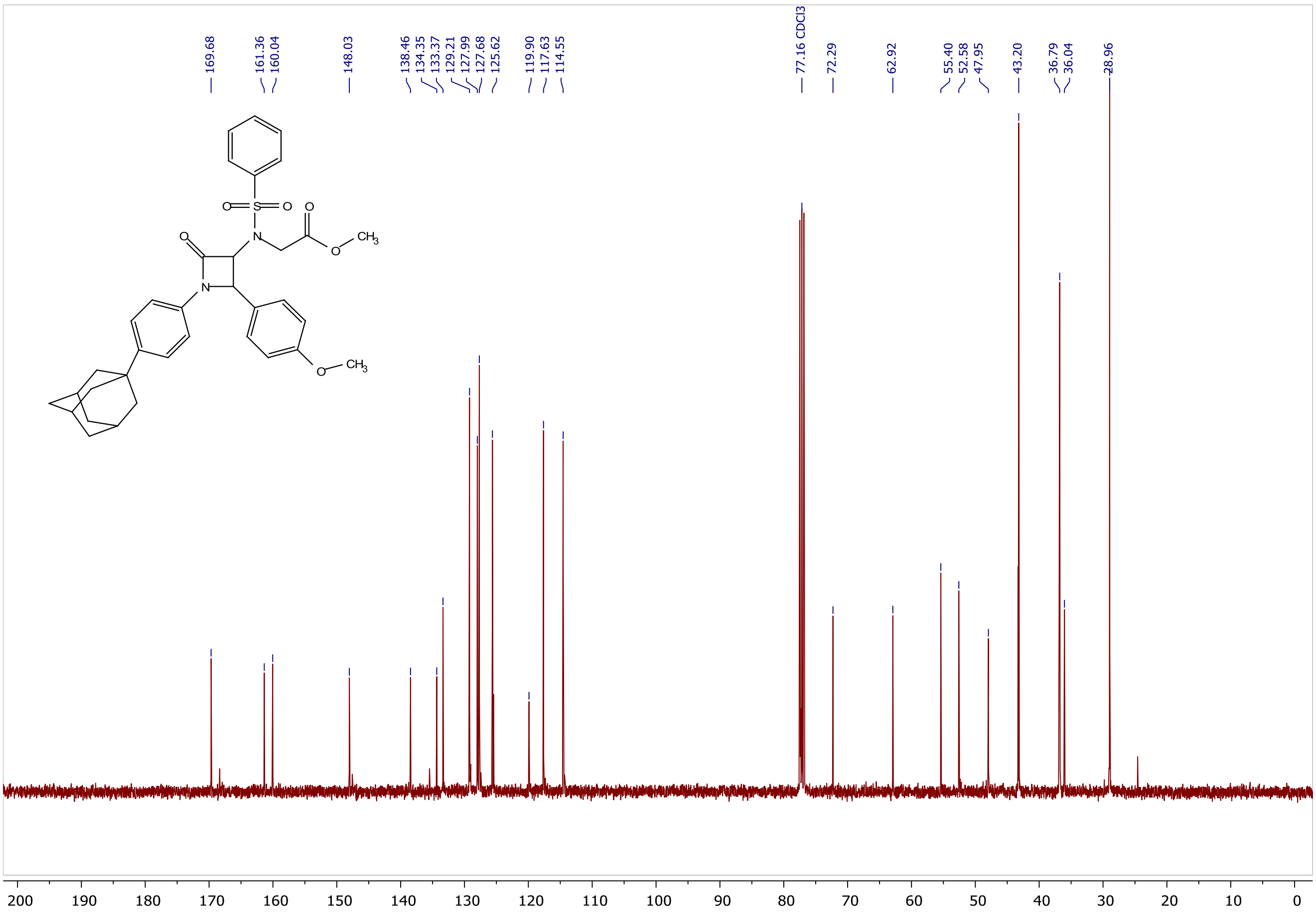

^{1}H, ^{13}C and ^{19}F NMR spectra of compound **6h**

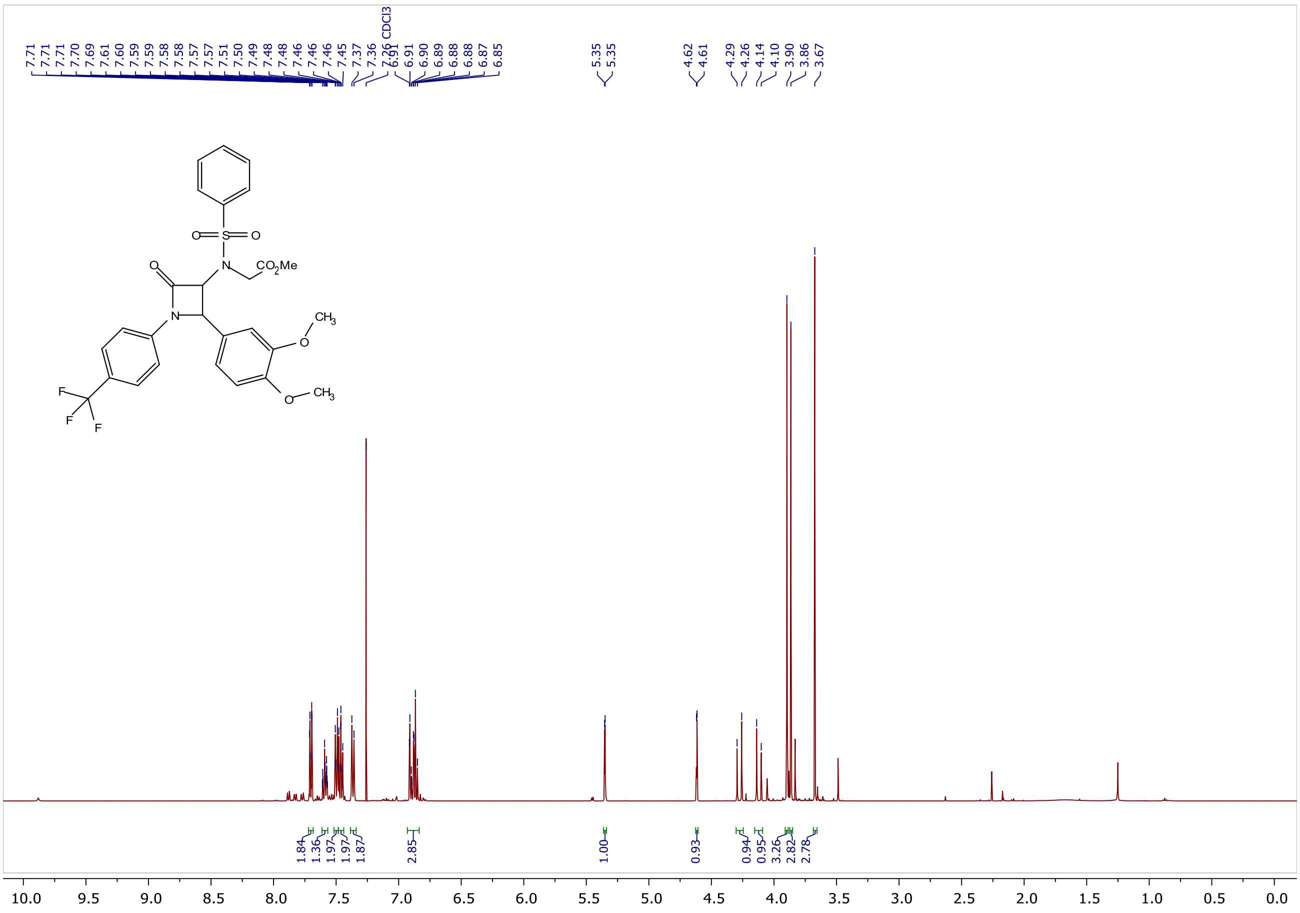

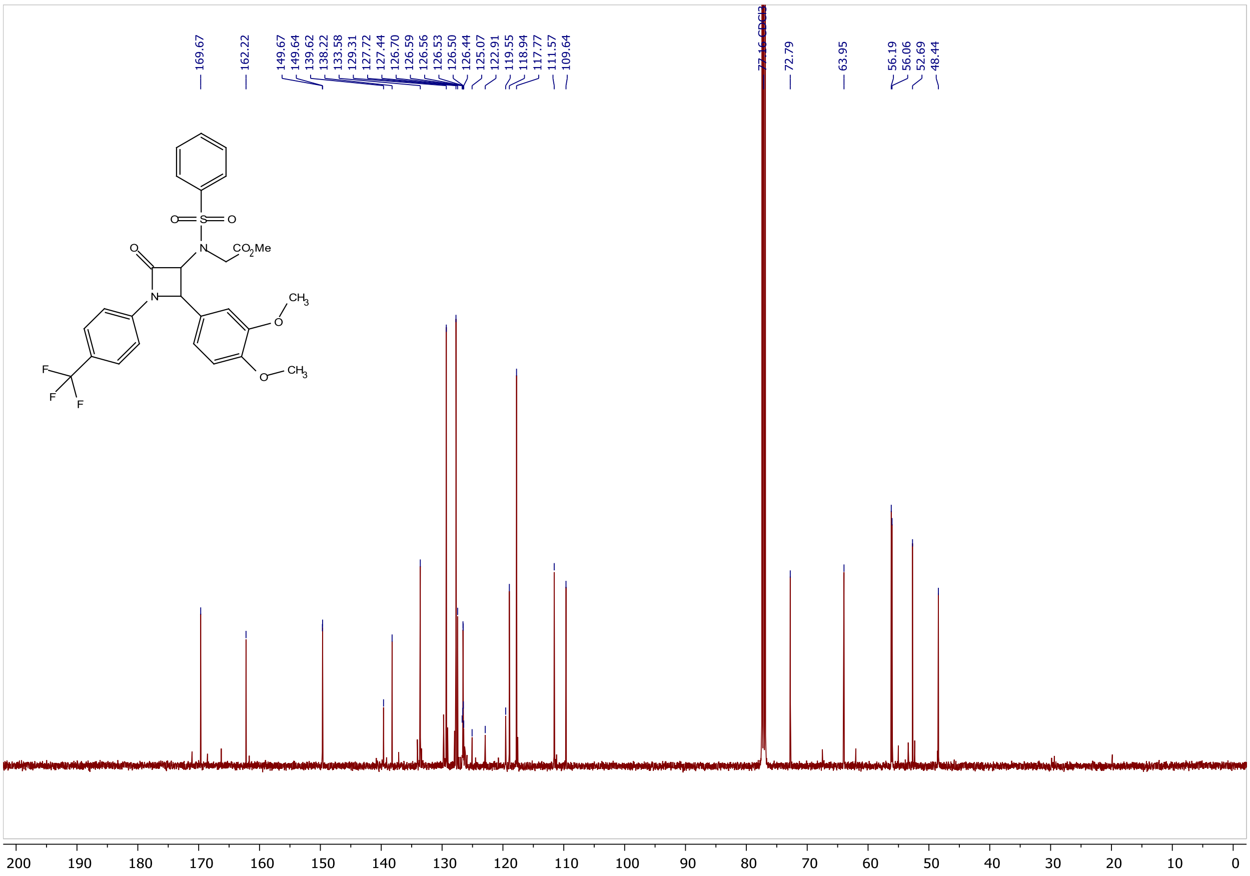
169.67
162.22
149.67
149.64
139.62
138.22
133.58
129.31
127.72
127.44
126.70
126.59
126.56
126.53
126.50
126.44
125.07
122.91
119.55
118.94
117.77
111.57
109.64
77.16 CDCl3
72.79
63.95
56.19
56.06
52.69
48.44
CO2Me
CH3
CH3
200 190 180 170 160 150 140 130 120 110 100 90 80 70 60 50 40 30 20 10 0

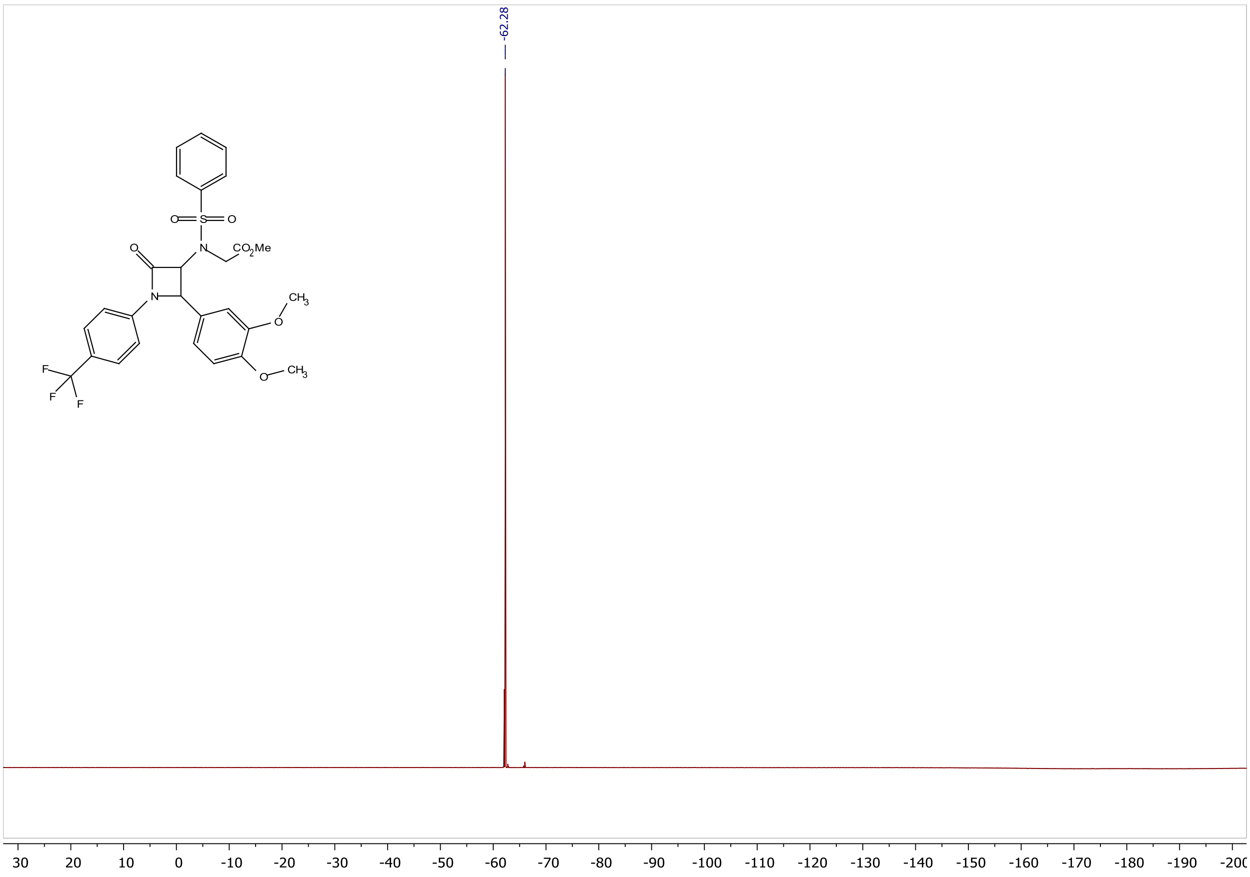
-62.28
CO2Me
CH3
CH3
30 20 10 0 -10 -20 -30 -40 -50 -60 -70 -80 -90 -100 -110 -120 -130 -140 -150 -160 -170 -180 -190 -200

^{1}H and ^{13}C NMR spectra of compound **6i**

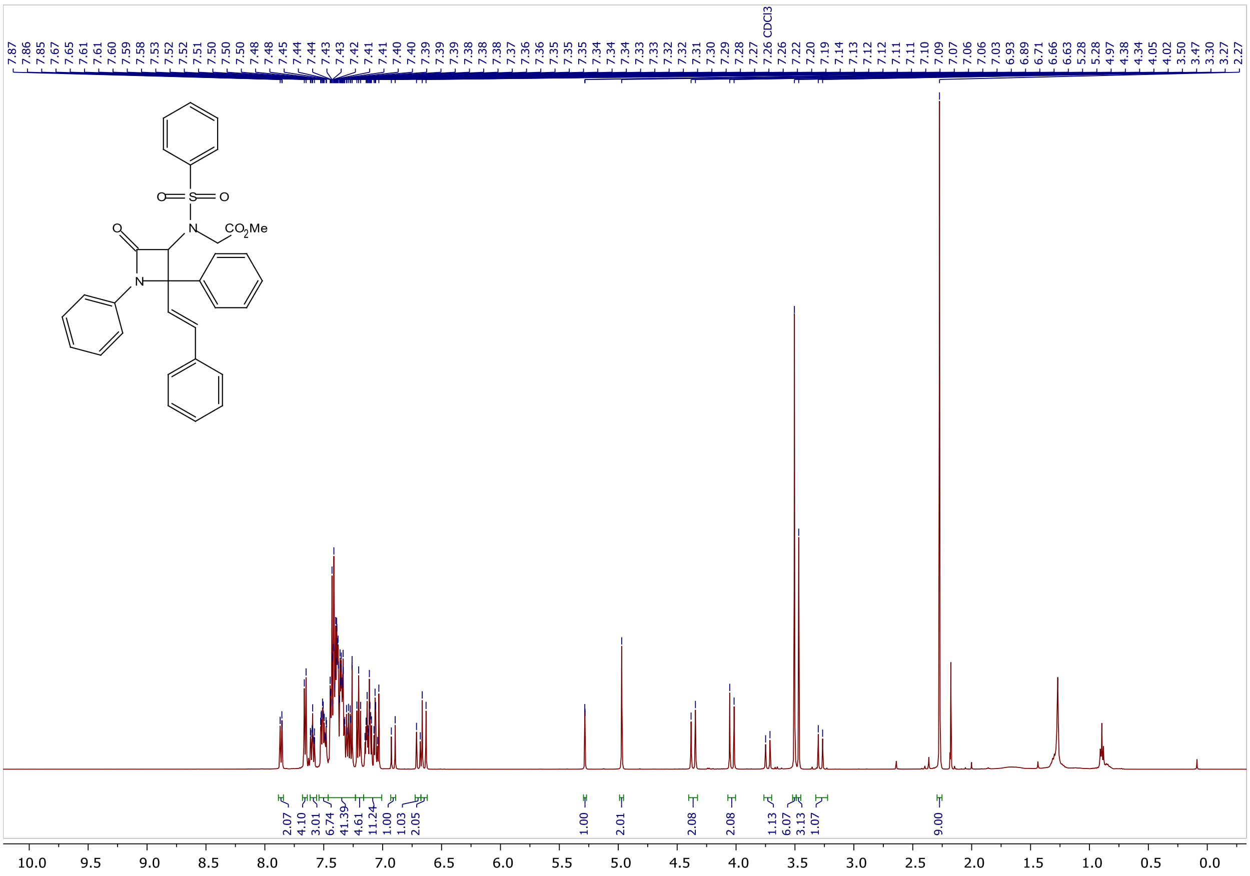

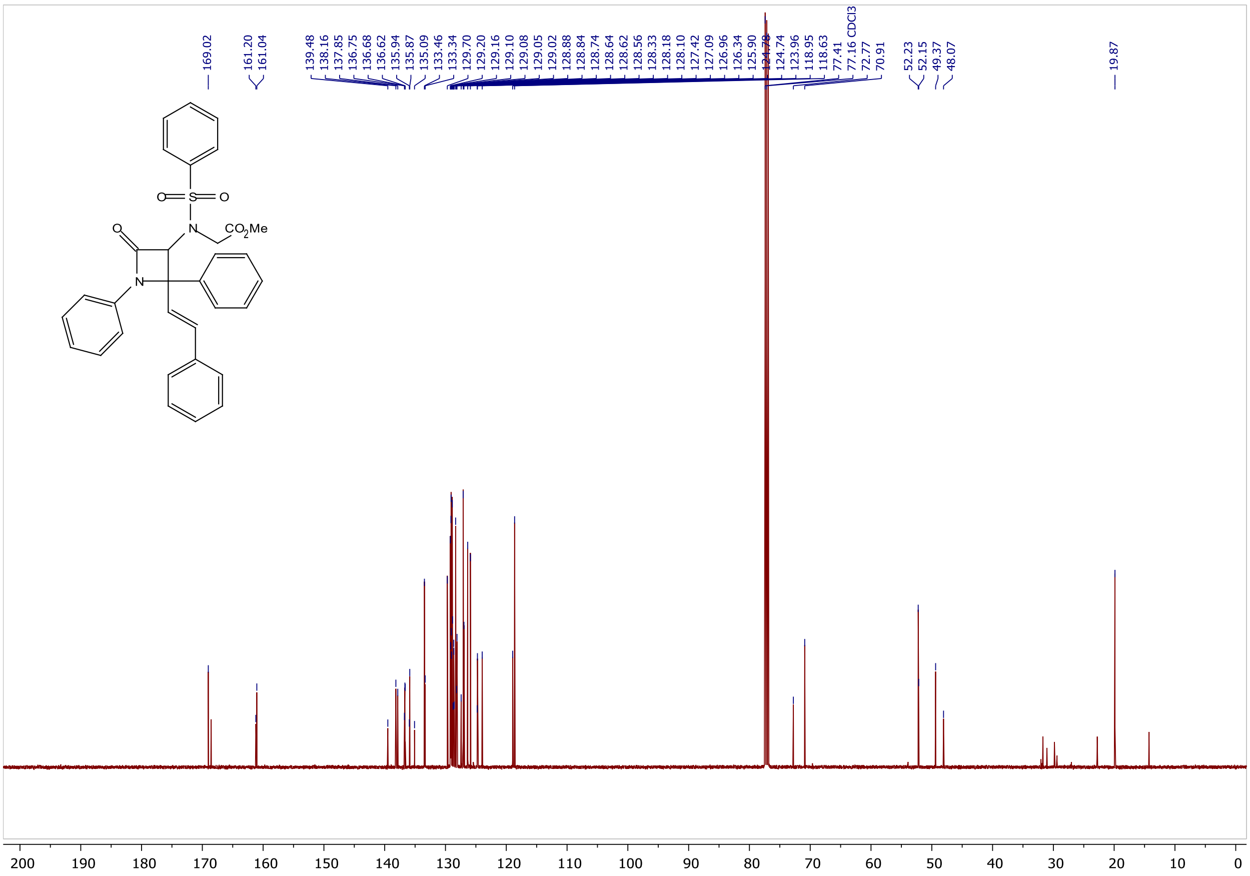

^{1}H and ^{13}C NMR spectra of compound **6j**-minor diastereomer

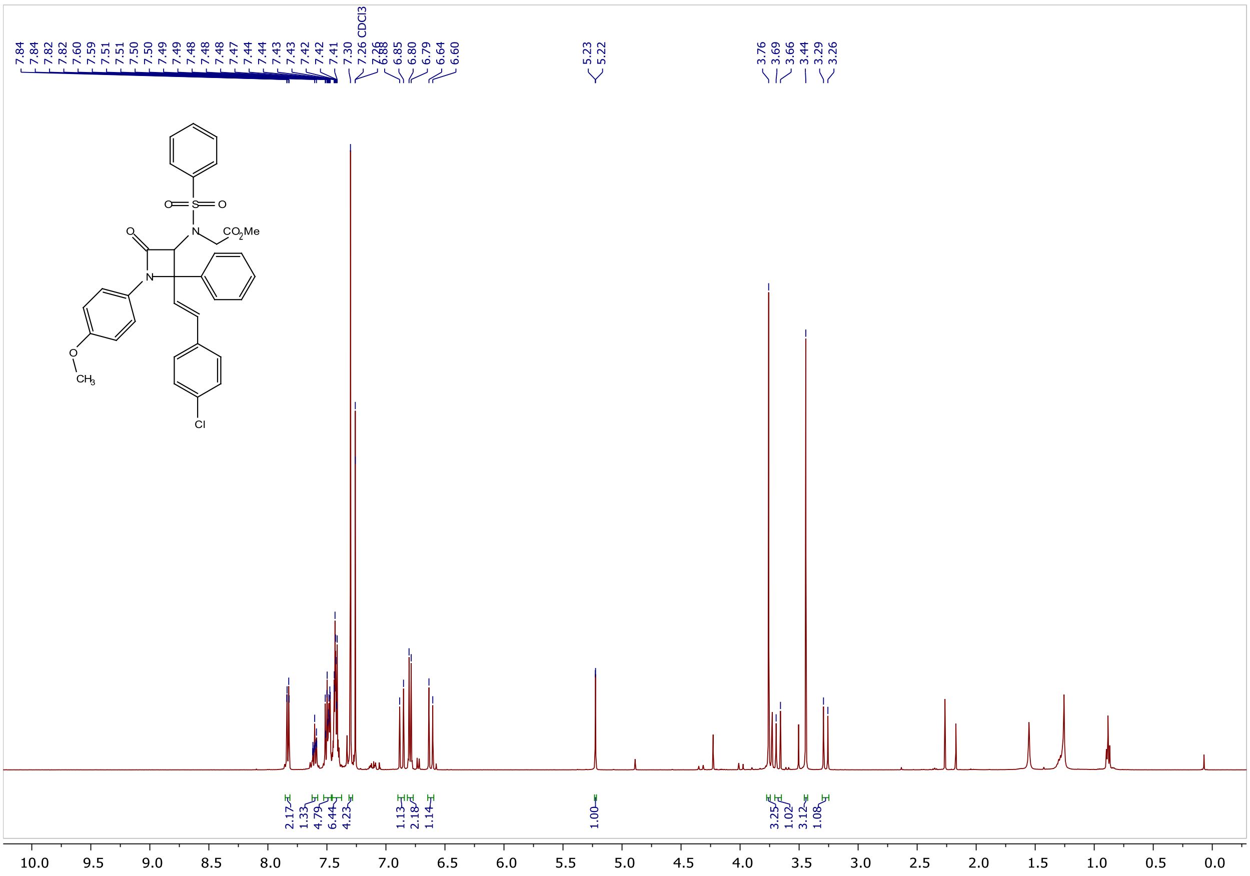

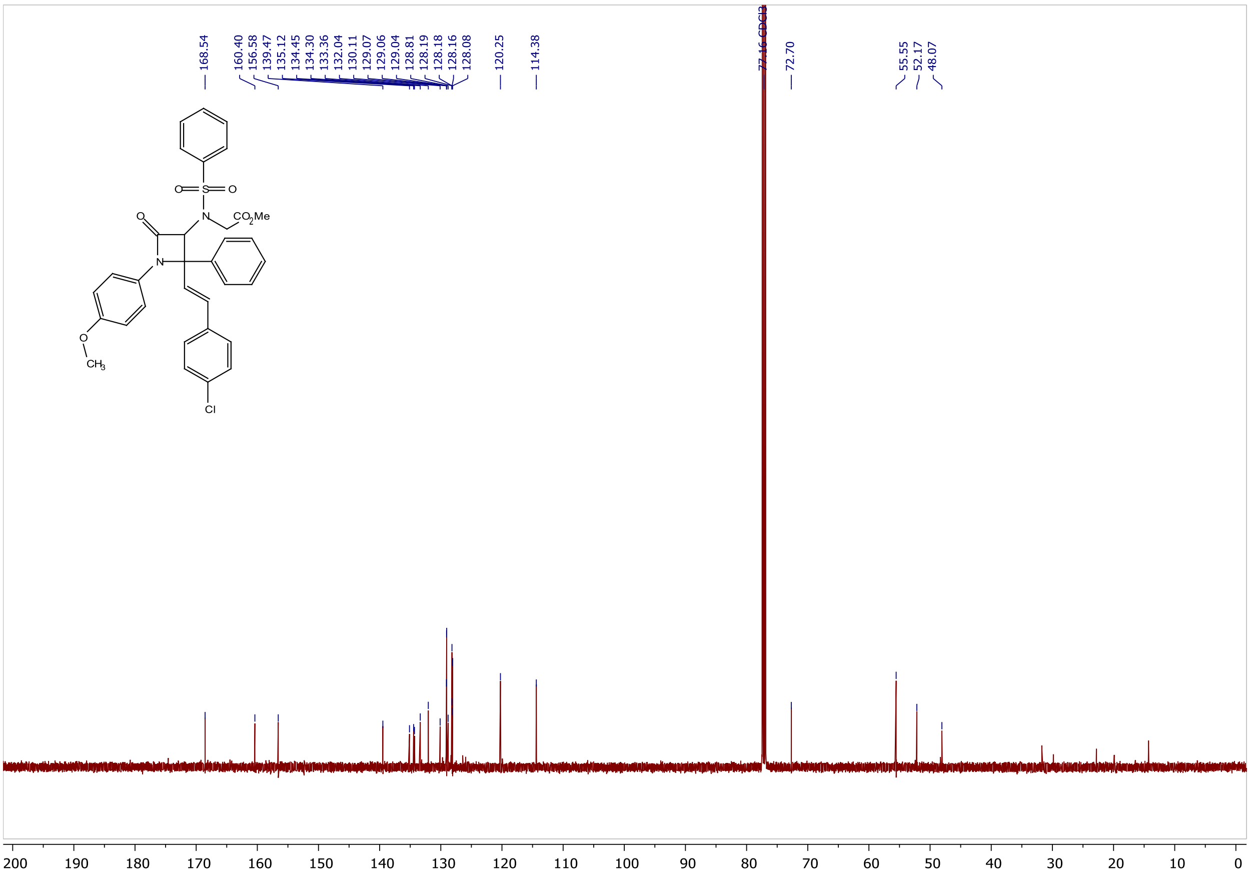

^{1}H and ^{13}C NMR spectra of compound **6j**-major diastereomer

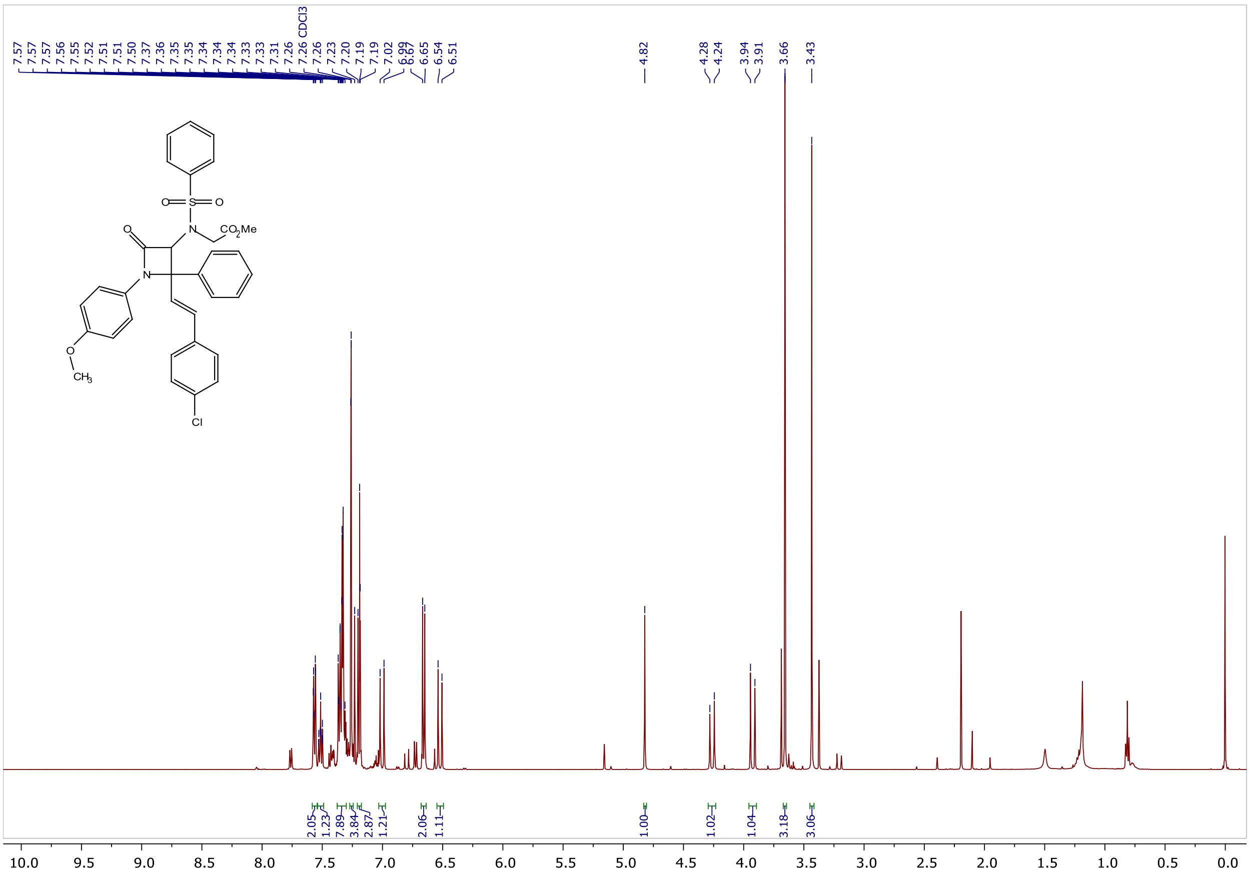

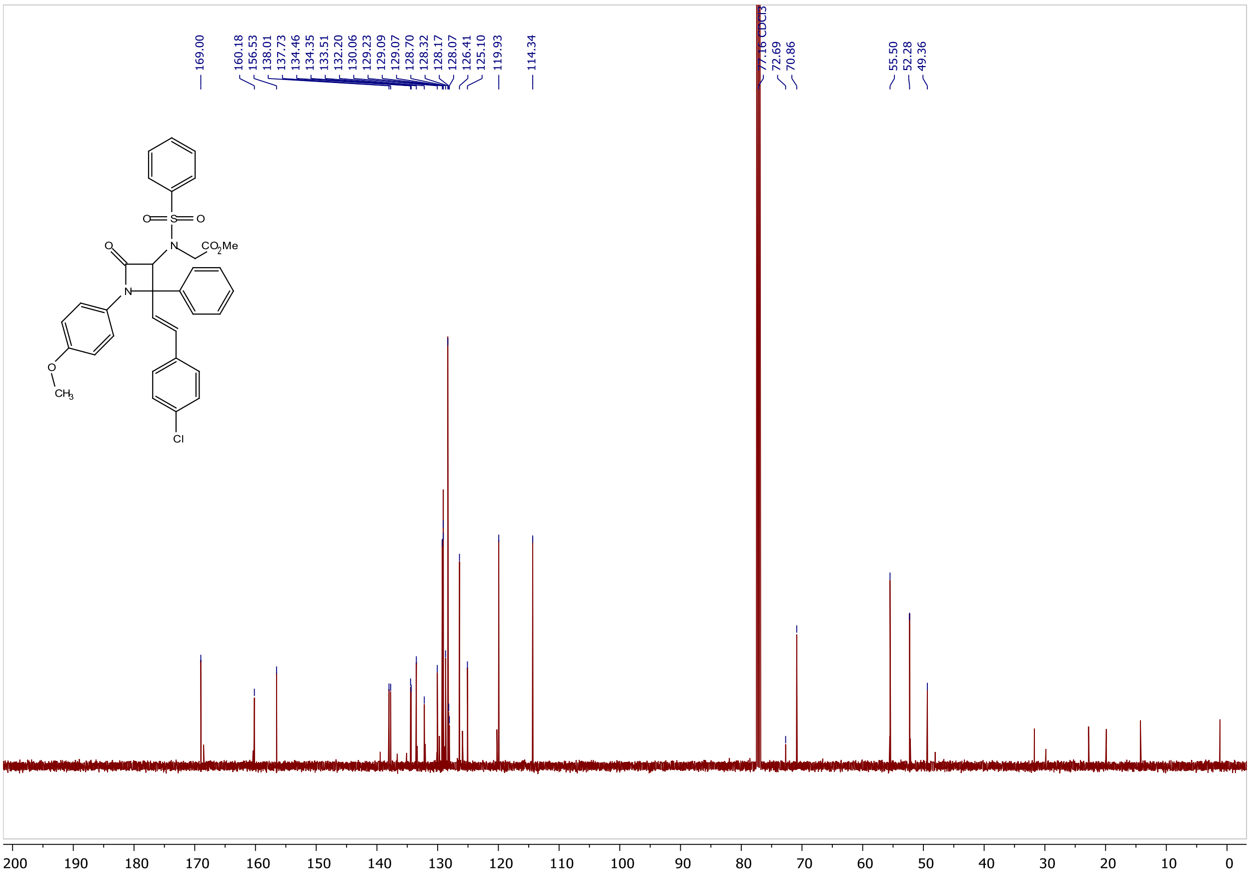

^{1}H and ^{13}C NMR spectra of compound **6k**

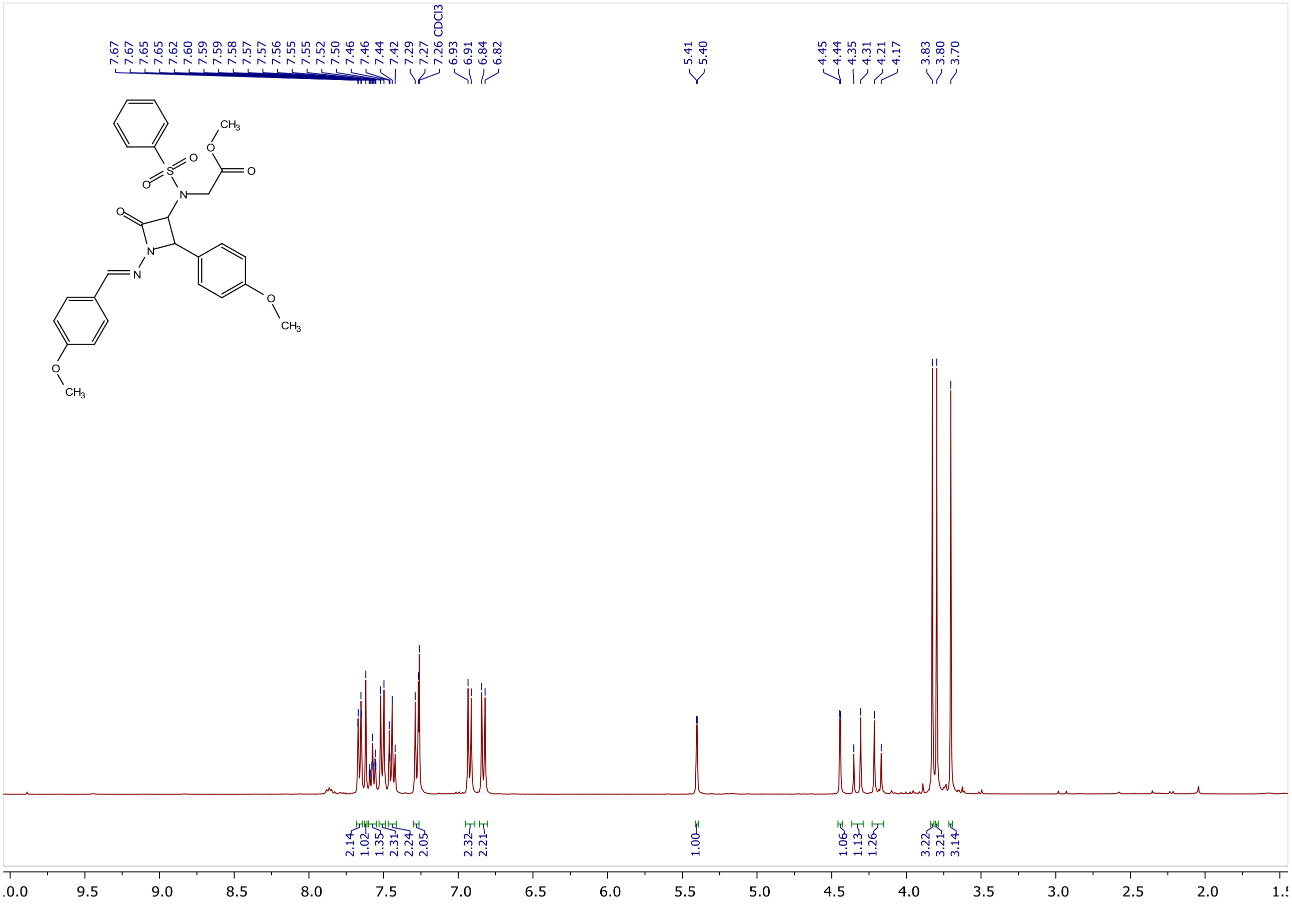

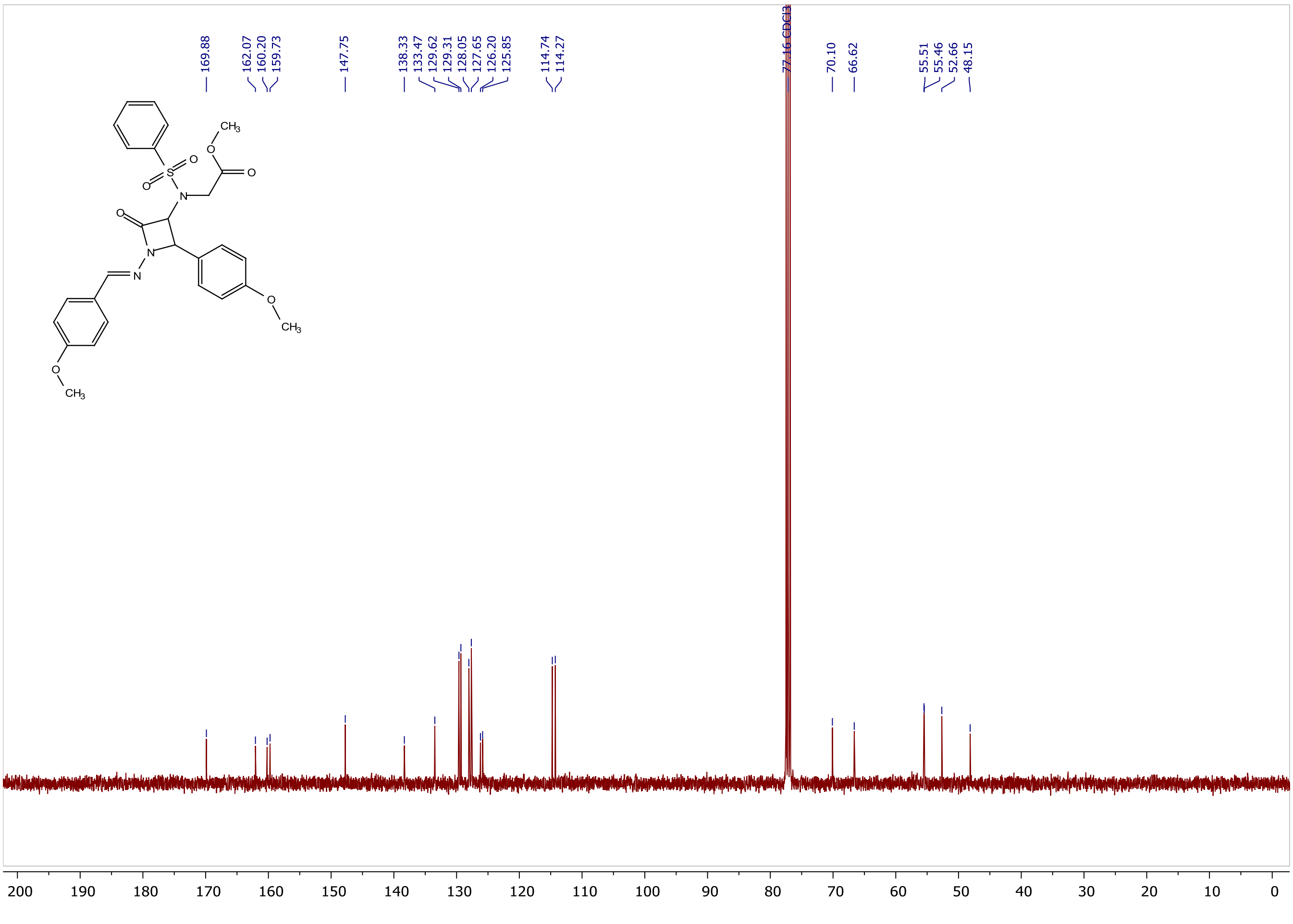

^{1}H and ^{13}C NMR spectra of compound **6l**

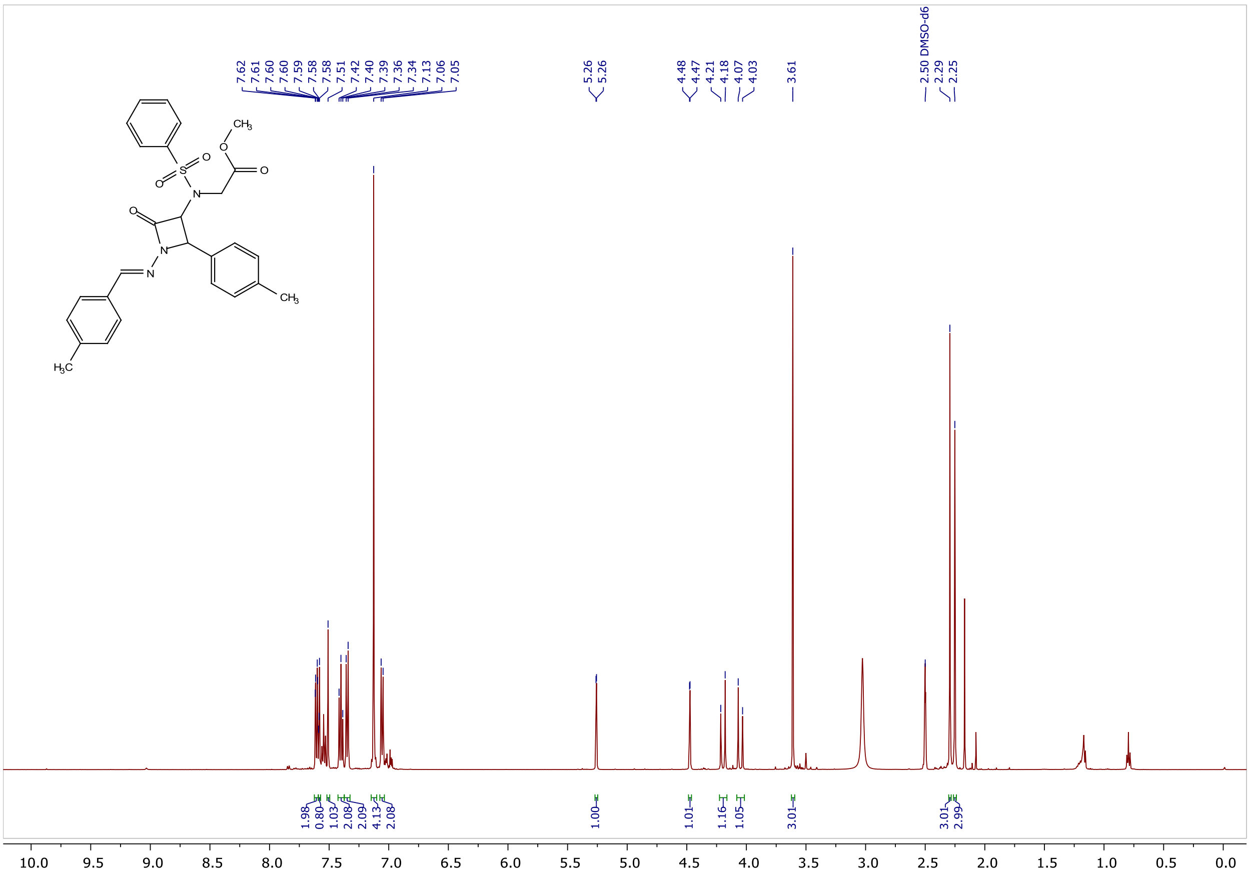

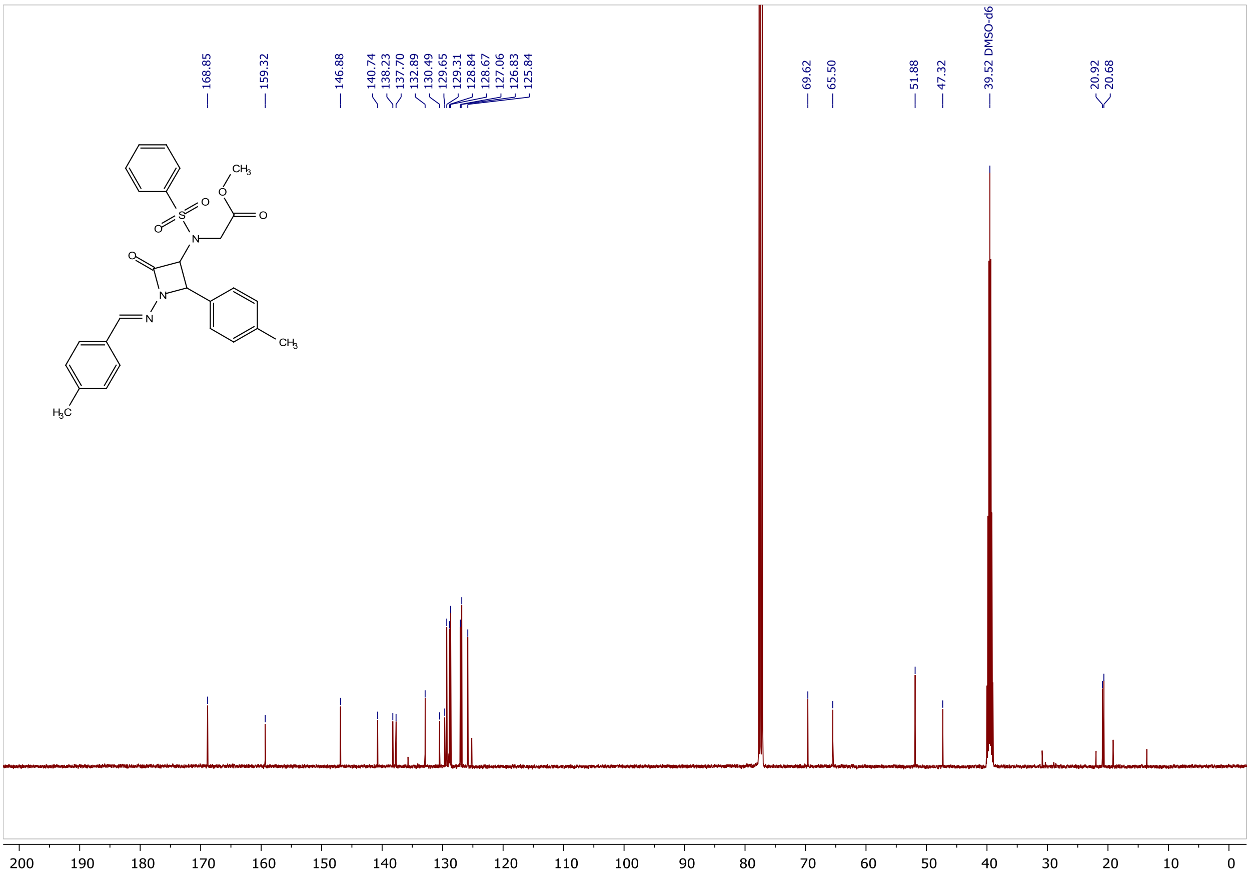

^{1}H and ^{13}C NMR spectra of compound **6m**

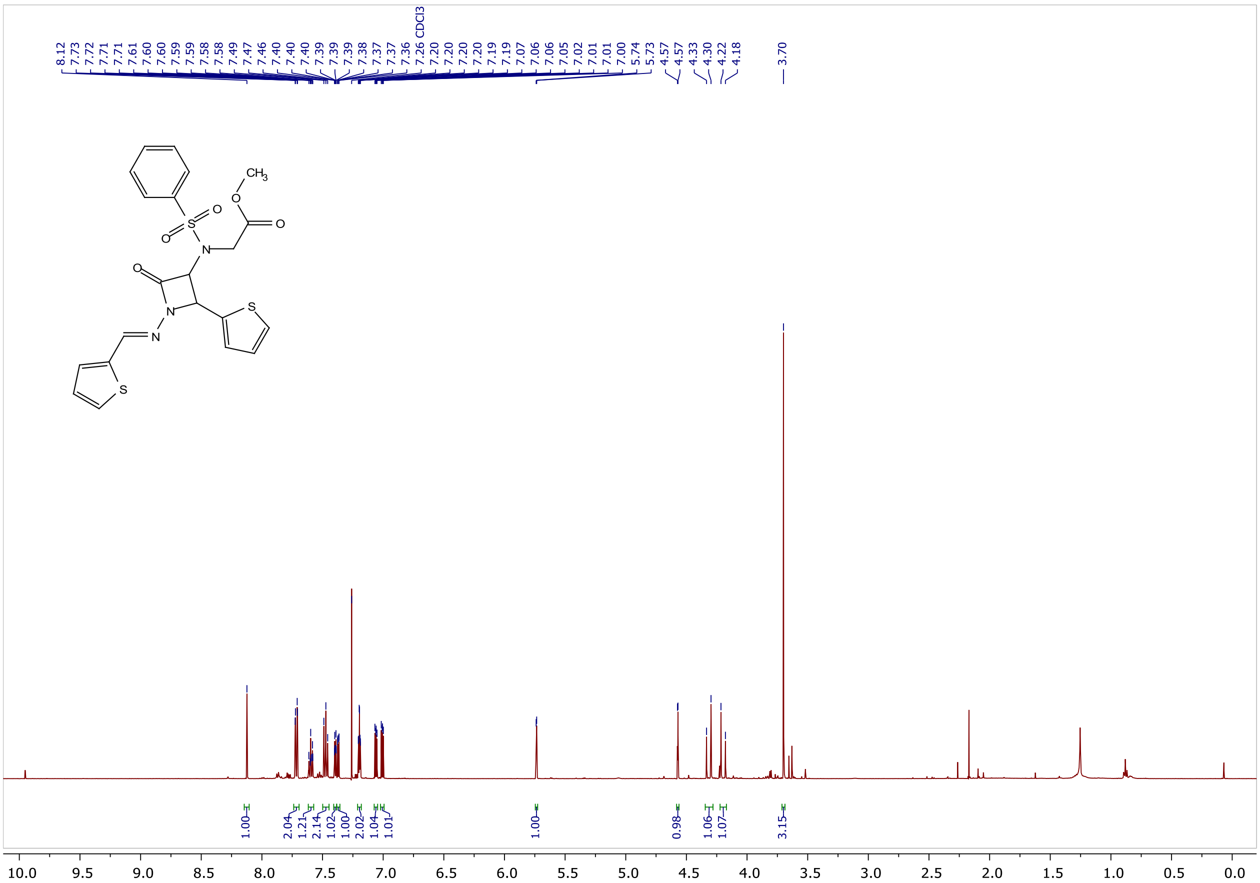

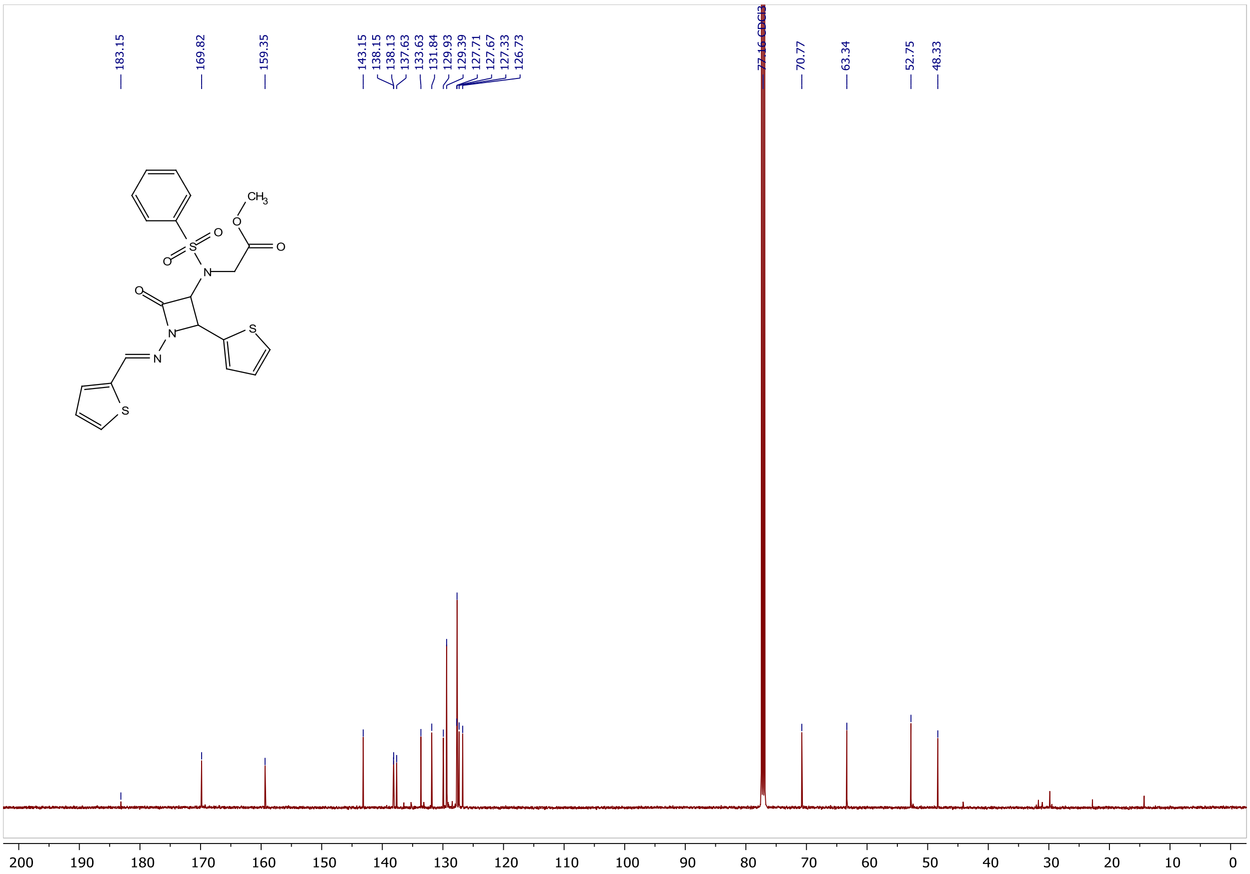

^{1}H and ^{13}C NMR spectra of compound **6n**

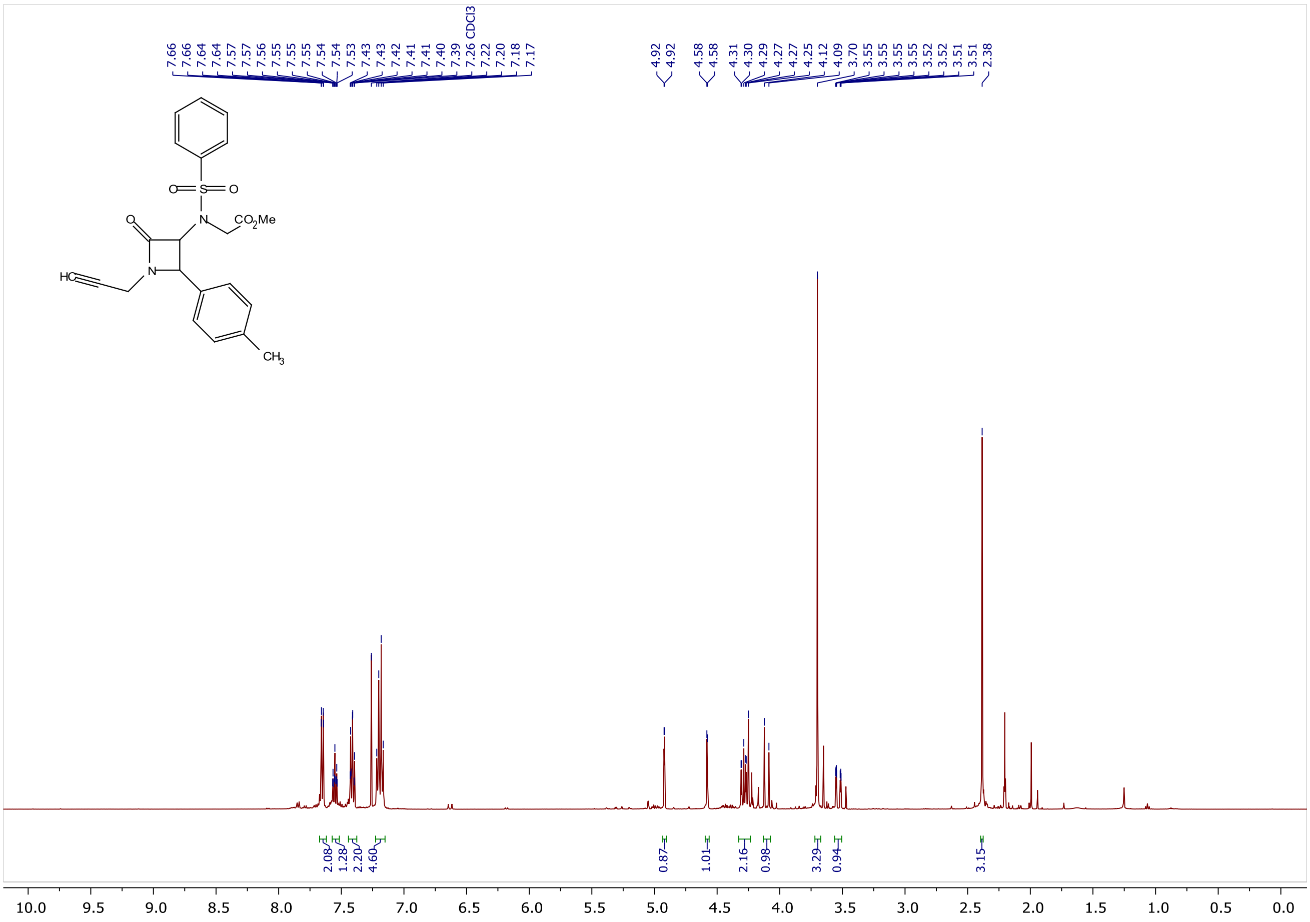

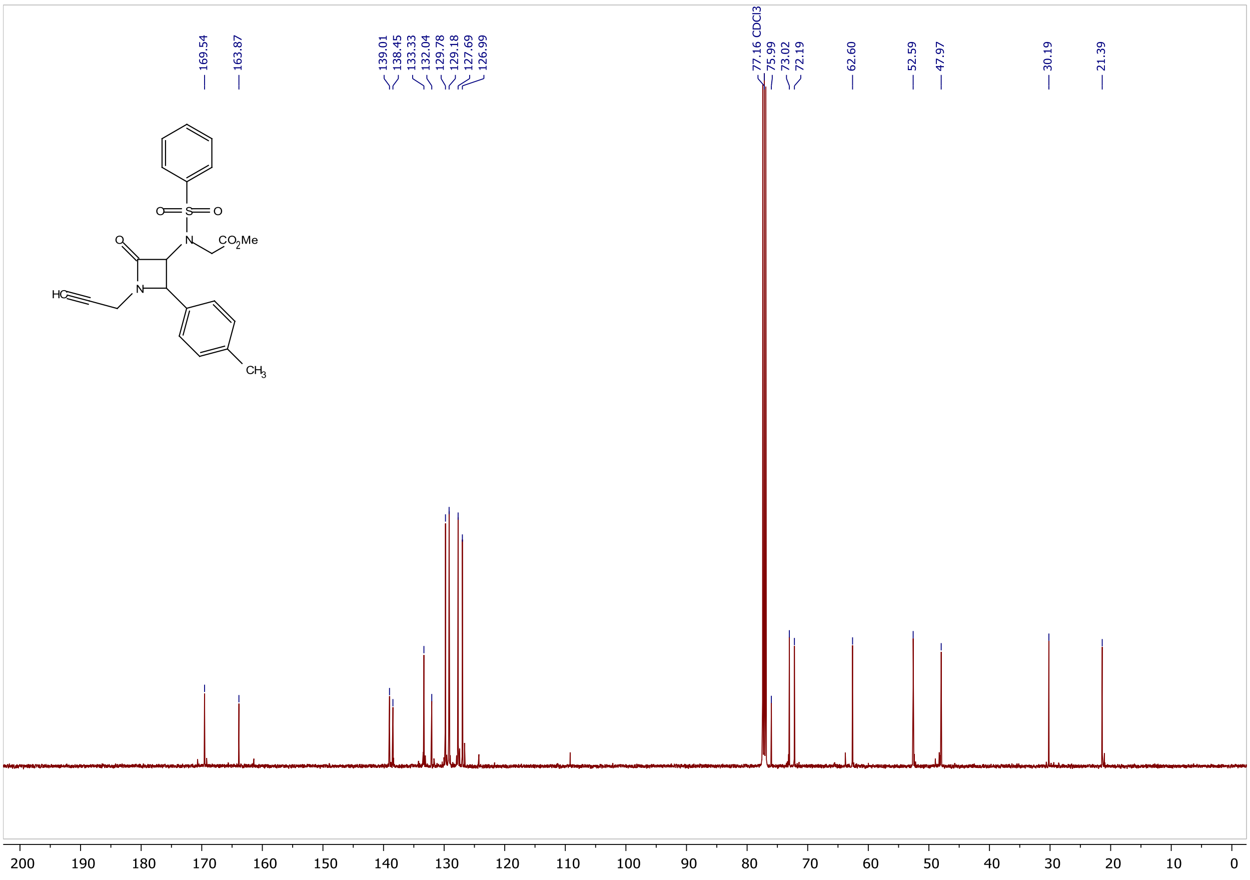

^{1}H and ^{13}C NMR spectra of compound **6o**

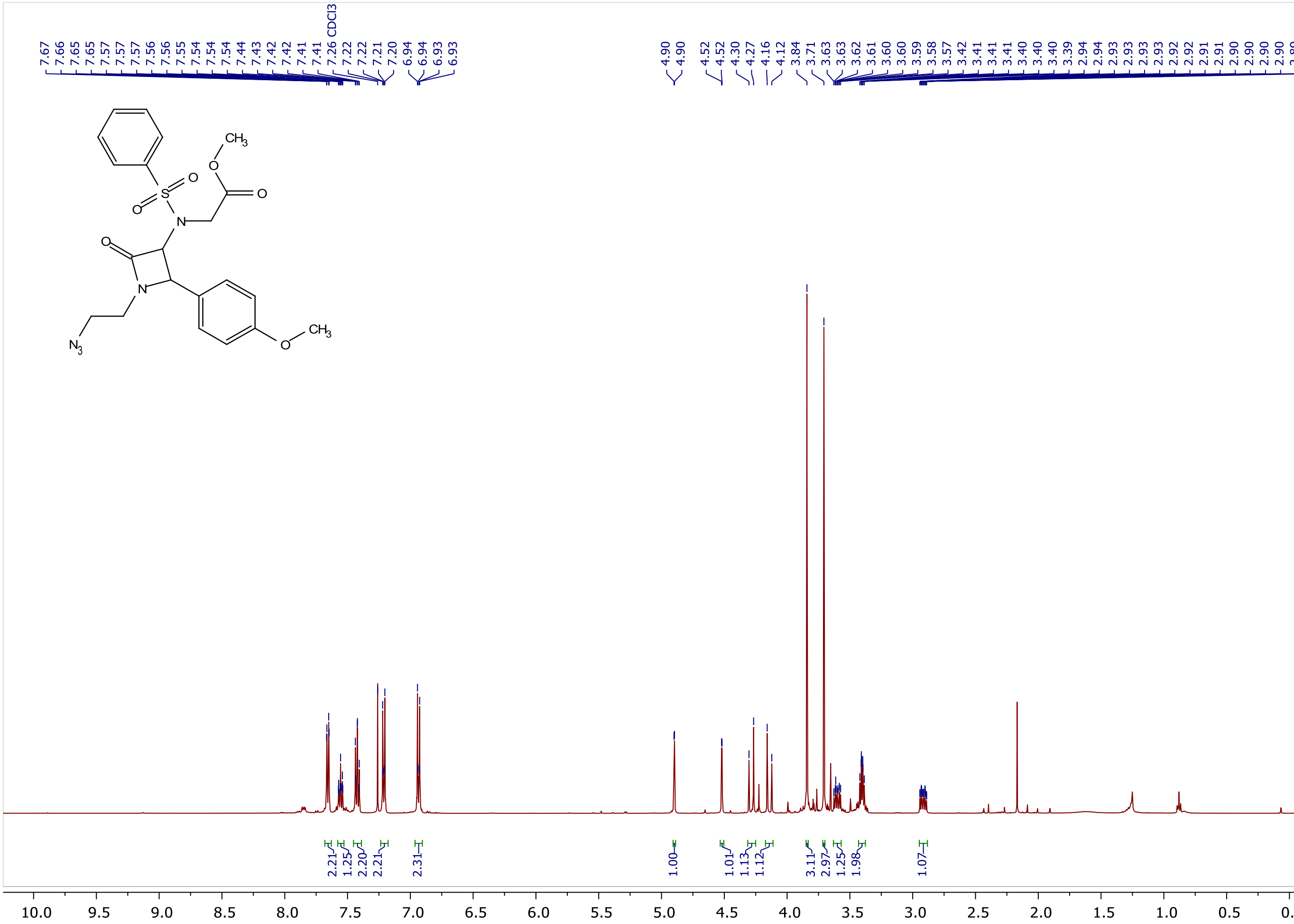

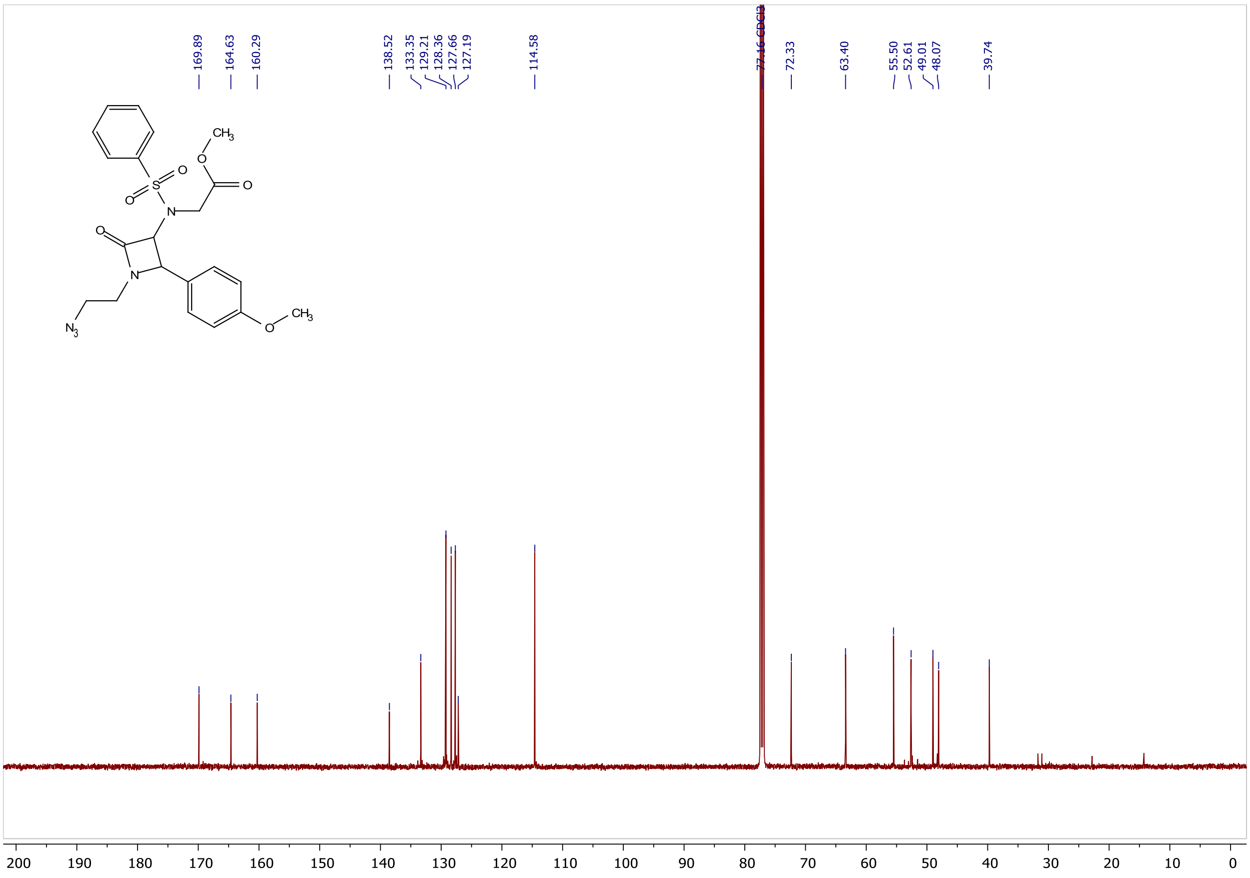

^{1}H and ^{13}C NMR spectra of compound **6p**

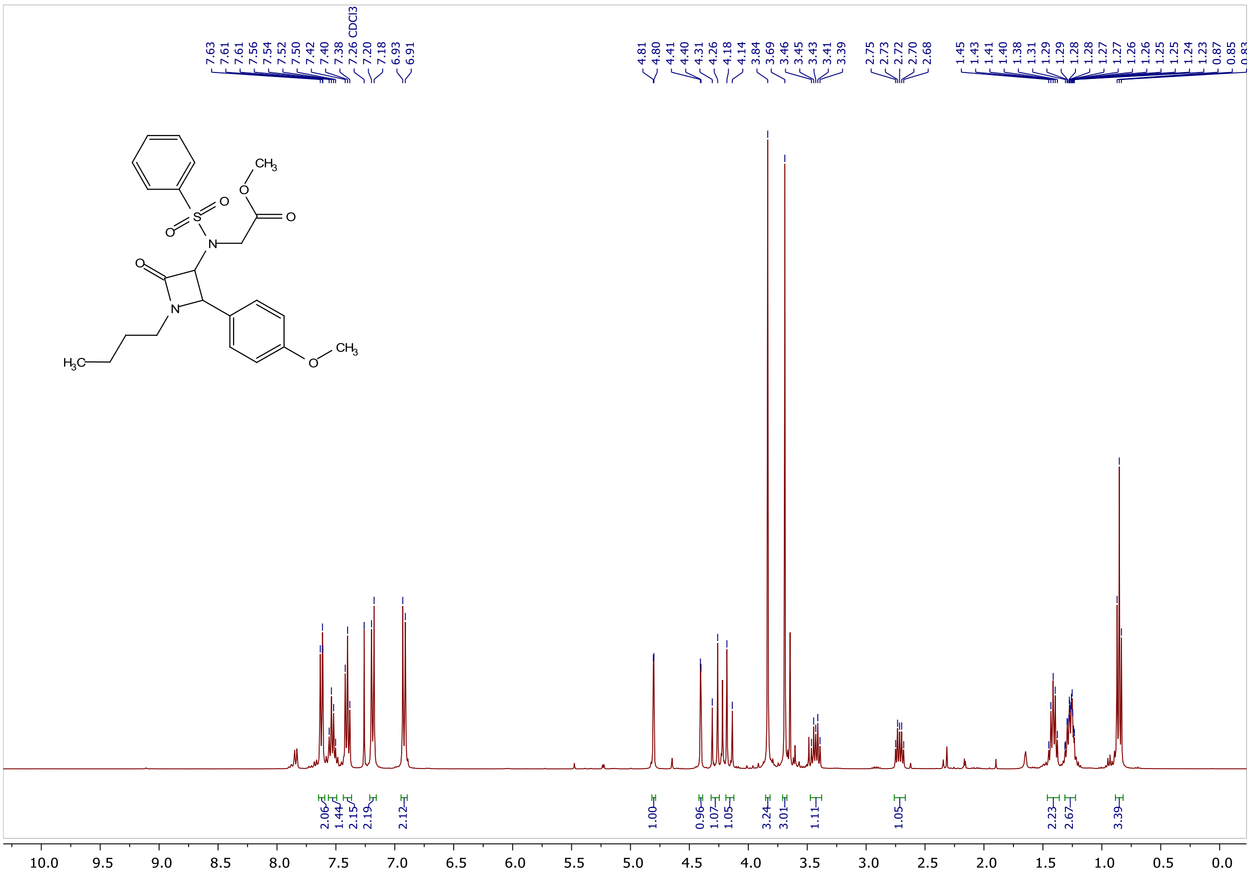

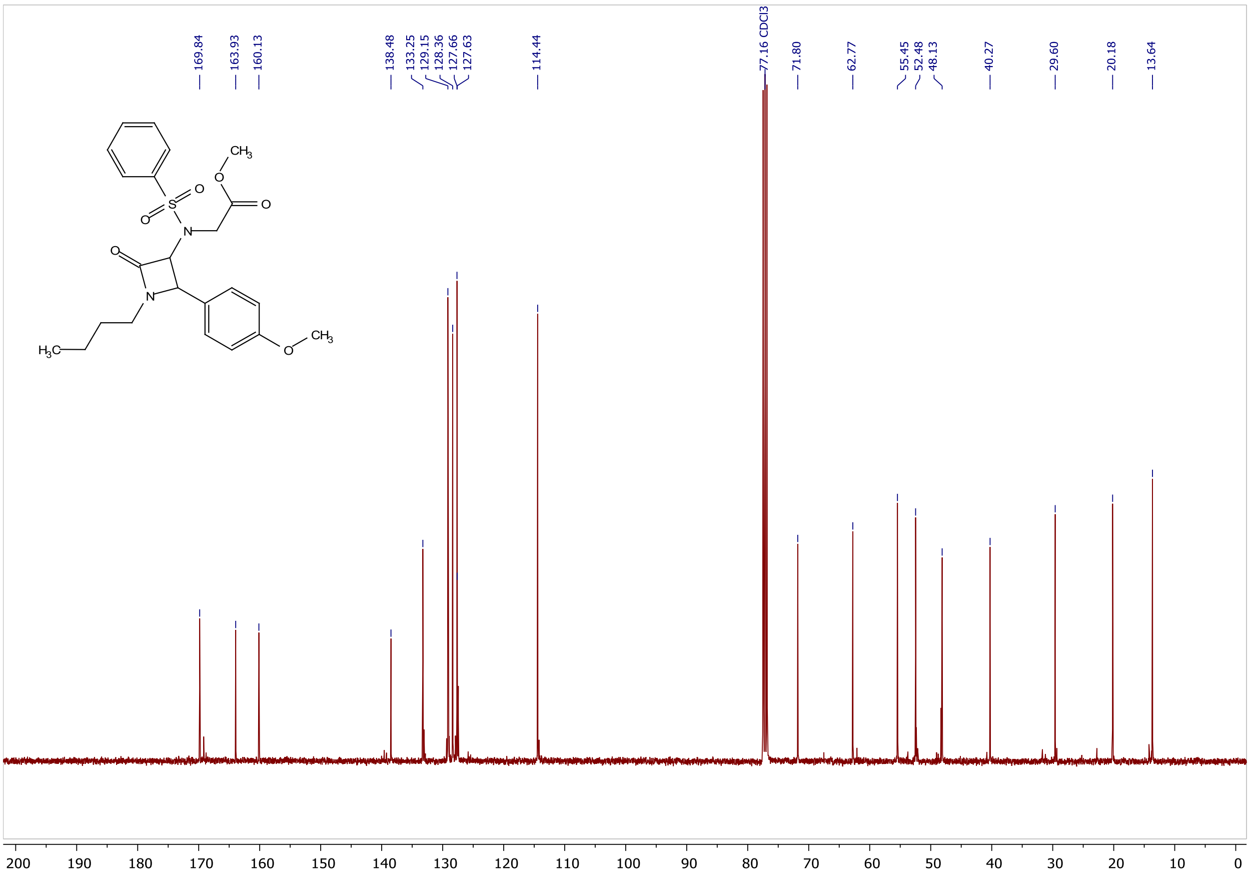

^{1}H and ^{13}C NMR spectra of compound **12a**

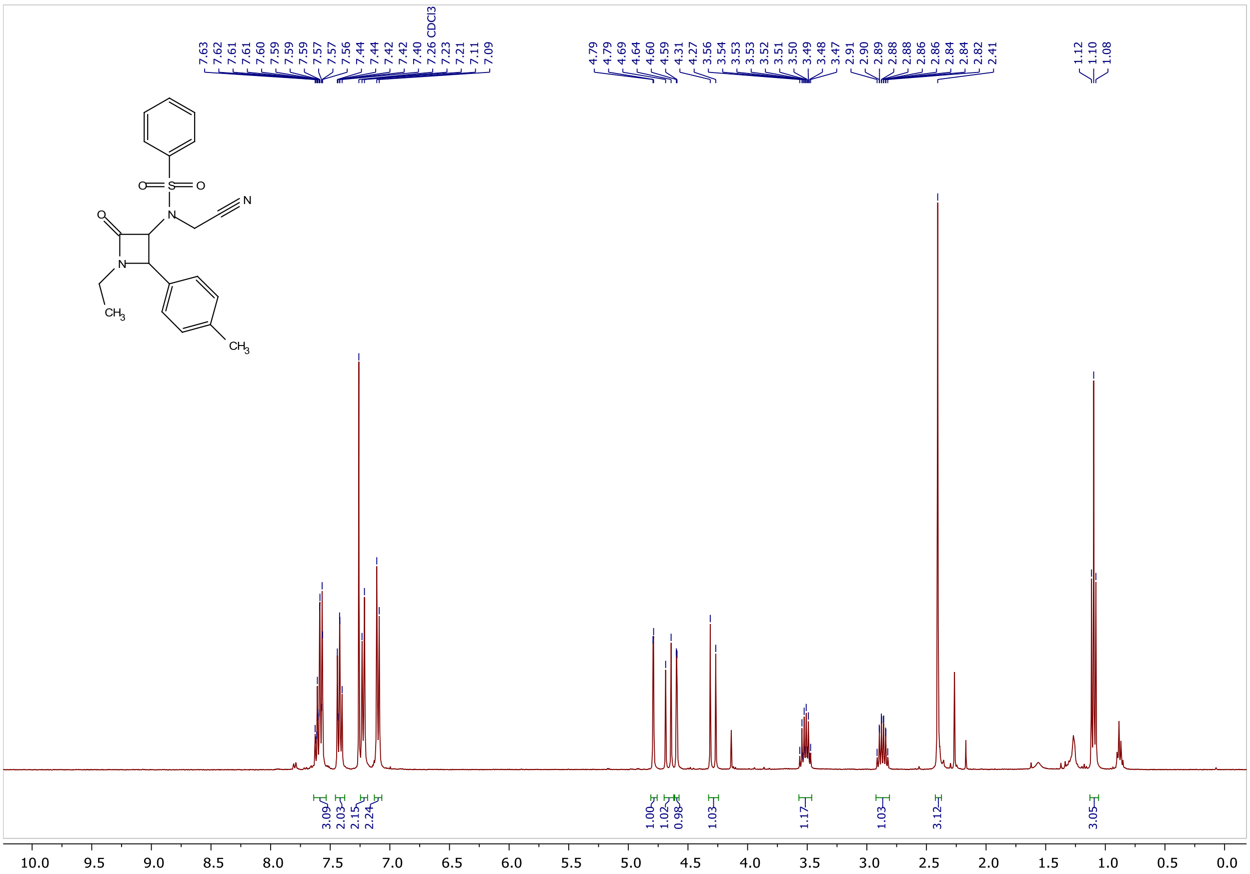

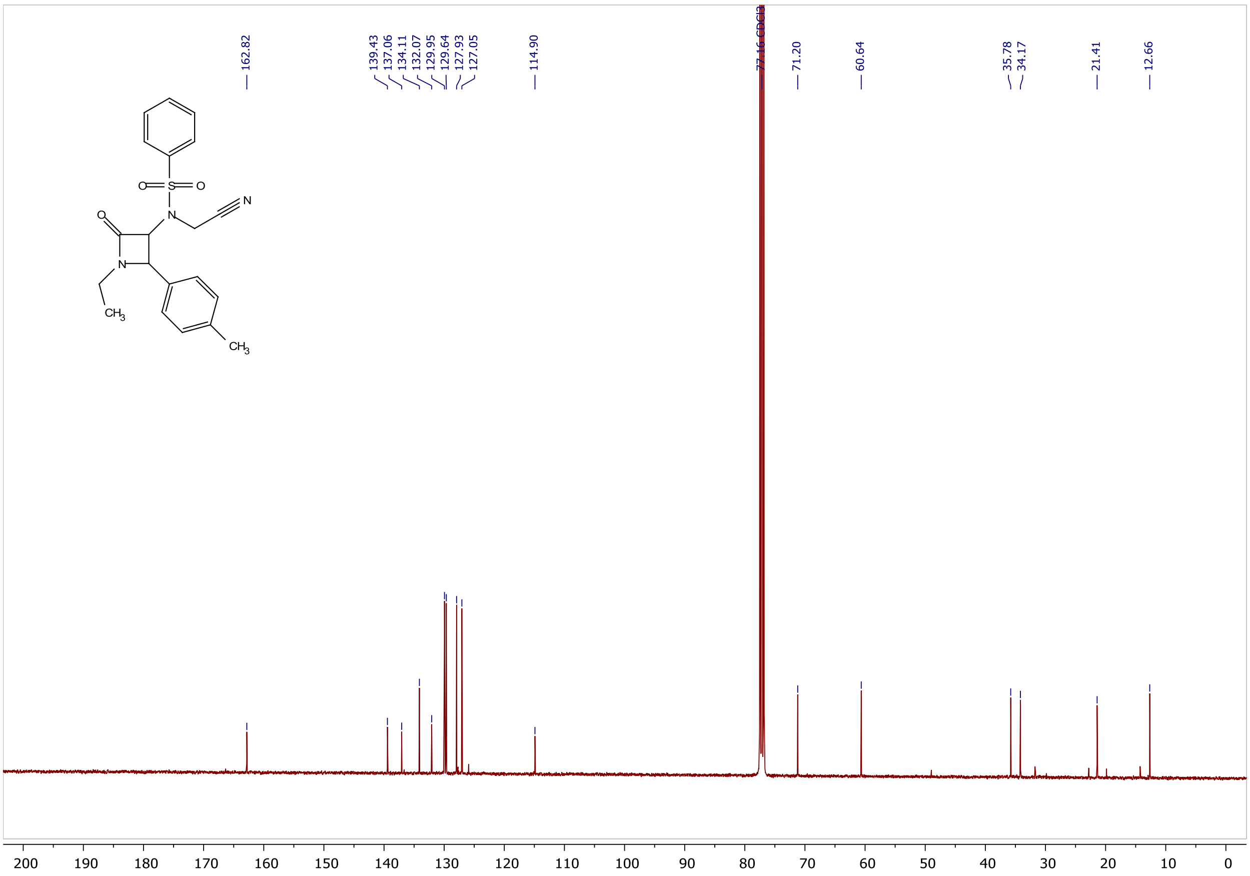

^{1}H, ^{13}C and ^{19}F NMR spectra of compound **12b**

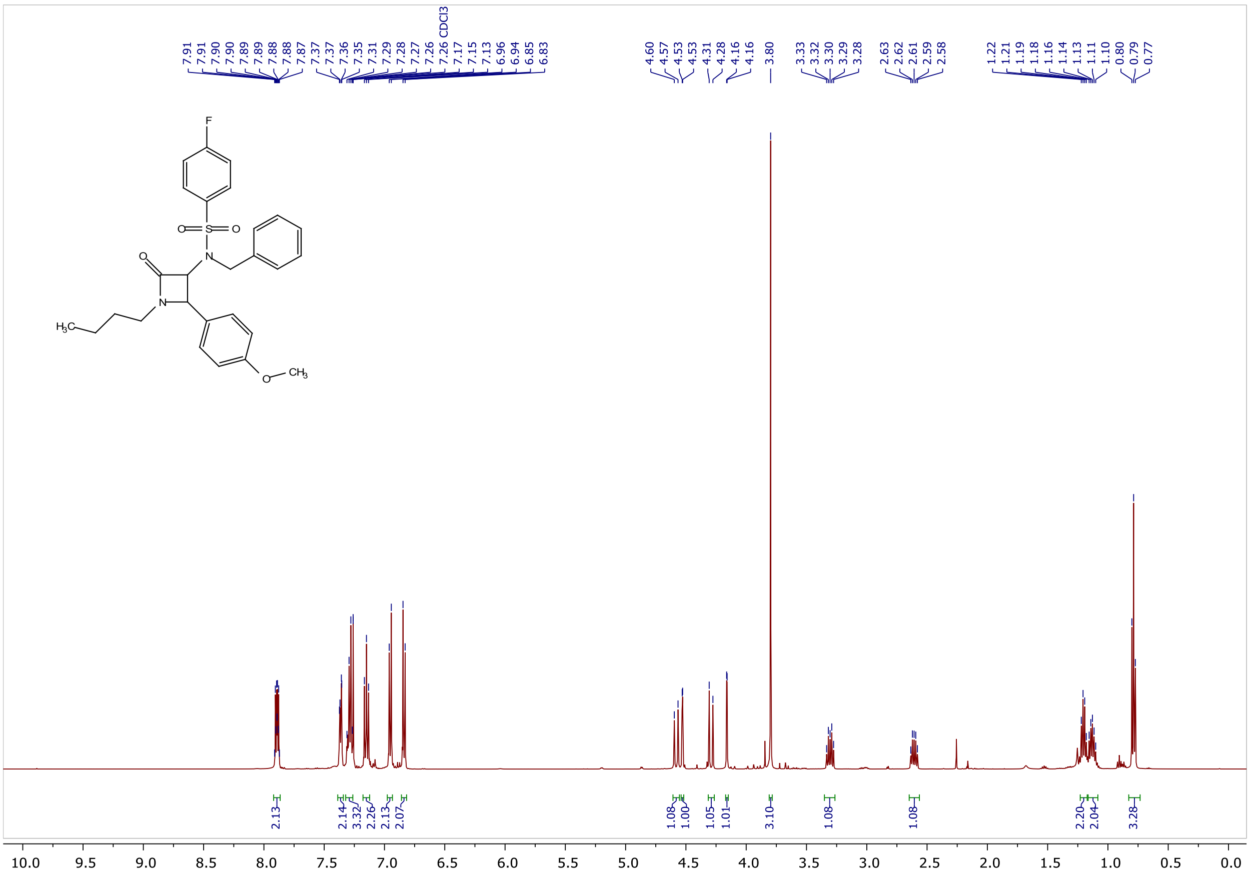

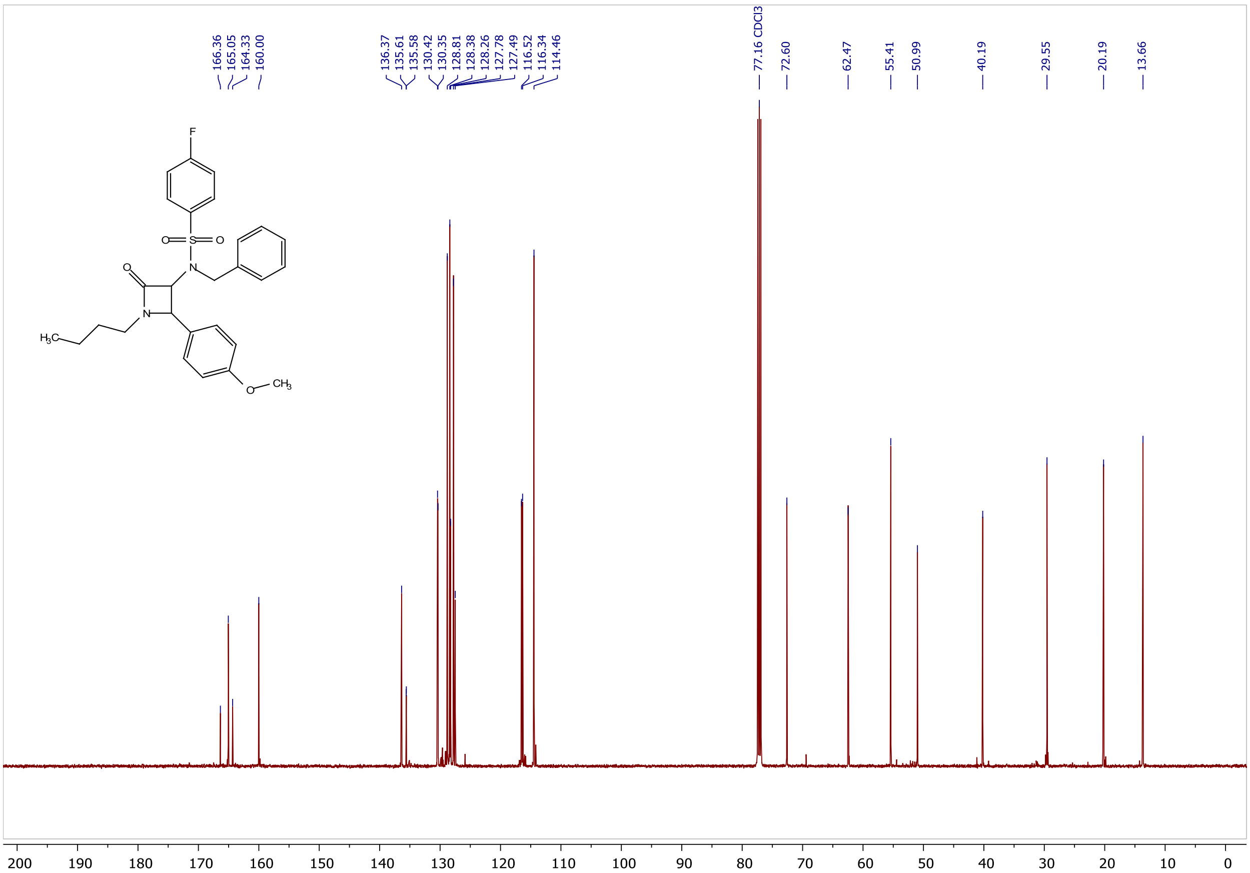

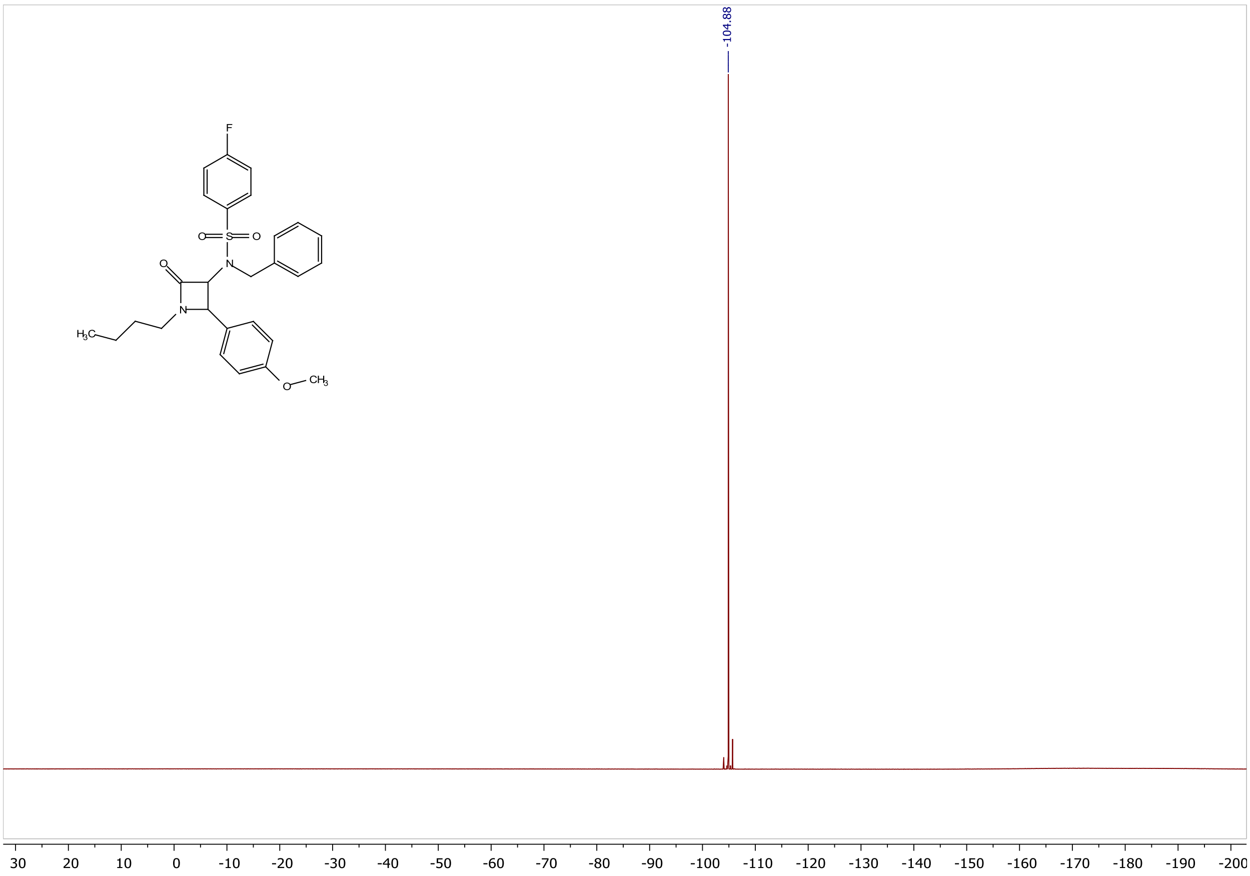

^{1}H and ^{13}C NMR spectra of compound **12c**

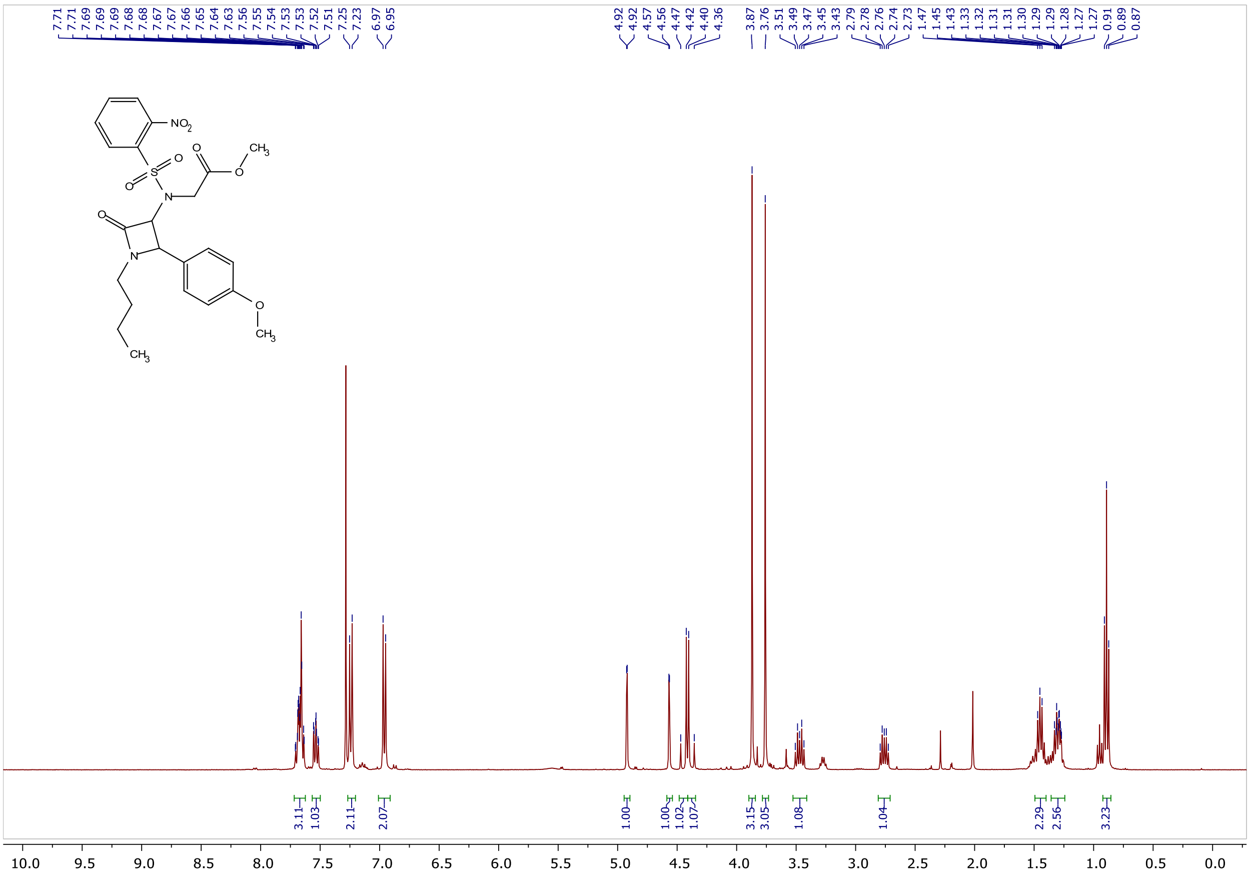

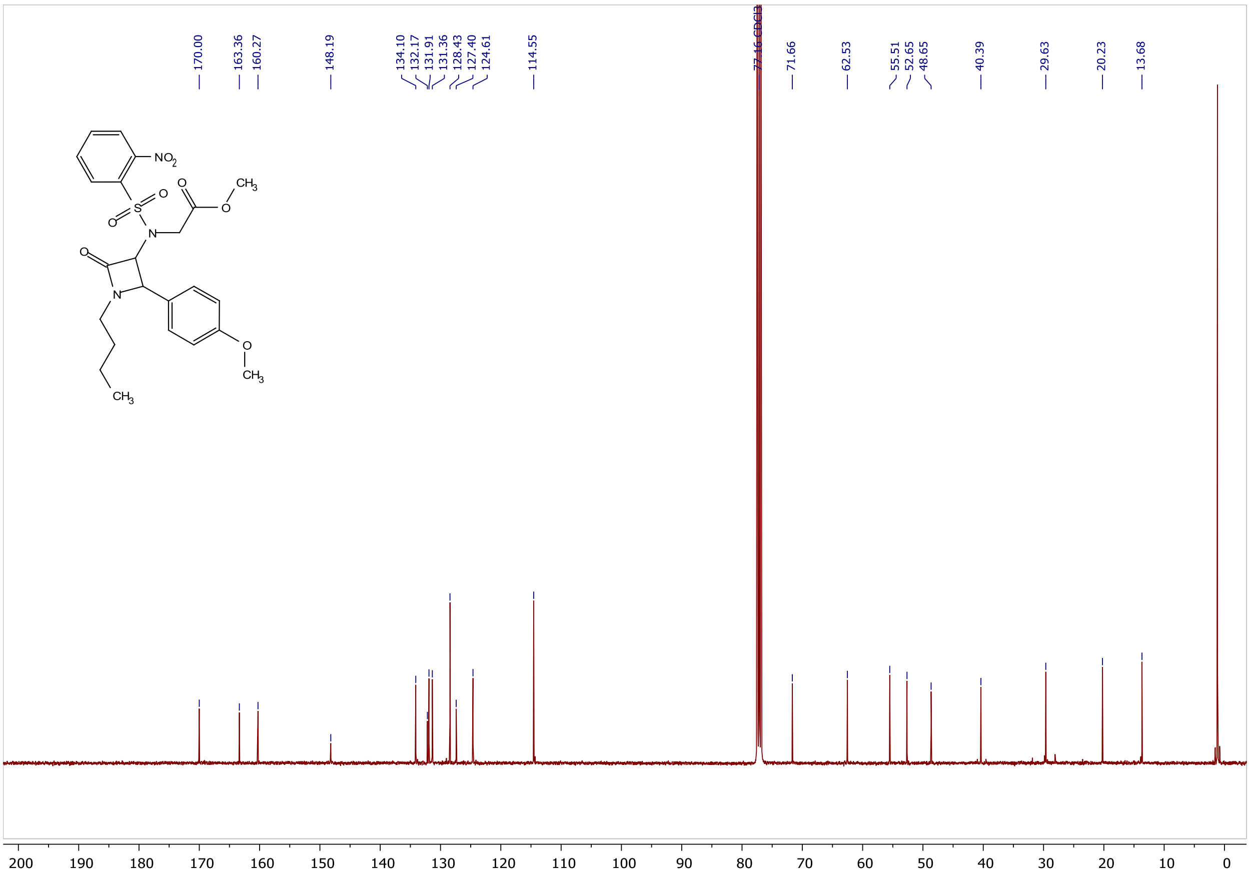

^{1}H and ^{13}C NMR spectra of compound **12d**

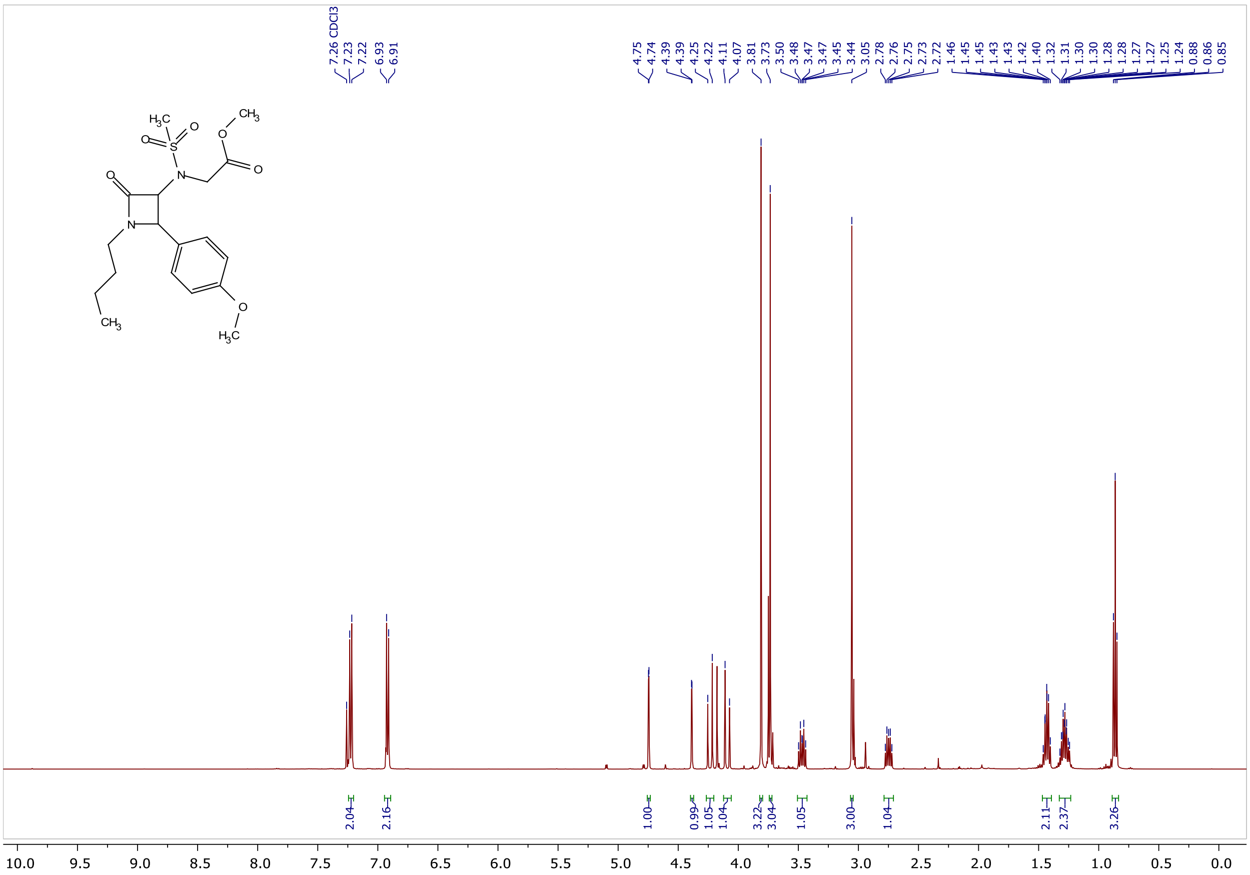

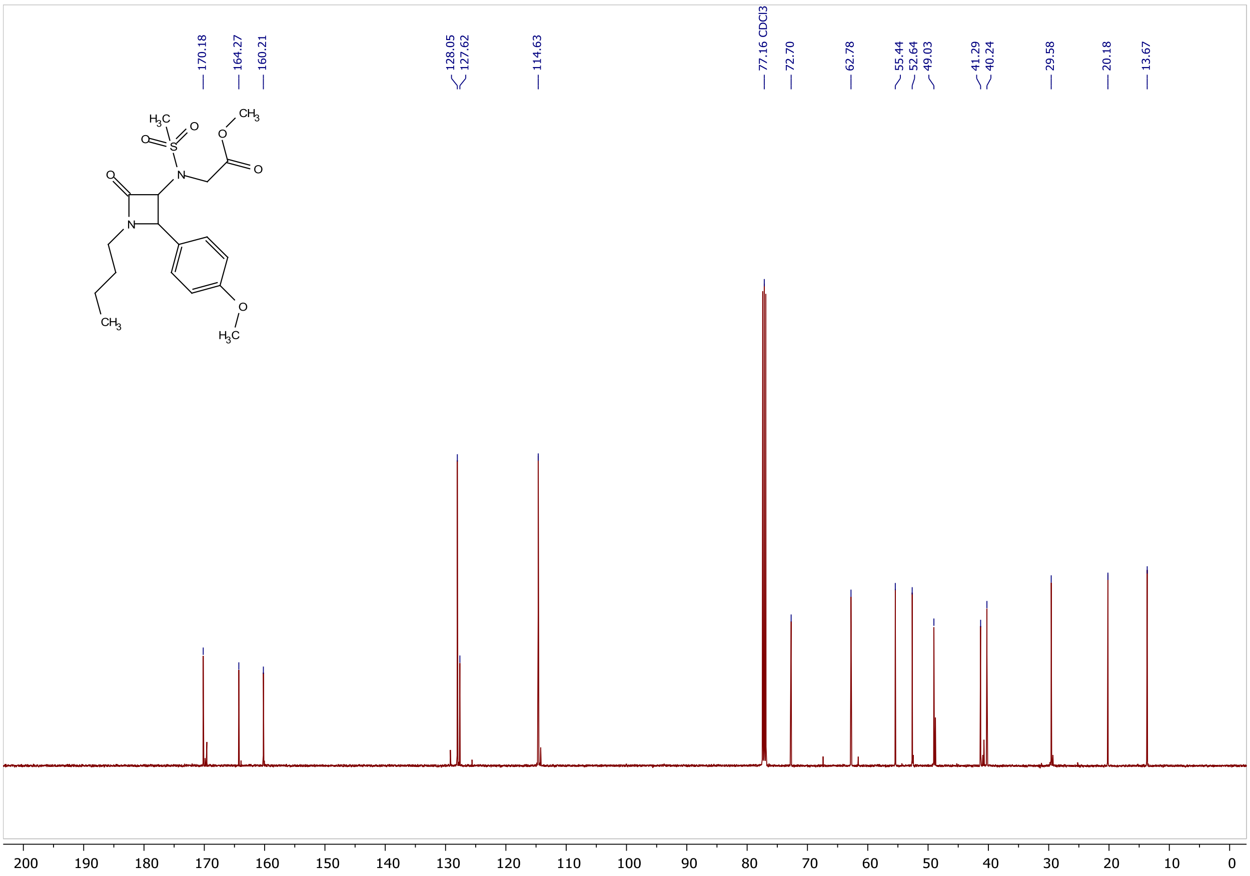

^{1}H and ^{13}C NMR spectra of compound **12e**

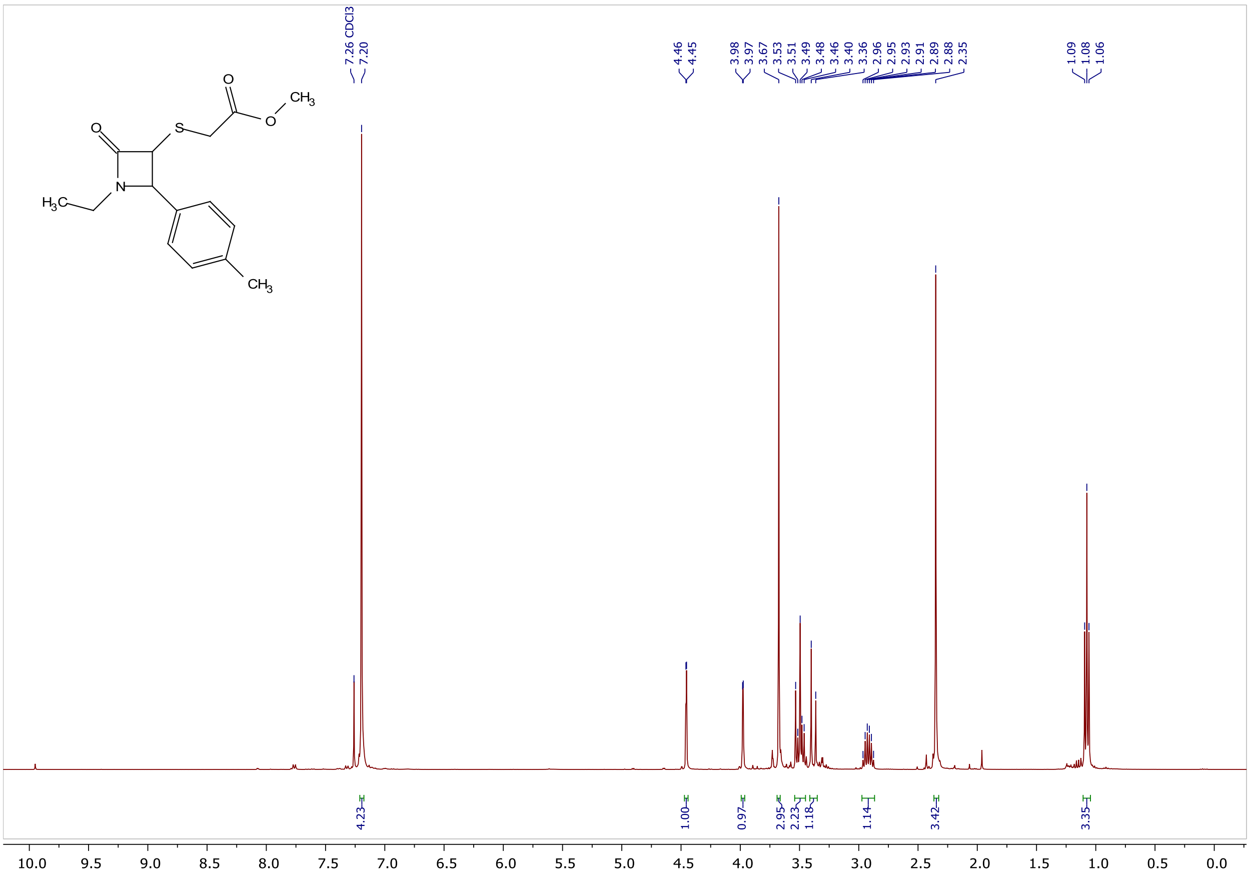

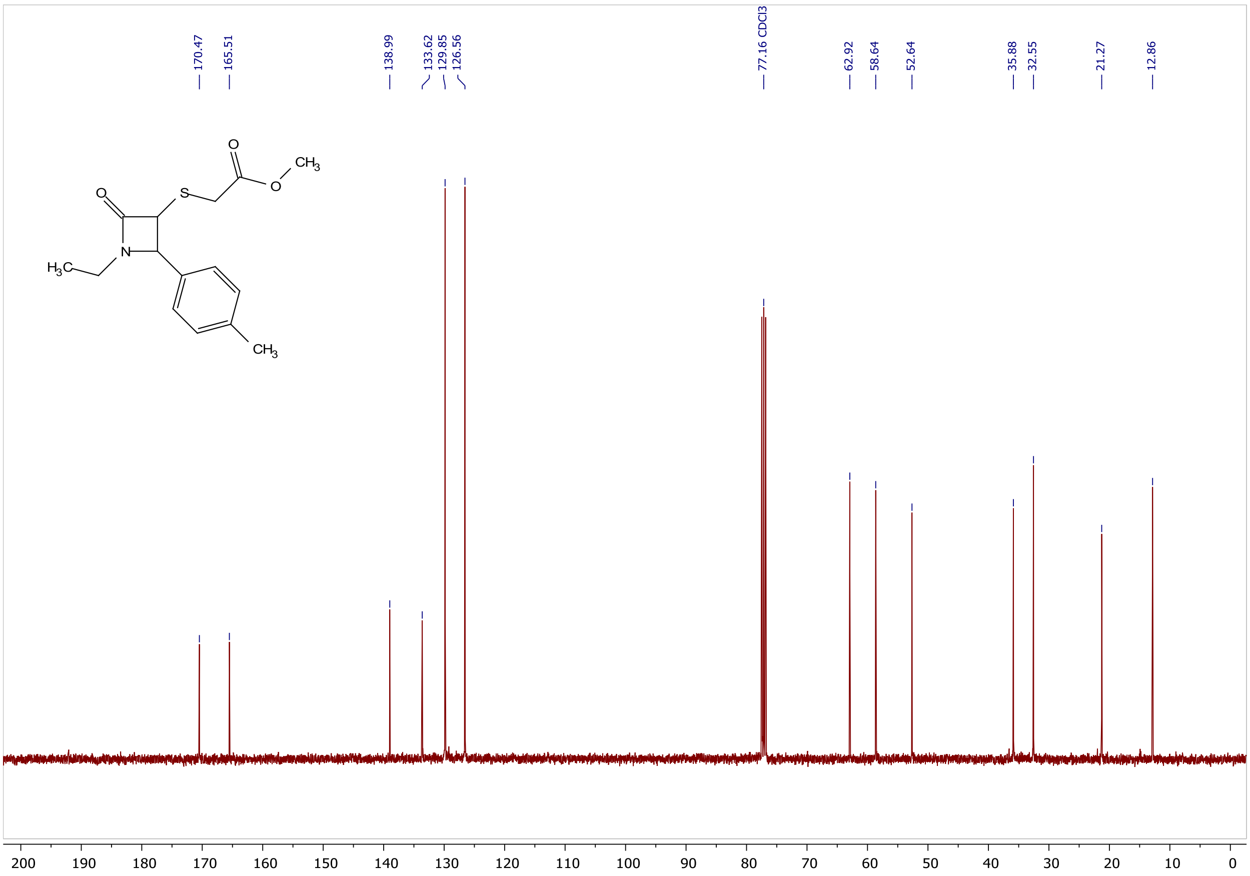

^{1}H and ^{13}C NMR spectra of compound **12f**

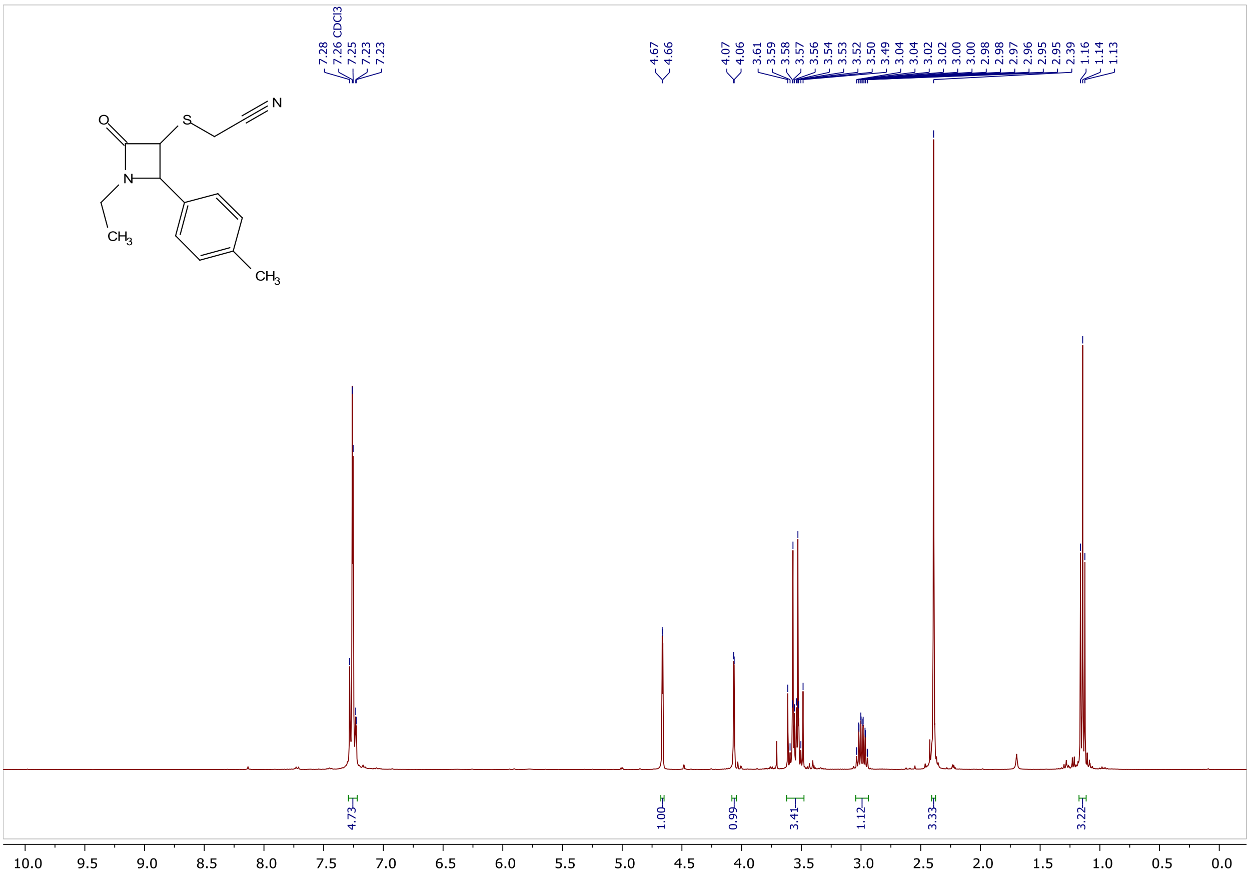

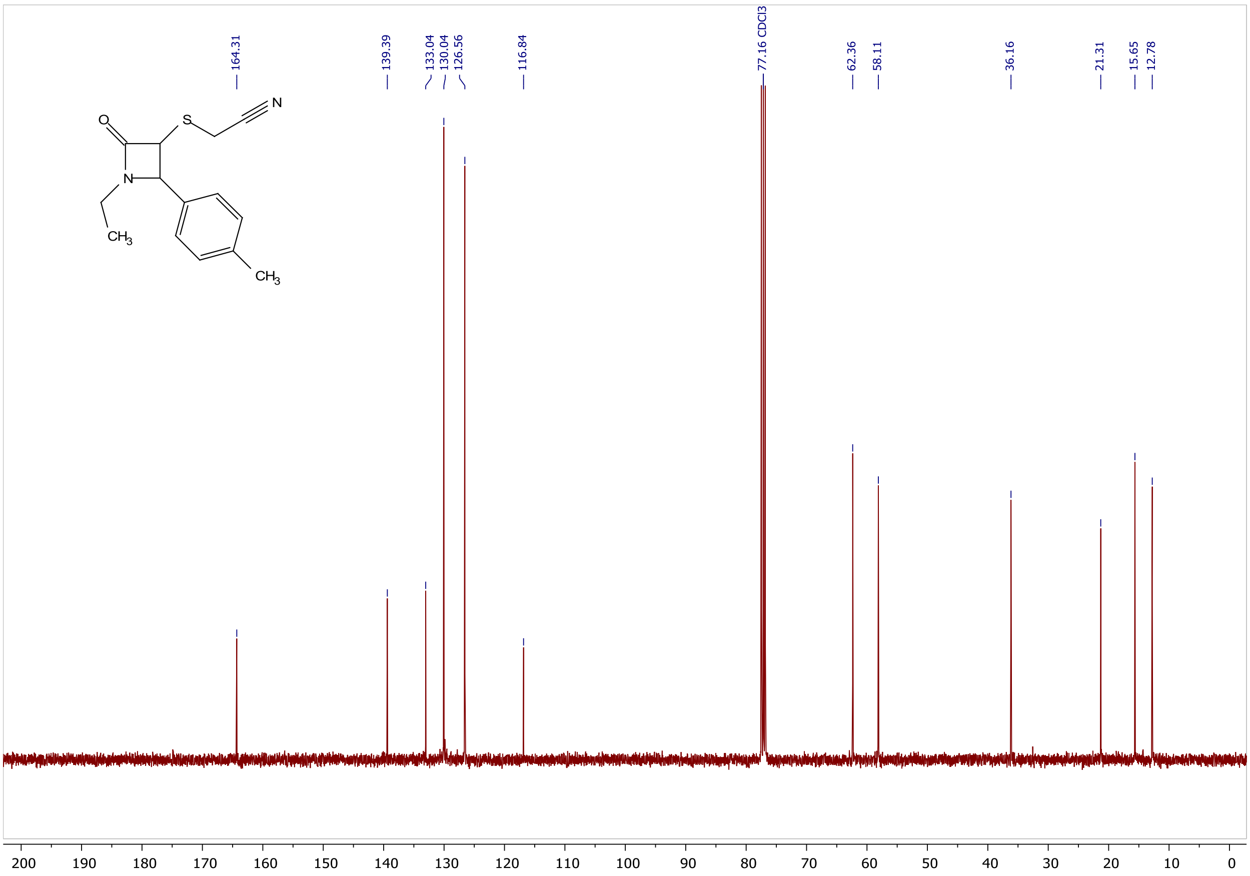

^{1}H, ^{13}C and ^{19}F NMR spectra of compound **12g**

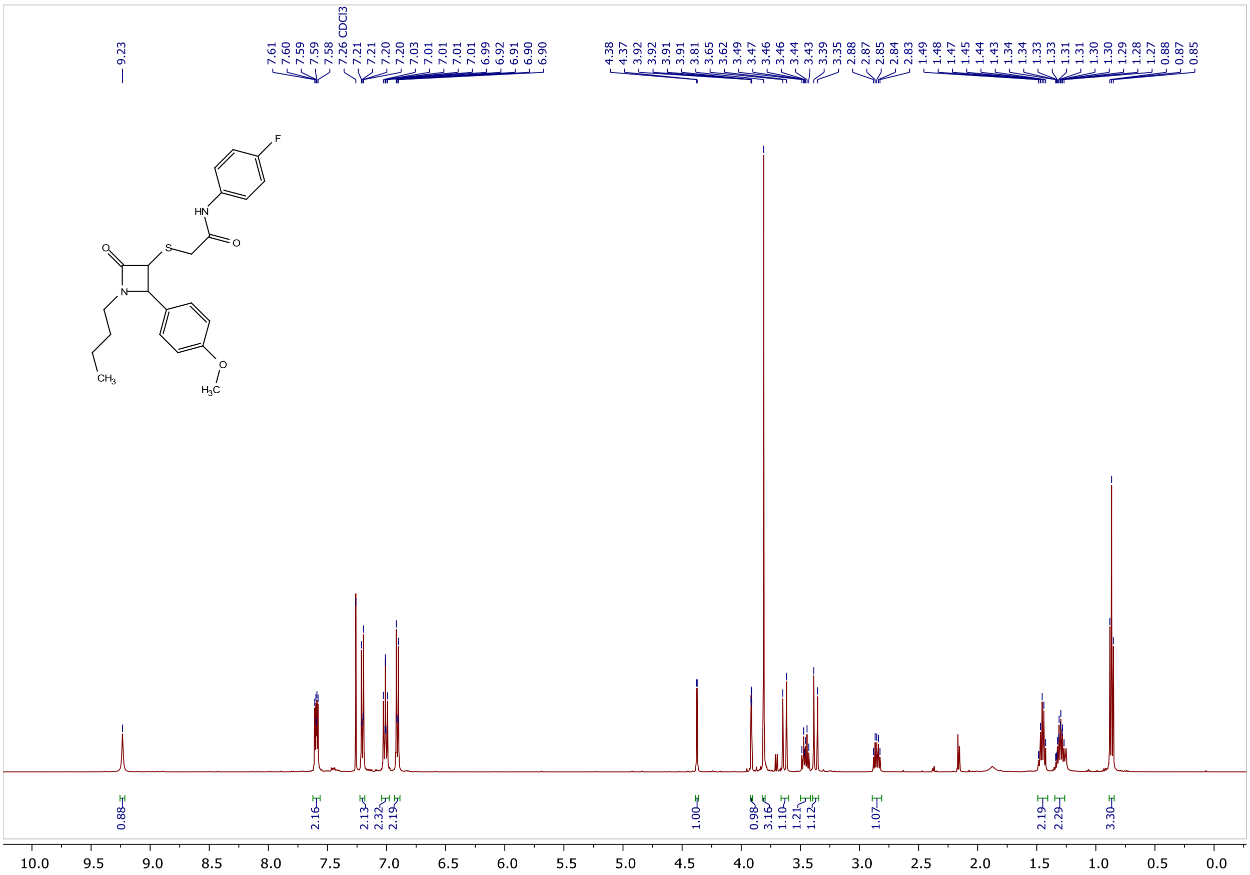

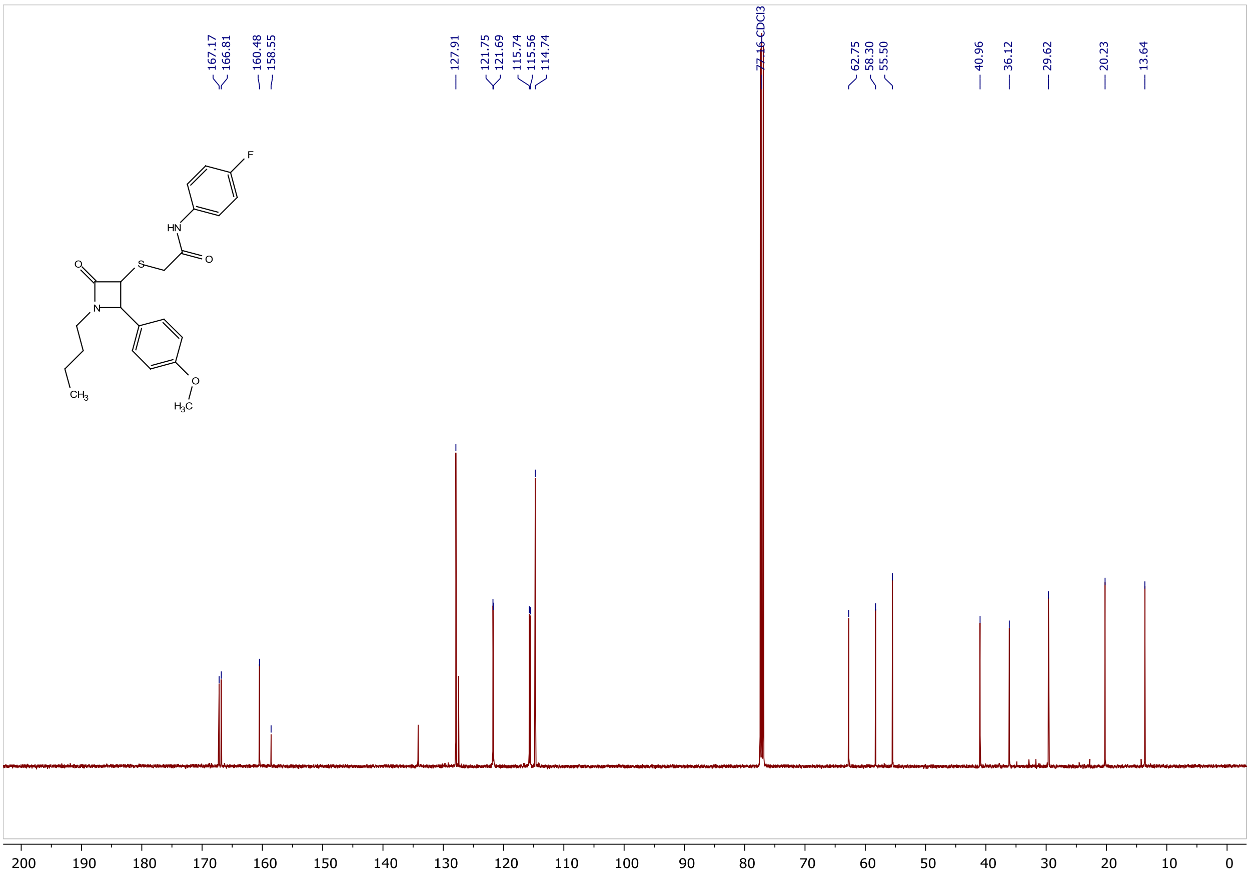
167.17
166.81
160.48
158.55
127.91
121.75
121.69
115.74
115.56
114.74
77.16 CDCl3
62.75
58.30
55.50
40.96
36.12
29.62
20.23
13.64
F
HN
O
S
O
N
CH3
O
H3C
200 190 180 170 160 150 140 130 120 110 100 90 80 70 60 50 40 30 20 10 0

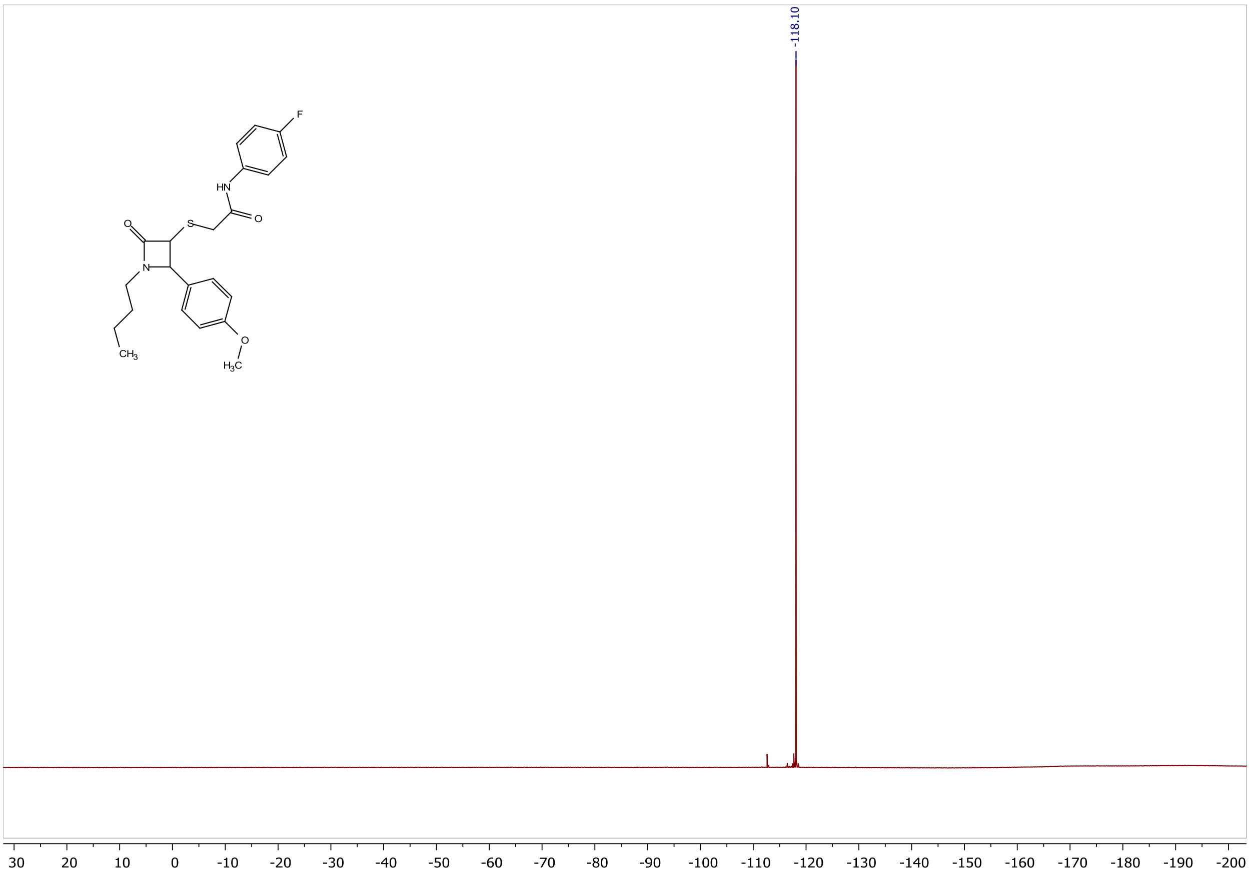
-118.10
F
HN
O
S
O
N
CH3
O
H3C
30 20 10 0 -10 -20 -30 -40 -50 -60 -70 -80 -90 -100 -110 -120 -130 -140 -150 -160 -170 -180 -190 -200

^{1}H and ^{13}C NMR spectra of compound **12m**

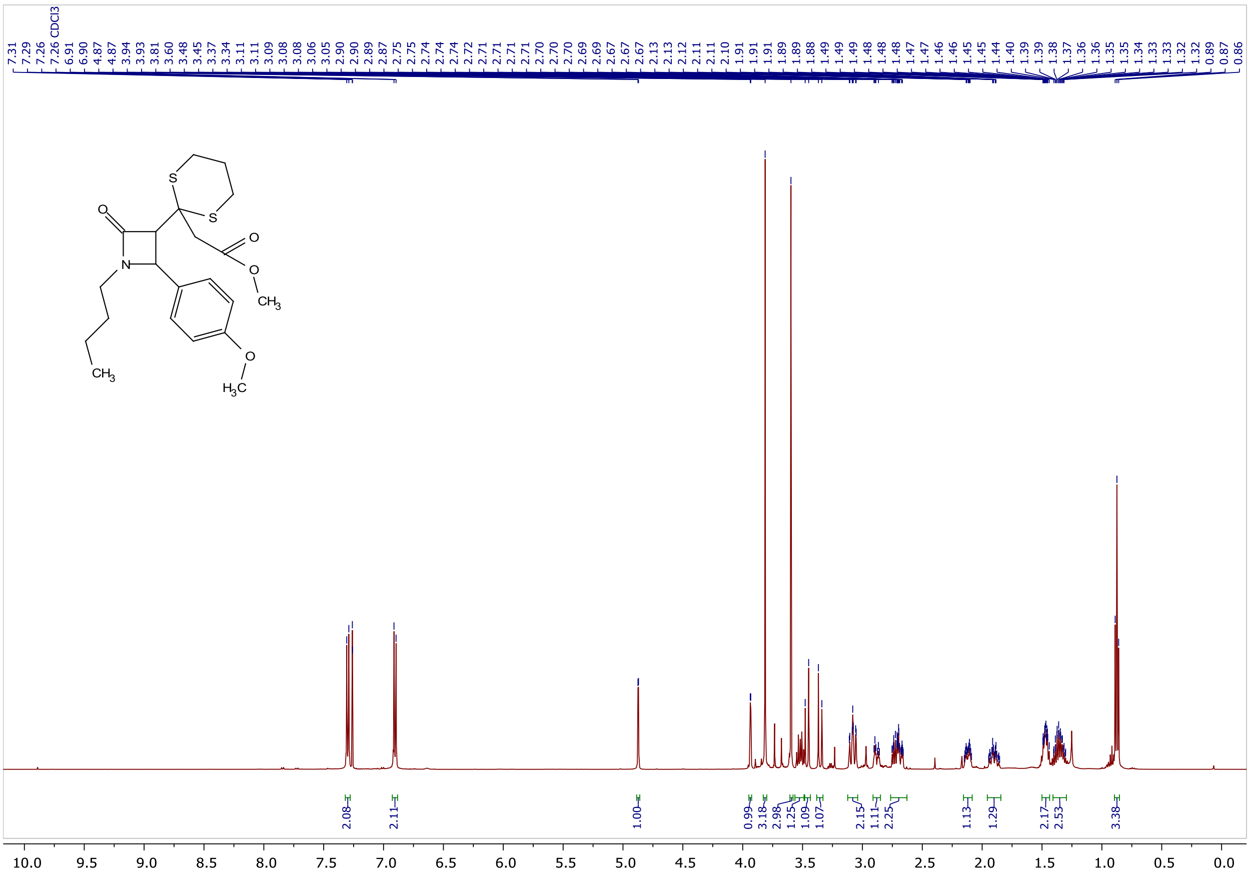

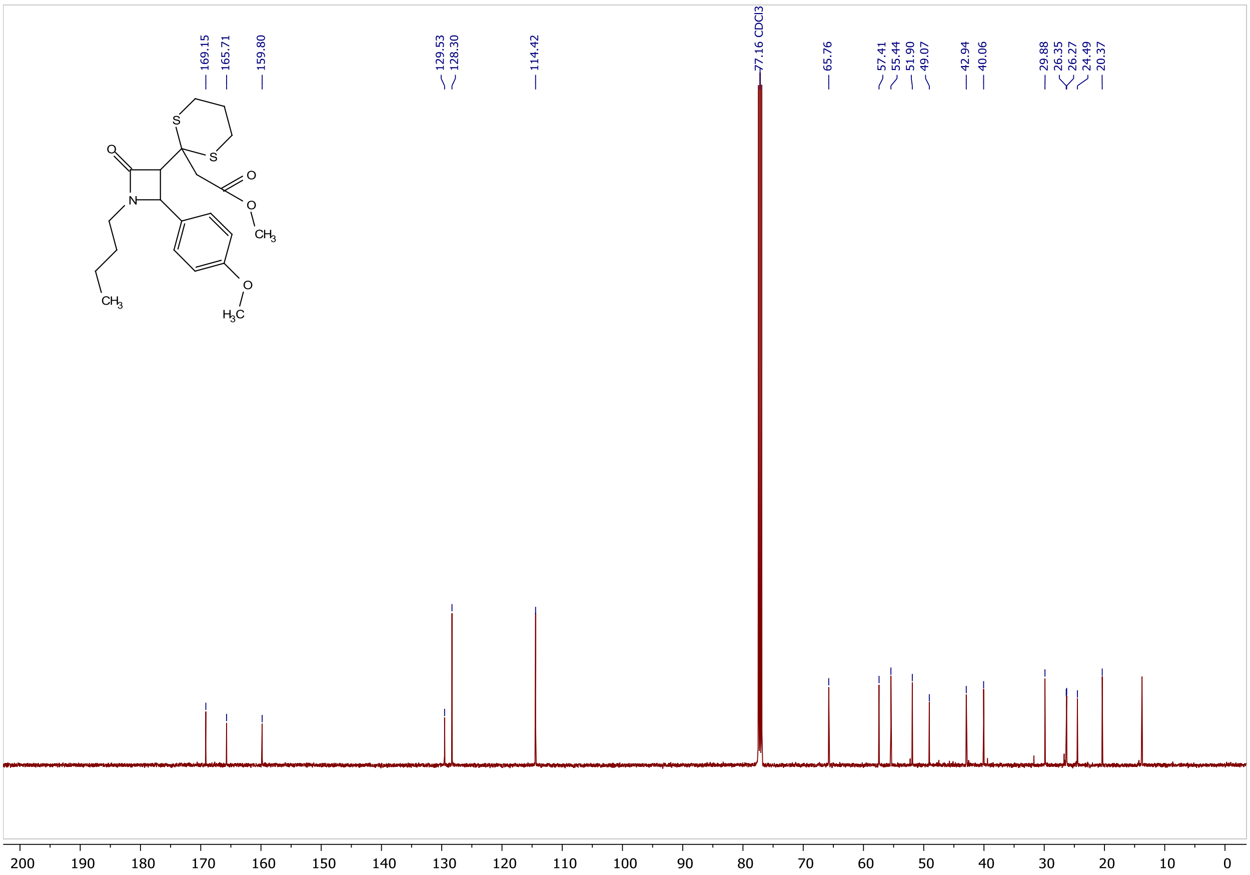

^{1}H, ^{13}C and ^{19}F NMR spectra of compound **16**

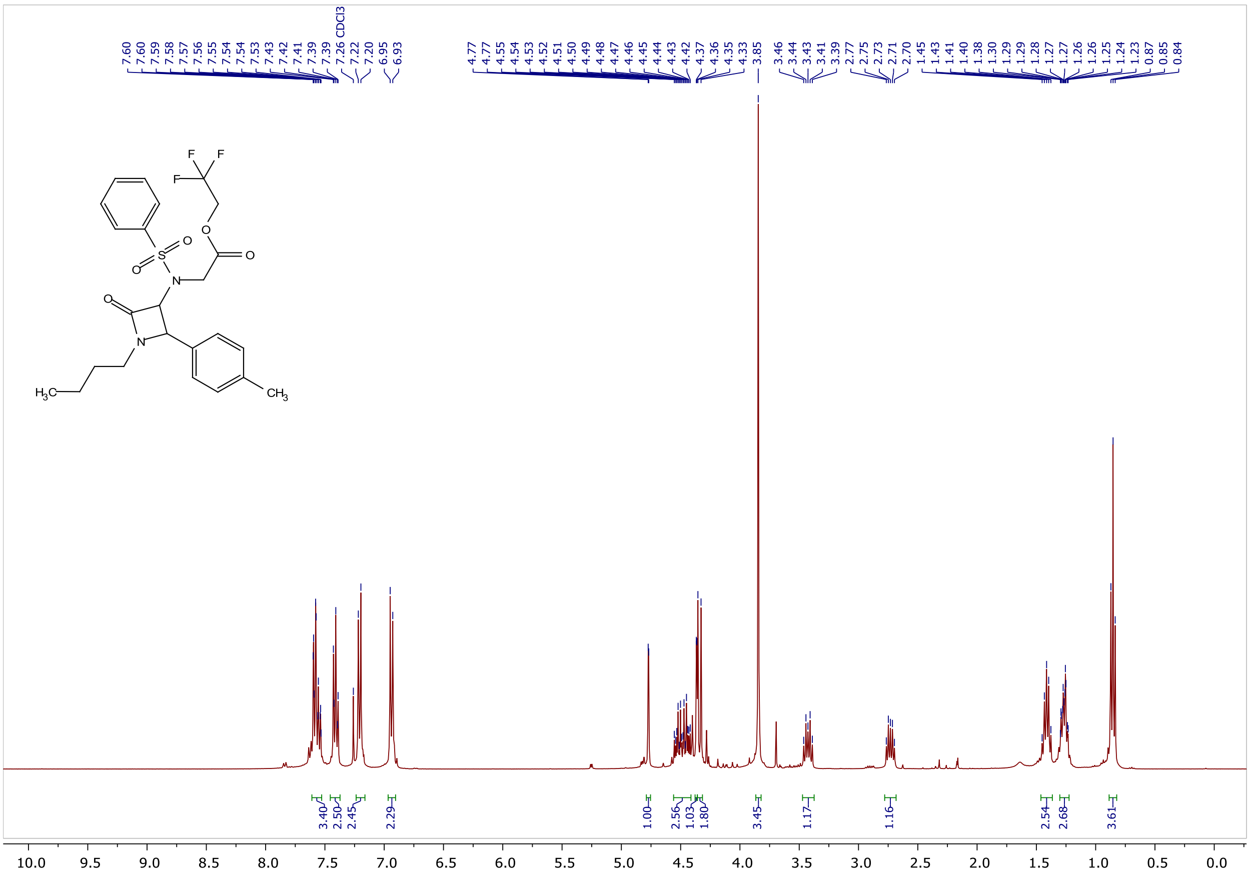

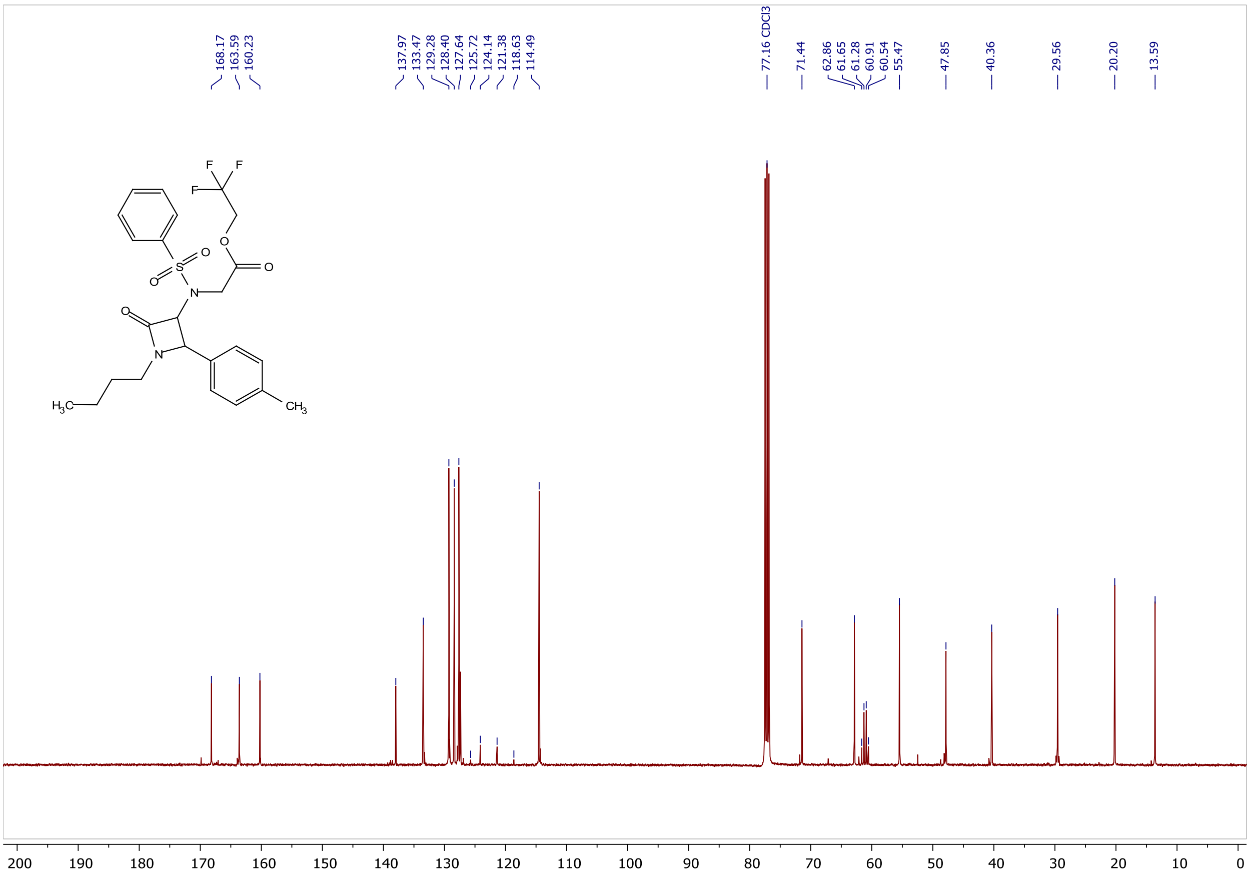

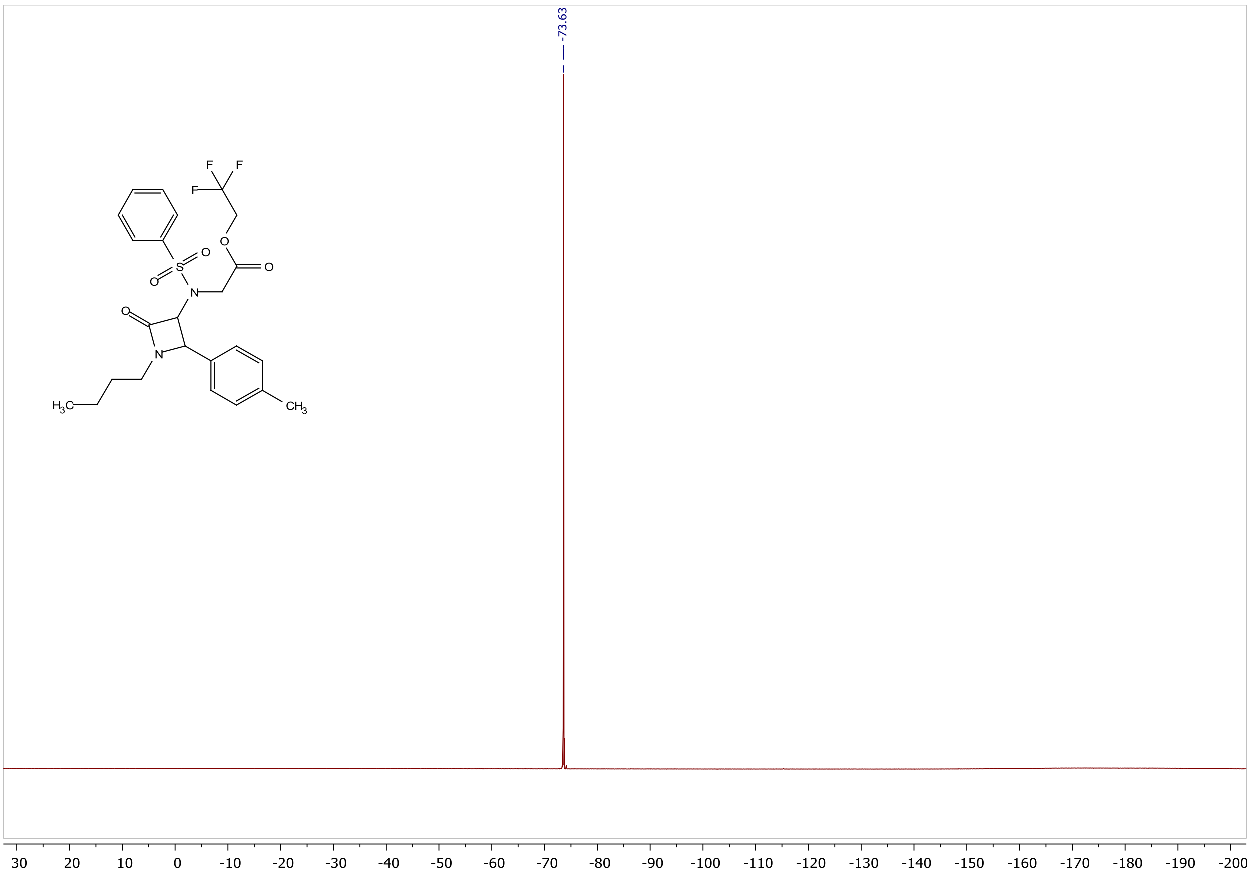

^{1}H and ^{13}C NMR spectra of compound **18**

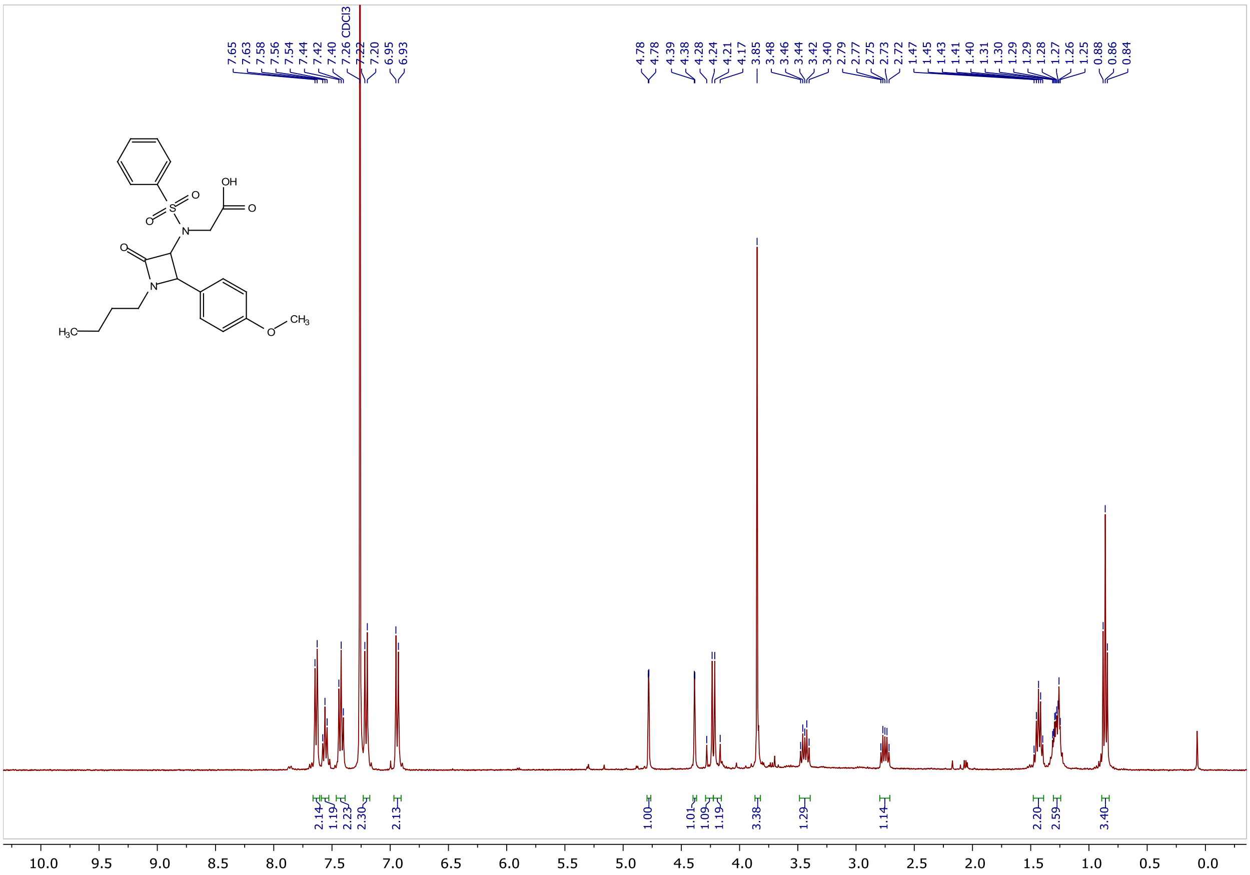

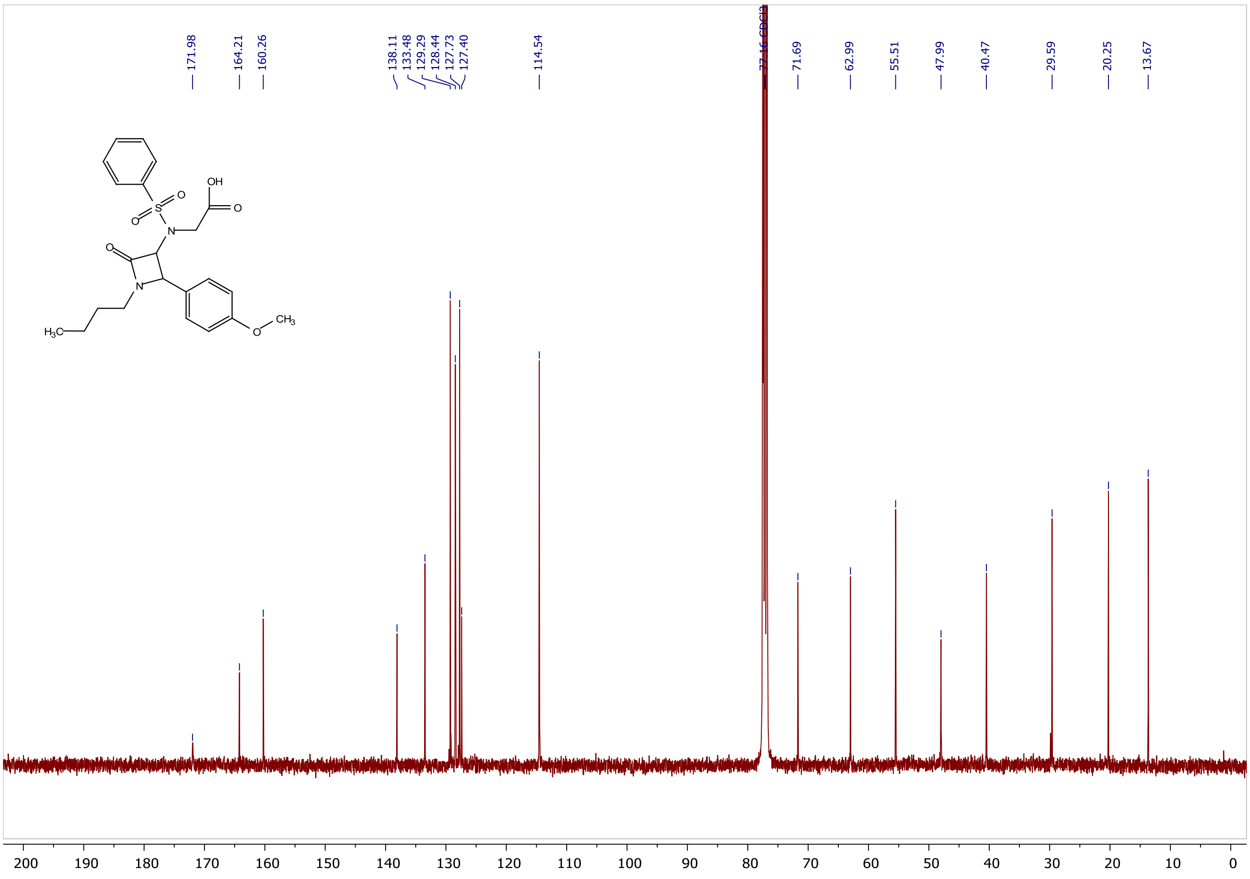

^{1}H and ^{13}C NMR spectra of compound **19**

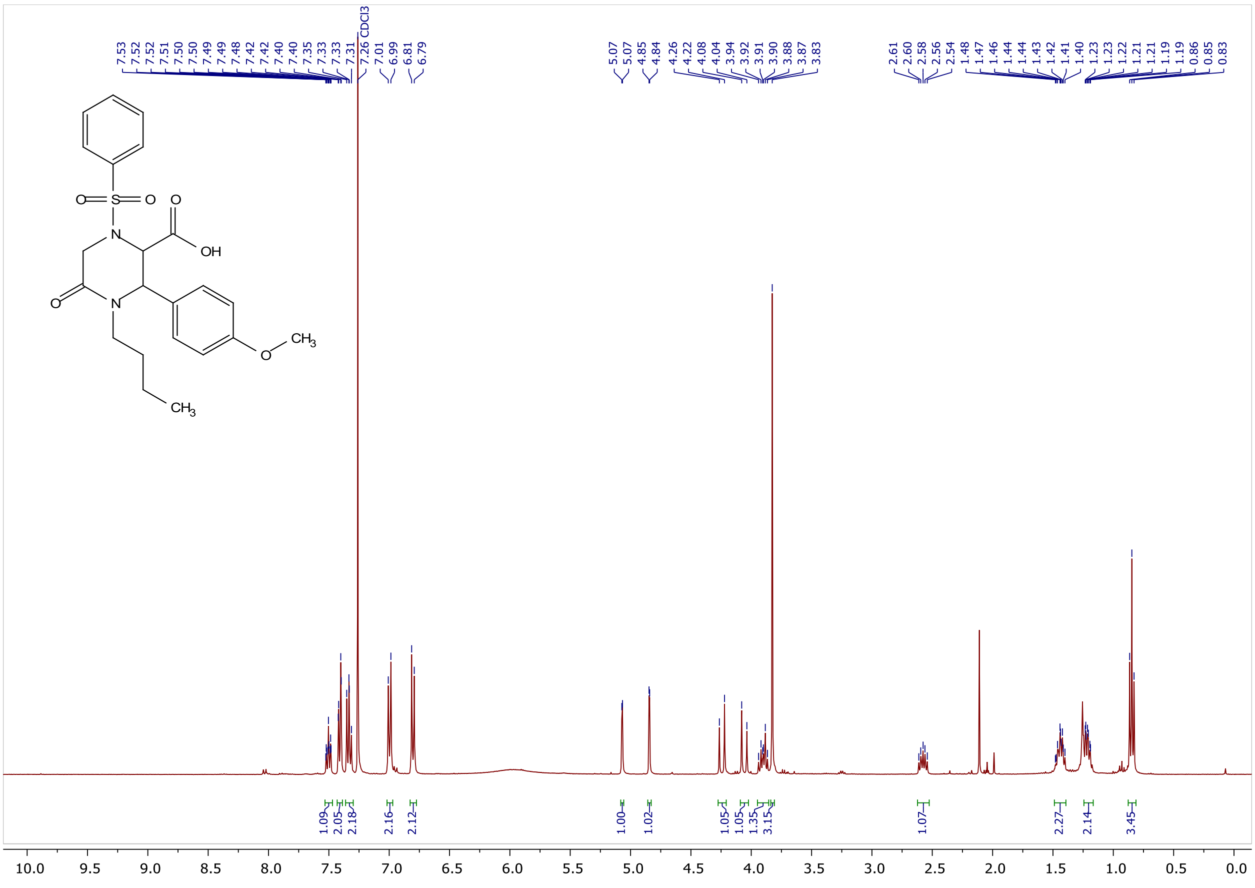

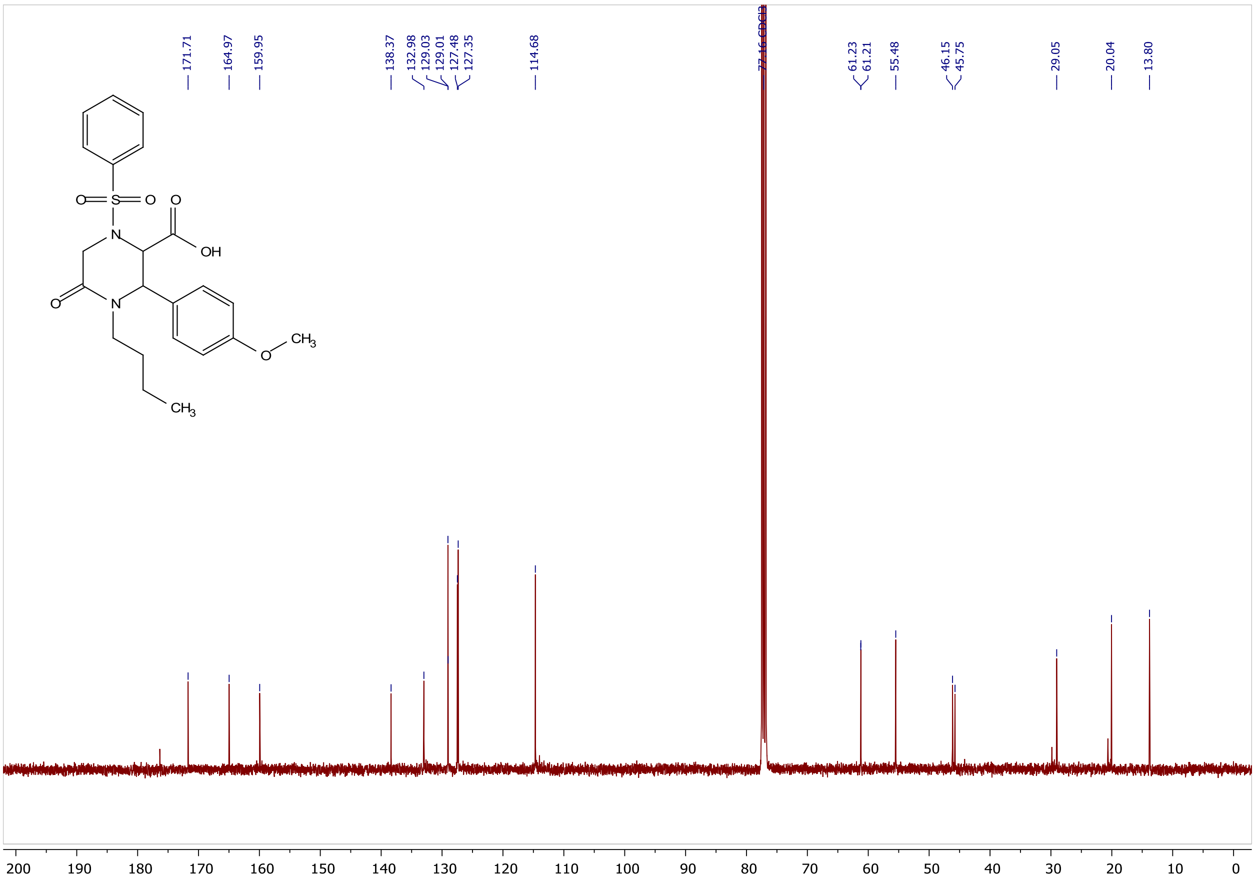
171.71
164.97
159.95
138.37
132.98
129.03
129.01
127.48
127.35
114.68
77.16 CDCl3
61.23
61.21
55.48
46.15
45.75
29.05
20.04
13.80
O
S
O
O
N
OH
O
N
CH3
CH3
200
190
180
170
160
150
140
130
120
110
100
90
80
70
60
50
40
30
20
10
0
